# Overlooked but not forgotten: the first new extant species of Hawaiian land snail described in 60 years, *Auriculellagagneorum* sp. nov. (Achatinellidae, Auriculellinae)

**DOI:** 10.3897/zookeys.950.50669

**Published:** 2020-07-20

**Authors:** Norine W. Yeung, John Slapcinsky, Ellen E. Strong, Jaynee R. Kim, Kenneth A. Hayes

**Affiliations:** 1 Bishop Museum, 1525 Bernice Street, 96817, Honolulu, Hawaii Bishop Museum Honolulu United States of America; 2 Florida Museum of Natural History, 1659 Museum Road, 32611, Gainesville, Florida Florida Museum of Natural History Gainesville United States of America; 3 National Museum of Natural History, Smithsonian Institution, PO Box 37012, MRC 163, 20013-7012, Washington, DC, USA Smithsonian Institution Washington United States of America

**Keywords:** gastropod, island, Oahu, Pacific, systematics

## Abstract

Recent surveys of Oahu’s Waianae Mountains uncovered a small, previously undescribed species of *Auriculella* that is conchologically similar to the three members of the *A.perpusilla* group all of which are endemic to the Koolau Mountain Range. However, sequence data demonstrate that the *perpusilla* group is not monophyletic. Moreover, the new species is not closely related to *A.perpusilla* or *A.perversa*, the only extant members of the group, but instead is sister to *A.tenella*, a species from the high spired *A.castanea* group. A neotype is designated for *A.auricula*, the type species of *Auriculella*; all members of the conchologically similar *perpusilla* group are anatomically redescribed; and lectotypes designated for *A.minuta*, *A.perversa*, and *A.tenella*. The new species is described and compared to the type of the genus, members of the *perpusilla* group, and the genetically similar species *A.tenella*.

## Introduction

Pacific Island land snails are among the most threatened faunas in the world, with more recorded extinctions since 1600 than any other group of animals (Régnier et al. 2009). Of the more than 25,000 islands spread across the Pacific, few have been extensively surveyed in modern times for their invertebrate fauna, and the estimates of extinction are probably a vast underestimate. Of the few islands and archipelagos that have been studied, like Hawaii, extinctions have been shown to be extensive (Régnier et al. 2015; Yeung and Hayes 2018). For example, as much as 93% of the endemic family Amastridae has been lost, and the other 12 families of land snails represented in Hawaii are not fairing much better (Yeung and Hayes 2018). Critical to understanding and slowing the rate of extinction is accurate and updated systematics and biogeography of land snails, and other understudied groups (Cardoso et al. 2011).

The Pacific Island family Achatinellidae is the second most diverse land snail family in the Hawaiian Islands with 209 species divided into five subfamilies, two of which, the Achatinellinae Gulick, 1873 and Auriculellinae Odhner, 1922, are endemic (Cooke and Kondo 1960). Historically the large and colorful Achatinellinae have garnered much attention and the lion’s share of molluscan conservation attention in Hawaii (Gulick 1872; Hadfield et al. 1993; Thacker and Hadfield 2000; Holland and Hadfield 2002, 2004, 2007; Erickson and Hadfield 2008; Hadfield and Saufler 2009; O’Rorke et al. 2015; Price et al. 2015, 2016a, b, 2018; Sischo et al. 2016), and include the only Hawaiian land snail species protected under the US Endangered Species Act (1981, 2013). However, the smaller, less colorful Auriculellinae, comprising 31 species in the genus *Auriculella* Pfeiffer, 1854 and one species in the genus *Gulickia* Cooke in Pilsbry & Cooke, 1915 have remained understudied and unprotected since the last revisions more than a century ago (Pilsbry and Cooke 1914–1916). Although fossils (Solem 1977; Severns 2009) and extinct species (Severns 2011) of Hawaiian land snails have continued to be described, no new extant species of native Hawaiian land snails have been described in more than 60 years. The last described extant Hawaiian land snail species was an achatinellid in the subfamily Tornatellidinae Cooke & Kondo, 1960, *Philopoasingularis* Cooke & Kondo, 1960 and the most recently described *Auriculella* species is *A.lanaiensis* Cooke in Pilsbry & Cooke, 1915.

Cooke and Kondo (1960) arranged *Auriculella* into four conchologically distinct groups: the *cerea* group from the southeastern islands of Hawaii, Lanai, Maui, and Molokai; and the *auricula*, *castanea*, and *perpusilla* groups which are all endemic to Oahu (Pilsbry and Cooke 1914–1916; Cooke and Kondo 1960). The *perpusilla* group (*A.perpusilla* Smith, 1873, *A.minuta* Cooke & Pilsbry in Pilsbry & Cooke, 1915, and *A.perversa* Cooke in Pilsbry & Cooke, 1915) contains the smallest species; all 6 mm or less in adult shell height. In addition to their small size these species have thin shells with 5 strongly convex whorls with low spires and weakly reflected apertures distinguishing them from the many-whorled, high-spired *castanea* group and the larger, thicker shelled *auricula* and *cerea* groups (Pilsbry and Cooke 1914–1916).

**Figure 1.**
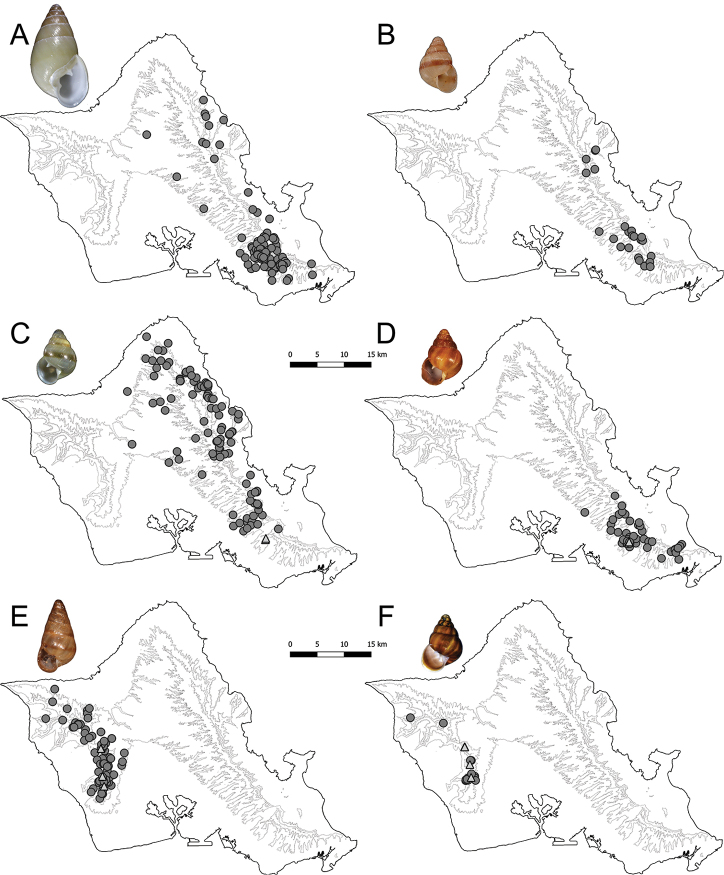
Distributional map of *Auriculella* spp. **A***Auriculellaauricula***B***Auriculellaminuta***C***Auriculellaperpusilla***D***Auriculellaperversa***E***Auriculellatenella* and **F***Auriculellagagneorum* sp. nov. Dark grey circle = historical; light grey triangle = since 2010.

In addition to their morphological similarity, the three species in the *perpusilla* group are all endemic to Oahu’s eastern Koolau range (Fig. 1B–D). Recent collecting in the island’s western Waianae range uncovered a previously undescribed species with features of shell size and shape that would place it in this group. The two mountain ranges are separated by a relatively dry, low elevation saddle 22 km long and 8 km wide and few land snail species have distributions in both ranges (Pilsbry and Cooke 1914–1916; Cowie et al. 1995). Specimens of the undescribed species were also found in samples collected prior to 1940, which were housed in the Bishop Museum (BPBM) and labelled by Y. Kondo as a potentially new species.

**Figure 2.**
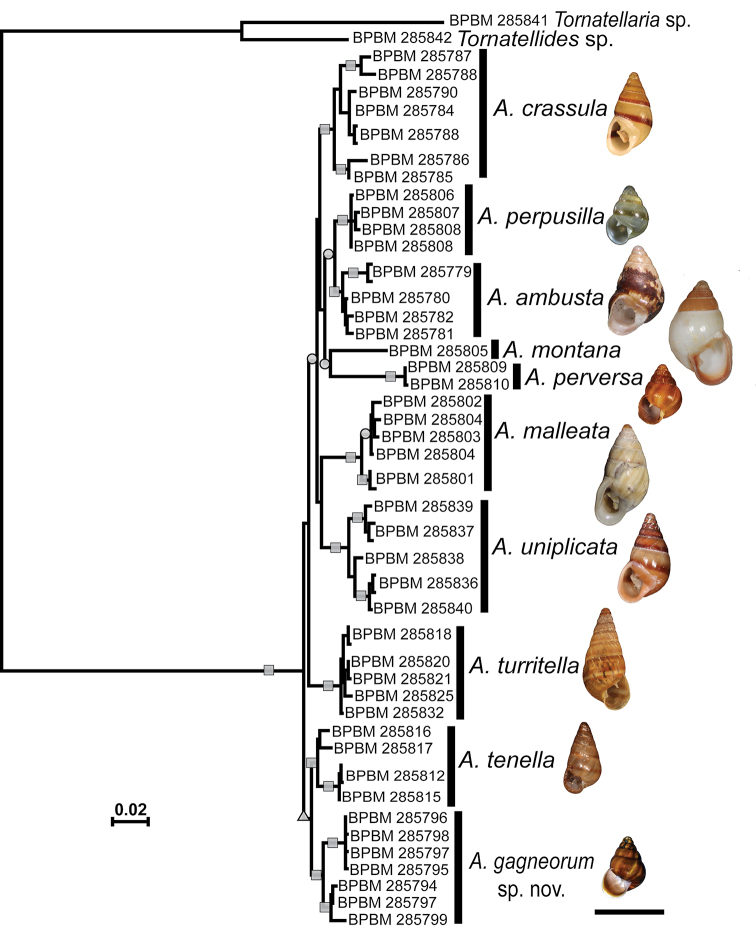
Phylogenetic tree of ten *Auriculella* spp. produced via Maximum Likelihood using a concatenated matrix composed of partial sequences of COI, 16S and 28S. Shapes on the nodes correspond to ML bootstrap values of 70–79 (triangle), 80–89 (circle), and 90–100 (square).

**Figure 3.**
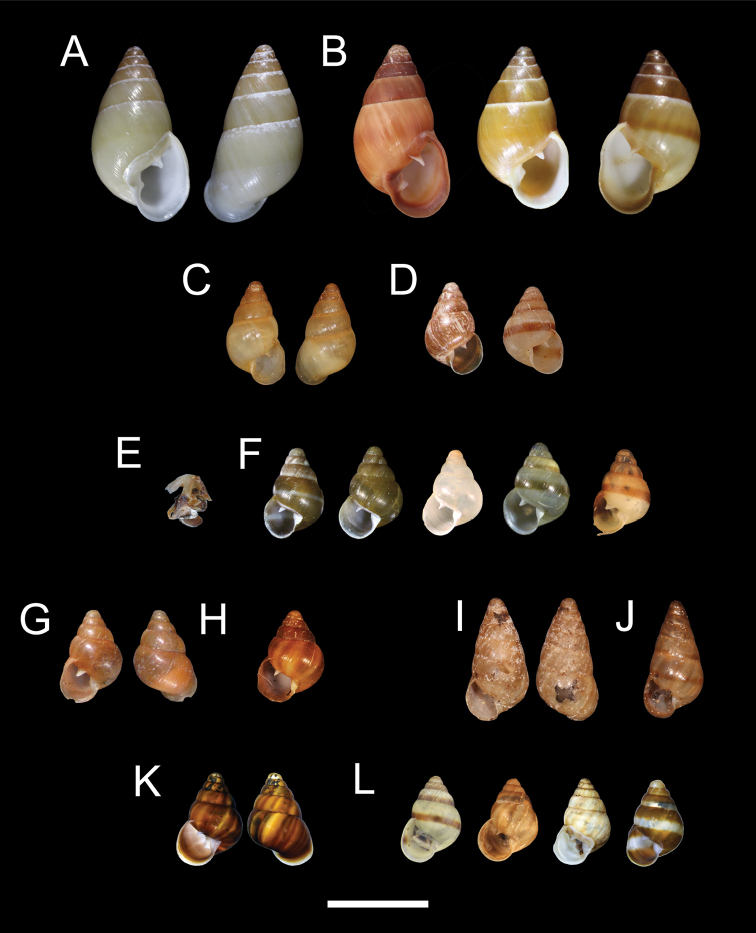
Comparative shell morphology of **A***Auriculellaauricula* neotype BPBM 18709 **B***Auriculellaauricula* shell variation (left to right) BPBM 12651, BPBM 12666 **C***Auriculellaminuta* lectotype BPBM 42377 **D***Auriculellaminuta* shell variation (left to right) BPBM12804, paralectotype MCZ 73037 **E***Auriculellaperpusilla* holotype MCZ 39912 **F***Auriculellaperpusilla* (left to right) BPBM 285806, BPBM 134341 (2 spm), BPBM 134280 white one, BPBM 122643, BPBM 13443 **G***Auriculellaperversa* lectotype BPBM 42384, 3 shells **H***Auriculellaperversa* shell variation (left to right) paralectotype ANSP 91817 **I***Auriculellatenella* lectotype BPBM 18943 **J***Auriculellatenella* shell variation paralectotype BPBM 109679 **K***Auriculellagagneorum* sp. nov. holotype (BPBM 285843) and **L***Auriculellagagneorum* sp. nov. paratypes, left to right (BPBM 285797, 285794, 285795, 285798). Scale bar: 5 mm.

Within *Auriculella*, intraspecific shell morphology varies and may often overlap interspecifically, making species delineation based on conchology alone difficult (Pilsbry and Cooke 1914–1916). As such, additional morphological and molecular data (e.g., DNA and RNA sequences) are necessary to distinguish among closely related species. The reproductive anatomy of only a few *Auriculella* species is known. Pilsbry and Cooke (1915 on plate 22) figured *A.pulchra* Pease, 1868 (figs 1, 2); *A.cerea* (Pfeiffer, 1855) (fig. 3); and *A.armata* (Mighels, 1845) as *A.westerlundiana* Ancey, 1889 (fig. 6). The reproductive anatomy of the type species of the genus, *Auriculellaauricula* (Férussac, 1821), was figured and described by Cooke and Kondo (1960: figs 113a–d, 114a–c) who also dissected 22 other species but figured only *A.castanea* (Pfeiffer, 1853) (Cooke and Kondo 1960: fig. 114d). The reproductive anatomy of the other species has never been figured or described, including all members of the *perpusilla* group. As part of a broader project whose aim is to fully revise the systematics of the Achatinellidae, we use an integrative approach using data from conchology, radula, reproductive system, and DNA sequences, to redescribe *A.auricula*, the type species of the genus and all members of the *perpusilla* group (*A.perpusilla*, *A.minuta*, *and A.perversa*). We also describe a new species, *A.gagneorum* sp. nov., based on recently collected material and from lots housed in the Bishop Museum. Relationships of the taxa traditionally relegated to the *perpusilla* group, and of the conchologically similar *A.gagneorum* sp. nov., are explored with a mitochondrial and nuclear gene dataset. To enhance the stability of the nomenclature, we designate a neotype for *A.auricula* and lectotypes for members of the *perpusilla* group.

## Materials and methods

As part of a long-term study of extant Hawaiian land snails, our team has surveyed more than 1000 sites across the six largest Hawaiian Islands (Kauai, Oahu, Maui, Molokai, Lanai, Hawaii). The targeted locations were those that historically supported snail populations, as well as more remote areas with remnant native vegetation that were often accessible only by helicopter. Surveys followed Durkan et al. (2013) and consisted of leaf litter sampling and hand collecting for at least one-person-hour by a minimum of two experienced malacologists in quadrats of at least 10 m^2^, but up to 100 m^2^, terrain permitting. GPS coordinates were collected at every survey site and coordinates were estimated for historical BPBM specimen records using locality, field notes, maps, and other descriptions. The precise locations (e.g., GPS coordinates) for material listed are not provided here for conservation purposes but are kept in the State of Hawaii Department of Land and Natural Resources Snail Extinction Prevention Program and Bishop Museum Malacology databases. Distributional maps were created using QGIS v3.8.2 (QGIS 2019) and used to show historical and current distributions of the species treated herein.

Newly collected material was photographed, flash boiled (Fukuda et al. 2008), and then fixed in 95% ethanol, after which a small piece of foot tissue was removed for DNA extraction. The remaining soft tissues were preserved in 80% ethanol, and dissections were performed on preserved specimens submerged in 75% ethanol. Shells and reproductive anatomy were photographed with digital single-lens reflex cameras (e.g., Cannon EOS 7D) attached to a dissecting microscope. Photographs of reproductive anatomy were traced in Photoshop to produce line drawings. Shell measurements were made using an ocular micrometer and each measurement was repeated three times and averaged for 50 specimens per species. Shell measurements, shell height **(H)**, shell width **(W)**, aperture height **(AH)**, aperture width **(AW)**, and number of whorls **(WH)** were made following Slapcinsky and Kraus (2016: 30, fig. 1). All pertinent type and comparative material were examined and photographed. Locality and collector information of materials examined were listed as verbatim. Materials examined for the new species is provided in the text and all others can be found in Suppl. material 1. Museum collections are abbreviated:

**ANSP**Academy of Natural Sciences, Philadelphia;

**BPBM**Bernice P. Bishop Museum, Honolulu;

**MCZ**Museum of Comparative Zoology, Cambridge;

**MNHN**Muséum national d’Histoire naturelle, Paris;

**NMW**National Museum of Wales, Cardiff;

**RBINS**Royal Belgian Institute of Natural Sciences, Brussels;

**SMF**Naturmuseum Senckenberg, Frankfurt.

Radulae were tissue-digested in 180 µL of T1 lysis buffer (Macherey-Nagel) containing 20 mg/mL of Proteinase-K and rinsed in de-ionized water. Cleaned radulae were mounted directly on carbon adhesive tabs attached to aluminum stubs, which were then coated with 25–30 nm gold/palladium (60/40) and photographed using an Apreo scanning electron microscope (FEI Company) at the National Museum of Natural History, Washington.

Total genomic DNA (gDNA) was extracted from an approximately 1 mm^3^ piece of foot tissue using the Macherey-Nagel NucleoSpin Tissue Kit following the manufacturer’s instructions, with the exception that elution was with 60 µl of elution buffer supplied with the kit, and gDNA stored at -20 °C prior to amplification via the polymerase chain reaction (PCR).

Portions of two mitochondrial genes, 16S ribosomal DNA (rDNA) and cytochrome *c* oxidase subunit I (COI), and the nuclear encoded 28S rDNA were amplified using primers listed in Table 1. Reactions were carried out in 25 µl volumes containing 1–2 µl template DNA and a final concentration of 1 U of MangoTaq DNA polymerase (Bioline), 1X reaction buffer, 0.2 mM each dNTP, 2.5 mM MgCl_2_ and 0.75 µM of each primer, 10 µg BSA, and 0.5% DMSO. Cycling parameters were one cycle of 5 min at 95 °C, 1 min at 44–48 °C, 2 min at 72 °C, followed by 34 cycles of 95 °C, 46–50 °C, and 72 °C for 30 sec each, and a final extension of 5 min at 72 °C. A final 4 °C incubation of 30 min terminated each reaction (Table 1). The amount and specificity of amplifications were verified via agarose electrophoresis and single product amplicons were cycle sequenced using the ABI BigDye terminator kits (Perkin-Elmer Applied Biosystems, Inc.). Sequences were electrophoresed and analyzed on an ABI 3730XL (Perkin-Elmer Applied Biosystems, Inc.) at either the University of Hawaii’s Advanced Studies in Genomics, Proteomics, and Bioinformatics facility or Eurofins Genomics, LLC. All loci were initially sequenced in one direction, and any unique haplotypes sequenced in both directions. The COI fragment was sequenced for all individuals, and subsets of these were selected based on unique COI haplotypes and sequenced for 16S and 28S. Due to lower variability in the other two loci, not all individuals with a unique COI haplotype were sequenced for all other loci. All sequences have been uploaded to the Barcode of Life Data System (BoLD; https://doi.org/10.5883/DS-AURICOI) and to GenBank (Accession numbers MT519807–MT519913; Table 2)

**Table 1.** Primers and PCR annealing temperatures.

Locus	T_A_ °C	Primers F/R
COI	44-46	LCO1490/HCO2198 (Folmer et al. 1994)
16S	48-50	16Sar/16S2 (Palumbi 1996; Garey et al. 1998)
28S	46-48	LSU2/LSU5 (Wade et al. 2006)

**Table 2.** Museum catalog numbers for specimens used in genetic analysis with numbers of specimens from which shell measurements, reproductive anatomy and radular morphology were obtained. Catalog numbers (BPBM) are for lots from which specimens were sequenced (N = number of individuals sequenced from each lot).

Genus	Species	Island	BPBM	COI	16S	28S	Shell measurements	Reproductive system	Radula
*Auriculella*	*ambusta*	Oahu	BPBM 285779 (1)	MT519807	–	MT519879	–	–	–
		Oahu	BPBM 285779 (1)	MT519811	MT519861	MT519880	–	–	–
		Oahu	BPBM 285780 (1)	MT519808	MT519860	–	–	–	–
		Oahu	BPBM 285781 (1)	MT519809	–	–	–	–	–
		Oahu	BPBM 285782 (2)	MT519810	–	–	–	–	–
	*auricula*	Oahu	BPBM 119141	–	–	–	–	1	–
		Oahu	BPBM 119157	–	–	–	–	–	1
		Oahu	BPBM 119172	–	–	–	–	–	2
		Oahu	BPBM 119202	–	–	–	–	1	–
		Oahu	BPBM 12651	–	–	–	21	–	–
		Oahu	BPBM 12666	–	–	–	26	–	–
		Oahu	BPBM 164138	–	–	–	–	–	1
		Oahu	BPBM 164143	–	–	–	–	–	1
		Oahu	BPBM 189709	–	–	–	–	–	1
		Oahu	BPBM 189710	–	–	–	–	–	1
		Oahu	BPBM 190854	–	–	–	–	–	1
		Oahu	BPBM 285783	–	–	–	3	–	–
	*crassula*	Maui	BPBM 285784 (1)	MT519819	–	MT519888	–	–	–
		Maui	BPBM 285785 (1)	MT519814	–	MT519883	–	–	–
		Maui	BPBM 285786 (1)	MT519813	MT519863	MT519882	–	–	–
		Maui	BPBM 285787 (3) BPBM 285793 (3) BPBM 285791 (5) BPBM 285792 (1)	MT519816	MT519865	MT519885	–	–	–
		Maui	BPBM 285788 (1)	MT519815	MT519864	MT519884	–	–	–
		Maui	BPBM 285788 (2)	MT519817		MT519886	–	–	–
		Maui	BPBM 285789 (4) BPBM 285788 (2)	MT519818	–	MT519887	–	–	–
		Maui	BPBM 285790 (3)	MT519812	MT519862	MT519881	–	–	–
	*gagneorum* sp. nov.	Oahu	BPBM 174233	–	–	–	7	–	–
		Oahu	BPBM 21823	–	–	–	40	–	–
		Oahu	BPBM 285794 (1) BPBM 285844 (1) BPBM 285796 (1)	MT519823	–	MT519891	–	–	–
		Oahu	BPBM 285795 (1)	MT519826	–	–	–	–	–
		Oahu	BPBM 285796 (1) BPBM 285794 (1)	MT519820	MT519866	MT519889	–	–	–
		Oahu	BPBM 285797 (1)	MT519821	MT519867	MT519890	–	1	–
		Oahu	BPBM 285797 (1)	MT519824	–	MT519892	–	–	–
		Oahu	BPBM 285798 (1)	MT519825	–	–	–	1	1
		Oahu	BPBM 285799 (1) BPBM 285800 (2) BPBM 285843 (1)	MT519822	MT519868	–	–	–	–
	*malleata*	Oahu	BPBM 285801 (1)	MT519830	–	MT519894	–	–	–
		Oahu	BPBM 285801 (1)	MT519831	–	MT519895	–	–	–
		Oahu	BPBM 285802 (1)	MT519829	MT519869	MT519893	–	–	–
		Oahu	BPBM 285803 (3)	MT519828	–	–	–	–	–
		Oahu	BPBM 285804 (1)	MT519832	MT519870	MT519896	–	–	–
		Oahu	BPBM 285804 (1)	MT519827	–	–	–	–	–
	*minuta*	Oahu	BPBM 12799	–	–	–	14	–	–
		Oahu	BPBM 12804	–	–	–	25	–	–
		Oahu	BPBM 170304	–	–	–	10	–	–
		Oahu	BPBM 98043	–	–	–	–	–	2
		Oahu	BPBM 99164	–	–	–	–	2	–
*Auriculella*	*minuta*	Oahu	BPBM 99164	–	–	–	–	–	3
	*montana*	Oahu	BPBM 285805 (1)	MT519833	–	–	–	–	–
	*perpusilla*	Oahu	BPBM 122643	–	–	–	15	–	–
		Oahu	BPBM 134280	–	–	–	15	–	–
		Oahu	BPBM 134431	–	–	–	15	–	–
		Oahu	BPBM 15048	–	–	–	4	–	–
		Oahu	BPBM 285806 (1)	MT519837	MT519872	MT519898	–	–	1
		Oahu	BPBM 285807 (1) BPBM 285808 (1)	MT519835	MT519871	MT519897	–	–	–
		Oahu	BPBM 285808 (1)	MT519834	–	–	–	–	–
		Oahu	BPBM 285808 (1)	MT519836	–	–	–	–	–
		Oahu	BPBM 90853	–	–	–	–	–	2
		Oahu	BPBM 93626	–	–	–	–	3	2
	*perversa*	Oahu	BPBM 12798	–	–	–	15	–	–
		Oahu	BPBM 164180	–	–	–	–	–	2
		Oahu	BPBM 22767	–	–	–	34	–	–
		Oahu	BPBM 285809 (1)	MT519839	–	–	–	–	–
		Oahu	BPBM 285810 (2)	MT519838	–	–	–	1	–
		Oahu	BPBM 97904	–	–	–	–	1	2
	*tenella*	Oahu	BPBM 125606	–	–	–	7	–	–
		Oahu	BPBM 162827	–	–	–	–	1	–
		Oahu	BPBM 18943	–	–	–	1	–	–
		Oahu	BPBM 211034	–	–	–	–	1	1
		Oahu	BPBM 285811	–	–	–	2	–	–
		Oahu	BPBM 285812 (1)	MT519841	MT519874	MT519900	–	–	–
		Oahu	BPBM 285812 (1) BPBM 285813 (1) BPBM 285814 (1)	MT519842	MT519875	–	–	–	–
		Oahu	BPBM 285815 (1)	MT519843	–	–	–	–	1
		Oahu	BPBM 285816 (1)	MT519840	MT519873	MT519899	–	–	–
		Oahu	BPBM 285817 (1)	MT519844	MT519876	MT519901	–	–	–
		Oahu	BPBM 33194	–	–	–	42	–	–
	*turritella*	Oahu	BPBM 285818 (1)	MT519845	–	MT519902	–	–	–
		Oahu	BPBM 285818 (1) BPBM 285819 (1) BPBM 285829 (2) BPBM 285832 (1) BPBM 285833 (1)	MT519850	–	MT519904	–	–	–
		Oahu	BPBM 285821 (1)	MT519847	–	–	–	–	–
		Oahu	BPBM 285823 (1) BPBM 285824 (3) BPBM 285822 (1) BPBM 285820 (1) BPBM 285826 (1) BPBM 285827 (1) BPBM 285828 (1)	MT519849	–	MT519903	–	–	–
		Oahu	BPBM 285825 (1) BPBM 285830 (1) BPBM 285831 (2) BPBM 285834 (1)	MT519848	–	–	–	–	–
		Oahu	BPBM 285832 (1) BPBM 285835 (1)	MT519846	–	–	–	–	–
	*uniplicata*	Maui	BPBM 285836 (1)	MT519851	–	MT519905	–	–	–
		Maui	BPBM 285836 (1)	MT519852	–	MT519906	–	–	–
		Maui	BPBM 285837 (1)	MT519853		MT519907	–	–	–
		Maui	BPBM 285837 (1)	MT519856	–	MT519910	–	–	–
		Maui	BPBM 285838 (2)	MT519855	–	MT519909	–	–	–
		Maui	BPBM 285839 (1)	MT519857	–	MT519911	–	–	–
		Maui	BPBM 285840 (2)	MT519854	–	MT519908	–	–	–
*Tornatellaria*	sp.	Maui	BPBM 285841 (1)	MT519858	MT519877	MT519912	–	–	–
*Tornatellides*	sp.	Molokai	BPBM 285842 (1)	MT519859	MT519878	MT519913	–	–	–

Electropherograms were checked for errors, edited, and assembled using Geneious Prime 2019 (http://www.geneious.com/). Sequences of COI were unambiguously aligned using MAFFT ver. 7.388 with the iterative refinement method E-INS-I (Katoh and Standley 2013) implemented in Geneious Prime 2019. Alignments where checked against amino acid sequences as references. Ribosomal genes were aligned using MAFFT and refined using Gblocks ver. 0.91b (Castresana 2000). Refinement of the 16S and 28S alignments in Gblocks removed regions of ambiguous homology created by the addition of gaps during initial alignment and the hypervariable nature of some regions. Phylogenetic analyses were done with and without these regions to evaluate their impact. Sequence alignments were concatenated in Geneious Prime and exported as phylip files for phylogenetic analysis.

Phylogenetic reconstruction was conducted using maximum likelihood (ML) in IQ-TREE ver. 1.6.12 (Nguyen et al. 2015). The best-fit partitioning scheme and the most appropriate substitution model for each partition were estimated using the integrated ModelFinder algorithm (Kalyaanamoorthy et al. 2017) and partition models (Chernomor et al. 2016). Nodal support was estimated with 5,000 ultra-fast bootstrap replicates (Hoang et al. 2018).

To corroborate species delineation based on conchological and anatomical analyses and phylogenetic reconstruction, we used the DNA barcode-based species identification method implemented in SpeciesIdentifier ver. 1.8 (Meier et al. 2006).

Museum catalog numbers for specimens used in DNA analysis with numbers of specimens from which shell measurements, reproductive anatomy, and radular morphology were obtained, are listed in Table 2.

## Results

Recent surveys recorded extant populations of two of the three species within the *perpusilla* group: *A.perpusilla* and *A.perversa* (Fig. 1C, D, respectively) and a new species with similar shell morphology, *A.gagneorum* sp. nov. (Fig. 1F). No populations of *A.auricula* (type species of the genus, Fig. 1A) or *A.minuta* (Fig. 1B) were recorded in our surveys and both species may be extinct.

The 104 snails representing ten *Auricullela* species and two outgroup taxa (*Tornatellaria* sp. and *Tornatellides* sp.) sequenced for this study produced 53 COI haplotypes, 19 and 35 sequences for 16S and 28S, respectively. Alignments for each locus were 654 bp for COI, 464 bp for 16S and 539 bp for 28S, making the concatenated dataset of 53 individuals 1657 bp with 223 parsimony informative sites. Sixteen individuals were represented by all three loci, while three individuals had only COI and 16S, 19 with COI and 28S, and 15 with only COI. The best-fit partitioning scheme used distinct models for each locus with the best-fit models being K3Pu+F+I+G4, TPM2u+F+G4, and TIM3+F for COI, 16S, and 28S respectively.

The ML tree constructed from the concatenated dataset produced a well-resolved tree with all conchologically defined taxa recovered in strongly supported clades (Fig. 2). None of the groupings suggested by Cooke and Kondo (1960) based on gross shell morphology were recovered in the ML tree. As such, *A.perpusilla* and *A.perversa*, previously referred to the *perpusilla* group were recovered in unrelated clades with each as sister to much larger shelled species, *A.ambusta* and *A.montana*, respectively. Similarly, the new species *Auriculellagagneorum* sp. nov. was recovered as sister to *A.tenella* and not close to *A.perpusilla* or *A.perversa* with which it was previously confused.

The best match/best close match criteria (Meier et al. 2006) applied to all 53 COI haplotypes successfully matched all sequences in the correct conspecific clusters within a 3–4% threshold consistent with conchologically and phylogenetically recognized clades. Correct identifications with both approaches was 94.33%, with the other 5.66% (three sequences) lacking any conspecific sequences with which to cluster. These included the two outgroup taxa and *A.montana*, all of which were represented by a single sequence.

## Systematics

### Class Gastropoda Cuvier, 1795

**Subclass Heterobranchia Burmeister, 1837**

**Order Stylommatophora A. Schmidt, 1855**

**Superfamily Pupilloidea W. Turton, 1831**

**Family Achatinellidae Gulick, 1873**

**Subfamily Auriculellinae Odhner, 1921**

#### Genus Auriculella

Taxon classificationAnimalia

Pfeiffer, 1854

060BE0C9-E3E4-54B2-A97D-493C61CA3B97

##### Type species.

*Partulaauricula* Férussac, 1821 by subsequent designation (Gulick 1873).

##### Diagnosis.

Small to moderately sized Achatinellidae, 4 to 12 mm in adult shell height. Shells either dextral or sinistral, taller than wide, with a strong parietal lamella. Juvenile shells have two columellar lamellae, one or both of which are lacking in adults. Phallus with an epiphallus and a nearly apical appendix. Phallus retractor muscle inserted apically on the epiphallus and not secondarily attached to the appendix. Members of *Auriculella* are the only achatinellids known to have an epiphallus. All *Auriculella* species are oviparous (Pilsbry and Cooke 1914–1916).

#### Auriculella auricula

Taxon classificationAnimalia

(Férussac, 1821)

B02BC15C-DA25-5967-9F28-6F546C7F134E

**Figure 4.**
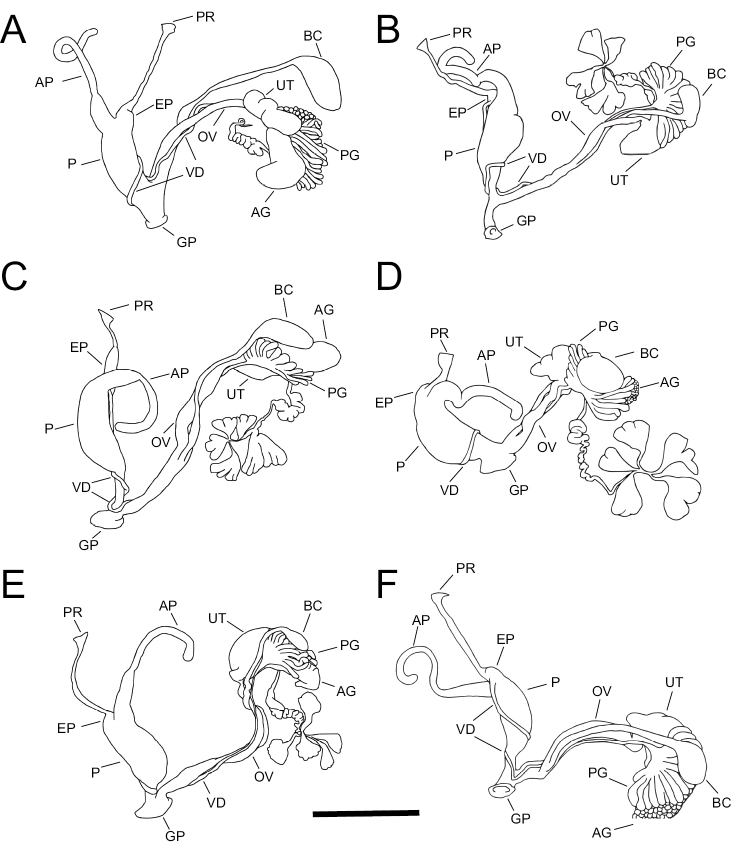
Comparative reproductive anatomy of **A***Auriculellaauricula*BPBM 119141 **B***Auriculellaminuta*BPBM 99146 **C***Auriculellaperpusilla*BPBM 93626 **D***Auriculellaperversa*BPBM 97904 **E***Auriculellatenella*BPBM 211034 **F***Auriculellagagneorum* sp. nov. paratype BPBM 285800. Abbreviatons for reproductive structures are: AG = albumen gland; AP = penial appendix; BC = bursa copulatrix; EP = epiphallus; GP = gonopore; P = penis; OV = free oviduct; PG = prostate gland; PR = penial retractor muscle; UT = uterus; VD = vas deferens. Scale bar: 1mm.

**Figure 5.**
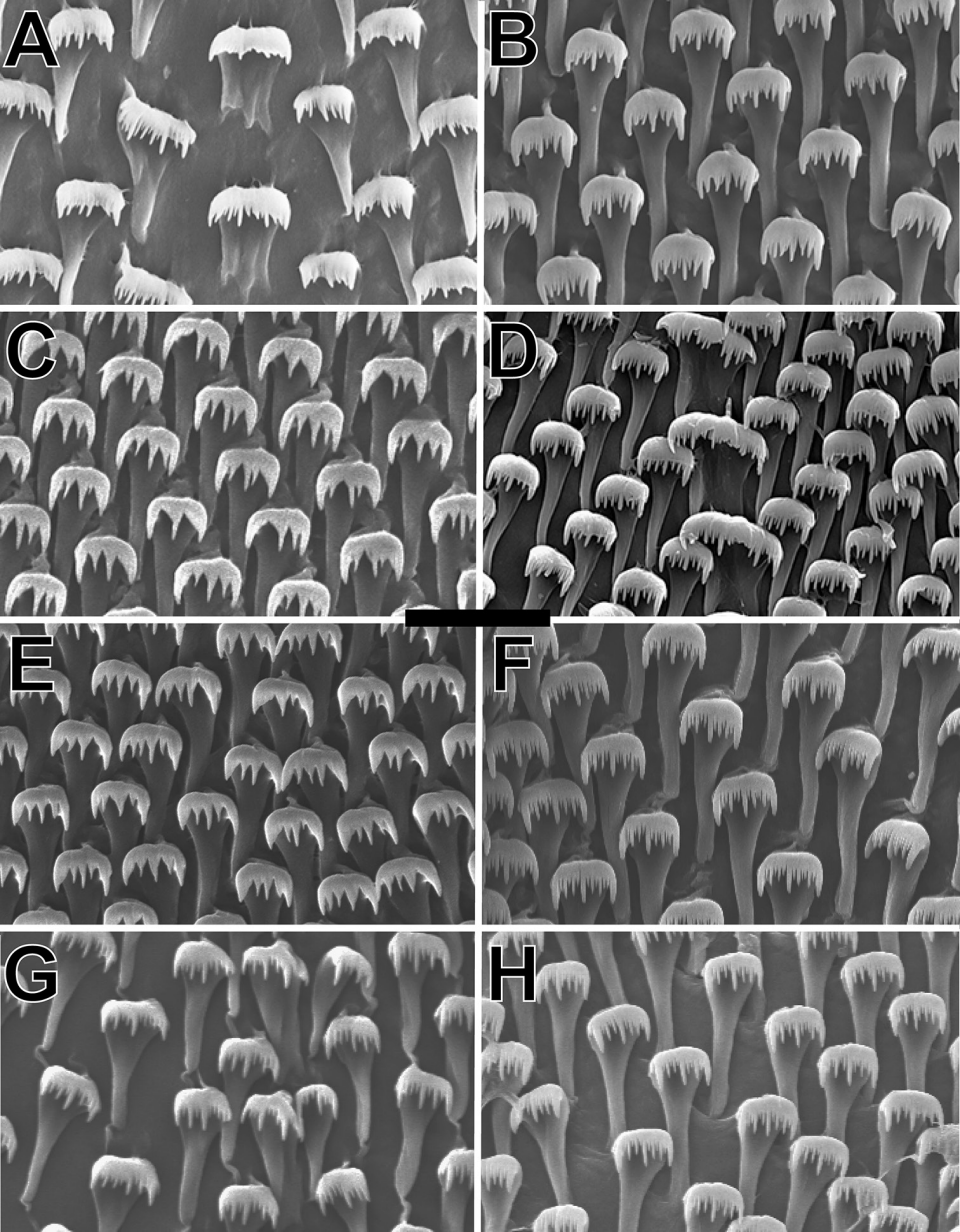
Comparative radular morphology of **A***A.auricula* (irregular rachidian and rastriform marginal teeth) **B***A.auricula* (rastriform marginal teeth) **C***A.minuta* (rastriform marginal teeth) **D***A.perpusilla* (irregular rachidian and rastriform marginal teeth) **E***A.perversa* (rastriform marginal teeth) **F***Auriculellatenella* (rastriform marginal teeth) **G***A.gagneorum* sp. nov. (irregular rachidian and rastriform marginal teeth) **H***A.gagneorum* sp. nov. (rastriform marginal teeth). Scale bar: 10 μm. Scale bar: 10 μm.

Figures 1A , 3A, B , 4A , 5A

Partula auricula Férussac, 1821: 66.
Auriculella auricula – Gulick 1872: 222; Gulick 1873: 91; Pilsbry and Cooke 1915: 78–80, pl. 24, figs 1–10; Cooke and Kondo 1960: 270–272, figs 113a–e, 114a–c; Cowie et al. 1995: 75; Severns 2011: 206, pl. 80, fig. 2.

##### Type material.

***Neotype***: USA • 1; H = 8.7 mm, W = 4.2 mm, AH = 4.6 mm, AW = 3.3 mm, with 6.4 WH; Honolulu County, Oahu, Koolau Mountains, Tantalus; 09 Jun 1943; Y. Tanada leg.; BPBM 189709.

##### Type locality.

“Sans doute les îles de la mer du Sud?” [without doubt the south sea islands?]; colloquially “sans doute” means probably; here restricted to Tantalus.

##### Diagnosis.

***Shell*.** Shell dextral or sinistral with flat-sided whorls and an obtuse apex, H = 8.0 ± 0.4 mm, W = 4.3 ± 0.2 mm, WH 6.0 ± 0.2, AH = 4.1 ± 0.2 mm, AW = 3.1 ± 0.2 mm (*N* = 50; Table 2). Columella with a single strong lamella and without an axial ridge. Parietal lamella is strong and smooth and not undulate, extending 0.3 to 0.7 whorls into the aperture. Shell color is tan, brown, or yellowish, often with a single narrow brown or white band (Fig. 3B). White bands are sometimes bordered by two darker brown bands and apical whorls are often darker brown. Lip reflected, thickened, white or brown in color.

***Reproductive system*.** Phallus retractor muscle relatively long, attached apically to a short but well-defined epiphallus (Fig. 4A). Appendix is longer than the phallus and about ⅔ the diameter of the phallus at its attachment. The appendix narrows abruptly at ⅕ its length and remains narrow to its terminus. Phallus is broad, narrowing only slightly apically and basally. Atrium is relatively short and broad. Vagina is about ⅓ the length of the phallus.

***Radula*.**Radula with an irregular rachidian flanked on either side by rastriform marginal teeth, as diagnostic of the family (Fig. 5A). Each tooth has a long narrow base that expands slowly for ¾ of the length of the tooth before reaching the forward curving cusps, which comprise the remaining ¼ of the tooth. There are three long cusps at mesocone, endocone, and ectocone positions with two or more alternating larger and smaller cusps intercalated between them. Number of teeth per row range from 177 to 183 (*N* = 6; Table 2).

##### Distribution and ecology.

*Auriculellaauricula* is endemic to Oahu’s Koolau Mountains (Fig. 1A), historically found across the range at elevations from 61 m to 305 m. The species is arboreal and found on vegetation including: *Cordyline* sp. *Freycinetiaarborea*, *Metrosiderospolymorpha*, *Canna* sp. (BPBM 34025, 49056, 51405), *Aleuritesmoluccanus*, *Psychotria* sp., *Zingiber* sp., *Psidiumcattleyanum*, *Musa* sp., *Asplenium* sp., and unspecified ferns and shrubs. The species has also been recorded on the ground under stones, logs and dead leaves. Live specimens recorded in the BPBM collection were last collected by Y. Kondo in 1946 from Palolo Valley; the species has not been recorded in recent surveys and is considered here possibly extinct.

##### Remarks.

In the original description, Férussac (1821) provided measurements for a single shell of three lines (6.8 mm) in height and 1¾ lines (4.0 mm) in width. His collection is housed in MNHN where there are two lots labelled *A.auricula* that are attributed to Férussac. The first (MNHN IM-2000-34306, 34307, 34308) is from Férussac’s collection but does not contain original labels. The three dextral shells are identified as *A.auricula* from the Mariana Islands, but they are not *A.auricula* and instead appear to be a gerontic adult and two juveniles similar to *Auriculellaambusta*, a species not found on the same mountain range as *A.auricula*. The other lot (MNHN IM-2014-7009) is from the Deshayes collection. Its source is unknown but probably came from Férussac whose specimens Deshayes used to complete Férussac’s “Histoire naturelle des mollusques terrestres et fluviatiles” after Férussac’s death in 1836. The lot contains six specimens of *Auriculellapulchra*, two of which are sinistral and all of which are larger than 6.8 mm. The two lots are not consistent with Férussac’s description, and we exclude these lots as possible syntypes of *A.auricula*. We have not located any other type material of *A.auricula* and we consider the types to be lost. Stabilizing the nomenclature of this species is important because it is the type species of the genus *Auriculella*, a genus with many similar but conchologically variable and poorly resolved species, nearly all of which are highly endangered. We designate BPBM 18709 (Fig. 3A) from Tantalus, Oahu as neotype of *Auriculellaauricula* to stabilize the taxonomic status and type locality of the species as well as the genus *Auriculella*. The neotype matches Férussac’s original description in having an acute ovoid shell with an obtuse apex, strong parietal lamella, and single columellar lamella. The color of the neotype is more tan than yellowish as described in the original description but the species is known to be polymorphic for shell color and pattern as well as chirality. The shell used in Férussac’s description was sinistral while the neotype is dextral. We chose a dextral specimen with slightly different coloration because it was used by Cooke and Kondo (1961) to describe the nervous system and reproductive anatomy of *Auriculellaauricula* thus clearly defining the species as well as the genus. The other four specimens from BPBM 189709 are re-cataloged as BPBM 285783. One of these is a broken shell presumably corresponding to the animal dissected by Cooke and Kondo (1961).

#### Auriculella minuta

Taxon classificationAnimalia

Cooke & Pilsbry, 1915

FE326122-6D99-5788-AB97-760EEEE0A7B9

Figures 1B , 3C, D , 4B , 5B

Auriculella minuta Cooke & Pilsbry in Pilsbry & Cooke, 1915: 90, pl. 25, figs 5–9; Cowie et al. 1995: 76; Johnson 1996: 190; Severns 2011: 210, pl. 82, fig. 1.

##### Type material.

***Lectotype***: USA • 1, H = 4.9 mm, W = 2.8 mm, AH = 2.2 mm, AW = 1.7 mm, WH = 5.7.; Honolulu County, Oahu, Koolau Mountains, Nuuanu; Nuuanu Valley Ridge 7, east, on ti, lehua, *Passiflorafoetida*; Cooke leg.; BPBM 42377, here designated

***Paralectotypes***: USA – Honolulu County, Oahu, Koolau Mountains • 1; Nuuanu Valley; Cooke leg.; BPBM 42377 • 1; Nuuanu Ridge; BPBM 13034 • 2; Nuuanu; BPBM 42379 • 1; Nuuanu Ridge; Cooke leg.; BPBM 42380 • 33; Nuuanu Ridge; Cooke leg.; BPBM 4238 • 1; Nuuanu Valley; Cooke leg.; BPBM 42382 • 82; Nuuanu Valley; Cooke leg.; BPBM 42383 • 5, Palolo Valley; Lyman leg.; BPBM 12808

***Paralectotypes not examined*.**ANSP 91816 (11 spm), ANSP 113294 (10 spm), MCZ 73037 (5 spm), SMF 7127 (4 spm), BPBM 12808 (5 spm).

***Possible paralectotype*.** USA – Honolulu County, Oahu, Koolau Mountains • 6; Palolo Valley; BPBM 16435.

##### Type locality.

Hawaiian Islands, Oahu, Nuuanu. See

##### Remarks.

##### Diagnosis.

***Shell*.** Shell dextral, H = 4.4 ± 0.18 mm, W = 2.7 ± 0.11 mm, WH = 5.1 ± 0.08, AH = 1.9 ± 0.11 mm, AW = 1.3 ± 0.08 mm (*N* = 50; Table 2). Whorls inflated. Columella in juveniles with a strong lamella that is reduced and covered by a thickening of the inner edge of the lip in adults. Some adults show a short projection or angulation where the columellar lamella was located. Adult columella reflected, without an axial ridge. Parietal lamella is smooth and not undulate, extending 0.2 to 0.5 whorls into the aperture. Shell color is pale tan or dark brown, with or without a single peripheral color band of pale tan or dark brown (Fig. 3D, MCZ 73037).

***Reproductive system*.** Phallus retractor muscle relatively long, attached apically to a short but well-defined epiphallus (Fig. 4B). Appendix is nearly equal in length to the phallus. Appendix the diameter of the phallus at its attachment, narrowing abruptly at ⅓ its length and remaining narrow to its terminus. Apical ¾ of the phallus is broad, basal ¼ narrows abruptly remaining narrow to the junction with the moderately long atrium. Vagina is long and nearly half the length of the phallus.

***Radula*.**Radula with an irregular rachidian flanked on either side by rastriform marginal teeth, as diagnostic of the family (Fig. 5B). Each tooth has a long narrow base that expands slowly for ¾ of the length of the tooth before reaching the forward curving cusps, which comprise the remaining ¼ of the tooth. There are three long cusps at mesocone, endocone, and ectocone positions with two or more alternating larger and smaller cusps intercalated between them. There are roughly 105 teeth per row (*N* = 5; Table 2).

##### Distribution and ecology.

*Auriculellaminuta* is endemic to Oahu’s Koolau Mountain Range (Fig. 1B), found predominantly in the southern portion of the range with a few historical records from the southern edge of the northern Koolau Mountains. No elevational range information is available with these historical specimen records. The species is arboreal and found on vegetation, including *Cordylinefruticosa*, *Dioscoreaalata*, *Freycinetiaarborea*, *Kaduaaffinis*, *Lobelia* sp., *Psidiumguajava*, and *Touchardialatifolia*. Live specimens recorded in the BPBM collection were last collected by Olaf Oswald in Waiahole in 1931 and is considered herein extinct.

##### Remarks.

**Figure 6.**
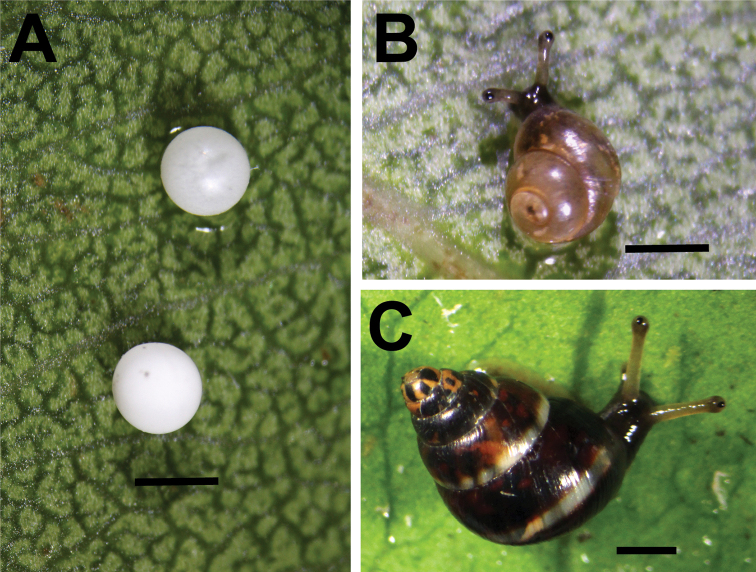
Photographs of live animals of *Auriculellagagneorum* sp. nov. **A** eggs **B** 1-day old juveniles **C** adult. Scale bars: 1 mm.

A holotype was not designated in the original description and the type series came from two different localities: Nuuanu collected by Cooke, and Palolo collected by both Cooke and Lyman (Pilsbry and Cooke 1915: 90). Five figures were provided with the original description (Pilsbry and Cooke 1915: pl. 25, figs 5–9) from Nuuanu, which according to the figure caption were based on specimens from BPBM and ANSP. The figure caption did not indicate which museum lots the figured specimens came from but the BPBM ledger in Cooke’s handwriting lists: BPBM 42377 “holotype”, figs 5, 9 (see note for ANSP 91816 below); BPBM 42378, “cotype”, fig. 8 (not ANSP 113294 as stated in Severns, 2011: 210); BPBM 42379 “paratypes”; BPBM 42380, “cotype”, fig. 7; BPBM 42381, “paracotypes”; 42382, “cotype”, fig. 6; BPBM 42383, “paracotypes”. The BPBM ledger documents that BPBM lots were the source of other type material: BPBM 42379 – 83 were the source for SMF 7127 (Zilch 1962: 78) and BPBM 42379, split from BPBM 13034, was the source lot for MCZ 73037. The ledger also indicated that two specimens were given to Dautzenberg whose collections were obtained by RBINS. Two ANSP lots 91816 and 113294 were received by Pilsbry from Cooke. The original label for ANSP 91816 is marked “cotype” and the source for fig. 9 in the description. Because the caption for figs 5–9 states that at least one of the figured specimens is from ANSP we believe this to be the source for fig. 9 rather than BPBM 42377 as stated in the BPBM ledger, although we do believe BPBM 42377 is the source for fig. 5. Johnson (1996) lists lot BPBM 42377 as the holotype citing the original BPBM specimen labelling. However, the species description is clearly based on multiple specimens all of which should be considered syntypes. In addition to the specimens from Nuuanu, the material from Palolo collected by both Lyman and Cooke are also part of the type series. There is only one lot of *Auriculellaminuta* (BPBM 12808) collected by Lyman from Palolo and although it is not labelled as being part of the type series it is likely the lot collected by Lyman that was mentioned in the species description. A second lot, BPBM 16435, lacks information on the collector but may be the lot collected by Cooke. We here designate lot BPBM 42377 as the lectotype, restricting the type locality to Nuuanu.

Unlike the other species traditionally placed in the *perpusilla* group, the shell of *A.minuta* is dextral rather than sinistral. The columella does not bear an axially oriented ridge like the one found in *A.perversa*. The palatal lamella is smooth and not undulate unlike that of *A.gagneorum* sp. nov. The epiphallus is short and well defined similar to *A.gagneorum* sp. nov., but unlike the long epiphallus of *A.perpusilla* or the poorly defined epiphallus of *A.perversa*. The appendix narrows abruptly at approximately ⅓ its length unlike the gently tapered appendix of *A.gagneorum* sp. nov.

#### Auriculella perpusilla

Taxon classificationAnimalia

E. Smith, 1873

7852DC76-4947-5E10-8509-62C5EF8381AA

Figures 1C , 3E, F , 4C , 5C

Auriculella perpusilla E. Smith in Gulick & Smith, 1873: 87, pl. 10, fig. 26; Pilsbry and Cooke 1915: 91–92, pl. 25, figs 1, 2; Cowie et al. 1995: 77; Johnson 1996: 193; Severns 2011: 210, pl. 82, fig. 3.

##### Type material.

***Holotype***: USA • 1; shell crushed; H = 4 mm, W = 2 ⅔ mm (according to original description); Honolulu County, Oahu, Koolau Mountains; 1918; John T. Gulick leg.; MCZ 39912.

##### Type locality.

“Kohalu” (*sic*, Kahaluu) on Oahu.

##### Diagnosis.

***Shell*.** Shell sinistral with inflated whorls, H = 4.4 ± 0.26 mm, W = 3.0 ± 0.15 mm, WH = 5.0 ± 0.14, AH = 2.1 ± 0.14 mm, AW = 1.5 ± 0.11 mm (*N* = 50; Table 2f). Columella in juveniles with a strong lamella that is reduced and covered by a thickening of the inner edge of the lip in adults. Some adults show a short projection or angulation where the columellar lamella was located. Parietal lamella is smooth and not undulate, extending 0.3 whorls into the aperture, and sometimes bears a weak angulation at mid-point. Shell color is pale tan or dark brown, with or without a single peripheral color band of pale tan or dark brown (Fig. 3F).

***Reproductive system*.** Phallus retractor muscle relatively short, attached apically to a long epiphallus, which is nearly ⅓ the length of the phallus (Fig. 4C). Appendix is nearly equal in length to the phallus. Appendix slightly over half the diameter of the phallus at its attachment, narrowing abruptly at ⅓ its length and remaining narrow to its terminus. Apical ⅔ of the phallus is broad, basal ⅓ narrows abruptly and remains narrow to the junction with the short atrium. Vagina is long and nearly half the length of the phallus.

***Radula*.**Radula with an irregular rachidian flanked on either side by rastriform marginal teeth, as diagnostic of the family (Fig. 5C). Each tooth has a long narrow base that expands slowly for ¾ of the length of the tooth before reaching the forward curving cusps, which comprise the remaining ¼ of the tooth. There are three long cusps at mesocone, endocone, and ectocone positions with two or more alternating larger and smaller cusps intercalated between them. There are roughly 127 teeth per row (*N* = 5; Table 2).

##### Distribution and ecology.

*Auriculellaperpusilla* is endemic to Oahu’s Koolau Mountain Range (Fig. 1C), recorded from across the range at elevations of 61 m to 1066 m. The species is arboreal and found on vegetation, including: *Antidesmapulvinatum*, *Cordylinefruticosa*, *Freycinetiaarborea*, *Kaduaaffinis*, *Lobelia* sp., *Metrosiderospolymorpha*, *Myrsine* sp., *Psidiumguajava*, *Psychotriakaduana*, *Syzygiumsandwicense*, *Touchardialatifolia*, and on unspecified ferns, tree trunks, and dead leaves. Recent observations are restricted to Tantalus (southern Koolau Mountains; Fig. 1C).

##### Remarks.

No holotype was designated in the original description which included a single figure and provided a single set of measurements: height 4 mm width 2 ⅔ mm. The shell donated by Gulick is MCZ 39912 and is labeled holotype. Pilsbry and Cooke (1915: 91) indicated that only a single shell existed; “The single specimen collected by Mr. Gulick and described by Mr. Smith, is unfortunately broken.” Consequently, MCZ 39912 is the holotype by monotypy.

Unlike *A.minuta*, *A.perpusilla* is sinistral and the columella does not bear an axially oriented ridge like the one found in *A.perversa*. The palatal lamella is smooth and not undulate like *A.gagneorum* sp. nov. The epiphallus is long unlike the poorly defined epiphallus of *A.perversa* or the short but well-defined epiphallus of *A.minuta* and *A.gagneorum* sp. nov. The appendix narrows abruptly at approximately ⅓ its length unlike *A.gagneorum* sp. nov.

#### Auriculella perversa

Taxon classificationAnimalia

Cooke, 1915

53F0C6DD-D2D5-564F-8C5C-2AD6F76944E1

Figures 1D , 3G, H , 4D , 5D

Auriculella perversa Cooke in Pilsbry & Cooke, 1915: 90–91, pl. 25, figs 3, 4; Cowie et al. 1995; 77; Johnson 1996: 193; Severns 2011: 210, pl. 82, fig. 2.

##### Type material.

***Lectotype***: USA • 1; H = 4.7 mm, W = 3.3 mm, AH = 2.2 mm, AW = 2.0 mm, WH = 5.1; Honolulu County, Oahu, Koolau Mountains, Nuuanu; Ridge 9, east side, on *Passiflorafoetida*; Cooke leg.; BPBM 42384, here designated.

***Paralectotypes***: USA • 1; Honolulu County, Oahu, Koolau Mountains, Nuuanu; Ridge 9, east side, on *Passiflorafoetida*; Cooke leg.; BPBM 42385.

***Paralectotypes not examined***: ANSP 91817 (6 spm), ANSP 108272 (13 spm), ANSP 163399 (1 spm), ANSP 163411 (5 spm), MCZ 73044 (2 spm), SMF 7090 (1 spm).

##### Type locality.

Oahu: Nuuanu. See

##### Remarks.

##### Diagnosis.

***Shell*.** Shell sinistral with inflated whorls, H = 4.4 ± 0.26 mm, W = 3.0 ± 0.23 mm, WH = 5.2 ± 0.08, AH = 2.0 ± 0.18 mm, AW = 1.4 ± 0.08 mm (Table 2). Columella in juveniles with a strong lamella that is reduced and covered by a thickening of the inner edge of the lip in adults. The columellar thickening usually bears an axially oriented ridge. Adults do not show a short projection or angulation where the columellar lamella was located. Parietal lamella is smooth and not undulate, extending 0.3 to 0.5 whorls into the aperture. Shell color is solid brown to dark brown with darker brown axial bands (Fig. 3H).

***Reproductive system*.** Phallus retractor muscle relatively short attached apically to a short and poorly defined epiphallus (Fig. 4D). Appendix is as long as the phallus and a bit over half the diameter of the phallus at its attachment, narrowing abruptly at ⅓ its length and remaining narrow to its terminus. Phallus is broad, narrowing only slightly at the junction with the short atrium. Vagina is short.

***Radula*.**Radula with an irregular rachidian flanked on either side by rastriform marginal teeth, as diagnostic of the family (Fig. 5D). Each tooth has a long narrow base that expands slowly for ¾ of the length of the tooth before reaching the forward curving cusps, which comprise the remaining ¼ of the tooth. There are three long cusps at mesocone, endocone, and ectocone positions with two or more alternating larger and smaller cusps intercalated between them. There are roughly 127 teeth per row (*N* = 4; Table 2).

##### Distribution and ecology.

*Auriculellaperversa* is endemic to Oahu’s southern Koolau Mountain Range (Fig. 1D), recorded from 61 m to 914 m elevation. *Auriculellaperversa* is arboreal and found on *Clermontia* sp., *Cordylinefruticosa*, *Dubautialaxa*, *Freycinetiaarborea*, *Metrosiderospolymorpha*, *Musa* sp., *Pritchardia* sp., *Psidiumguajava*, and unspecified ferns, tree trunks, and dead leaves. Prior to our recent surveys the last live specimens were collected in 1939 by O.H. Emerson, E.H. Bryan Jr., and D. Anderson on Kulepeamoa Ridge in the southern Koolau Mountain Range, and the only known extant population recorded occurs in Tantalus.

##### Remarks.

A holotype was not designated in the original description and the type series came from two different localities: Nuuanu collected by Cooke, and Kuliouou collected by Thaanum. Two figures were provided with the original description (Pilsbry and Cooke 1915: pl. 25, figs 3, 4) for material from Nuuanu at BPBM. However, the figure caption does not indicate type status or lot numbers. The BPBM ledger in Cooke’s handwriting lists: BPBM 42384 “holotype”, figs 3, 4; BPBM 42385, “paratypes”. The BPBM ledger documents that BPBM 42385 was also the source of MCZ 7034 and SMF 7090 (Zilch, 1962: 78). The ANSP online catalog list additional specimens from BPBM and labeled as syntypes: ANSP 163411, 91817, Nuuanu; ANSP 163399 Kuliousu [*sic*]. ANSP 108272 Kuliouou was collected by D. Thaanum. Johnson (1996: 193) stated that the “holotype” was BPBM 42384 based on its specimen label. However, it is clear that the original description was based on multiple specimens which should be considered syntypes. We here designate BPBM 42384 as the lectotype. As a result of this lectotype designation the type locality is restricted to Nuuanu.

Unlike *A.minuta*, the shell of *A.perversa* is sinistral. The columella bears an axially oriented ridge unlike all other species in the *perpusilla* group. The palatal lamella is smooth and not undulate like *A.gagneorum* sp. nov. The reproductive system includes a short and poorly defined epiphallus and an appendix that narrows abruptly at approximately ⅓ its length. The epiphallus is short and poorly defined unlike the long epiphallus of *A.perpusilla* or the short but well-defined epiphallus of *A.minuta* and *A.gagneorum* sp. nov.

#### Auriculella tenella

Taxon classificationAnimalia

Ancey, 1889

2AE53901-B5F9-5942-B966-5F7D8AB251FD

Figures 1E , 3I, J , 4E , 5E

Auriculella tenella Ancey, 1889: 232–233; Pilsbry and Cooke 1915: 99–100, pl. 19, figs 7, 8; Cowie et al. 1995:77; Wood and Gallichan 2008: 88, pl. 2, fig. 8, ix; Severns 2011: 204, pl. 79, fig. 5.

##### Type material.

***Lectotype***: USA • 1; H = 6.2 mm, W = 3.5 mm, AH = 2.3 mm, AW = 1.6 mm, WH = 6.6 whorls; Honolulu County, Oahu, Waianae Mountains; Baldwin leg.; BPBM 18943, here designated.

***Paralectotypes***: USA • 2; Honolulu County, Oahu, Waianae Mountains; Baldwin leg.; BPBM 285811.

***Paralectotypes not examined***: NMW 1955.158.24126 (1 spm); RBINS 10591 (accession, 1 spm).

##### Type locality.

“Waianae, dans la partie occidentale de l’île d’Oahu.” [Waianae, western part of Oahu Island].

##### Diagnosis.

***Shell*.** Shell sinistral with inflated whorls, H = 5.6 ± 0.8 mm, W = 3.0 ± 0.4 mm, WH = 6.5 ± 0.3, AH = 2.0 ± 0.3 mm, AW = 1.9 ± 0.3 mm (*N* = 50; Table 2). Columella in juveniles with two lamellae that are reduced and visible only deep within the aperture of adults. Columellar reflection lacks an axially oriented ridge. Parietal lamella is smooth and not undulate, extending 0.3 to 0.5 whorls into the aperture. Shell color straw to brown, indistinctly streaked with red, with or without a single darker brown marginal spiral band.

***Reproductive system*.** Phallus retractor muscle relatively long attached apically to a short but well-defined epiphallus (Fig. 4E). Appendix ⅓ longer and about half the diameter of the phallus at its attachment, narrowing abruptly at ⅓ its length and remaining narrow to its terminus. Phallus is broad, narrowing by half at the junction with the short atrium. Vagina is of moderate length.

***Radula*.**Radula with an irregular rachidian flanked on either side by rastriform marginal teeth, as diagnostic of the family (Fig. 5E). Each tooth has a long narrow base that expands slowly for ¾ of the length of the tooth before reaching the forward curving cusps, which comprise the remaining ¼ of the tooth. There are three long cusps at mesocone, endocone, and ectocone positions with two or more alternating larger and smaller cusps intercalated between them. There are roughly 129 teeth per row (*N* = 3; Table 2).

##### Distribution and ecology.

*Auriculellatenella* is endemic to Oahu’s Waianae Mountains, historically found throughout the range between 518 and 1227 m in elevation (Fig. 1E). This species is arboreal and found on *Broussaisia* sp., *Cordyline* sp., *Freycinetiaarborea*, *Lantana* sp., *Pelea* sp., *Sadleriacyatheoides*, *Bidens* sp., *Coprosma* sp., *Euphorbia* sp., *Metrosideros* sp., *Psychotria* sp., *Ilex* sp., *Philodendron* sp., and unspecified ferns, grasses, tree trunks, and small plants on stream banks. Occasionally, this species has been recorded on the ground on stones, dead leaves, and bark. The last live specimens in the BPBM collection were recorded in 1948. Our recent surveys documented the species in only three locations in the southern Waianae range.

##### Remarks.

A holotype was not designated in the original description, however, the type locality is listed as “Waianae” and collected by Baldwin. Ancey provided measurements in the original description, “Long., 6; diam., 3; alt. ap., 2 2/3 millim.”, which agree well with the designated lectotype. The ledger entry for BPBM 18943 lists four “types” collected by Baldwin from Waialae [*sic*]. However, only three specimens were found. The material probably came from Paul Geret who acquired Ancey’s collection after his death and subsequently sold it. Much of Ancey’s Hawaiian land and freshwater material was purchased by BPBM in 1908 (Johnson, 1996) but some was sold to other buyers. Both NMW 1955.158.24126 and RBINS 10591 (accession number) have Geret “cotype” labels (Wood and Gallichan 2008:88). Tomlin, the source of the NMW lot, had a sales list confirming purchase from the Ancey collection.

The shell of *A.tenella* has approximately seven nearly flat-sided whorls unlike *A.auricula*, *A.minuta*, *A.perpusilla* and *A.perversa*, which have approximately five whorls, and are inflated in all but *A.auricula*. *Auriculellatenella* is sinistral unlike *A.minuta* and does not bear an axially oriented columellar ridge like *A.perversa* or an undulating palatal lamella like *A.gagneorum* sp. nov. The epiphallus is short and well defined unlike the long epiphallus of *A.perpusilla* or the poorly defined epiphallus of *A.perversa*. The appendix narrows abruptly at approximately ⅓ its length unlike *A.gagneorum* sp. nov.

#### Auriculella gagneorum sp. nov.

Taxon classificationAnimalia

30A8D17A-D829-5DBA-AA22-55D2BD2C5CAC

http://zoobank.org/25f68bf8-12f1-461e-be17-263982427bb0

Figures 1F , 3K, L , 4F , 5F , 6A-C

##### Material examined.

***Holotype***: USA • 1, H = 4.7 mm, W = 3.4 mm, AH = 2.3 mm, AW = 1.8 mm, WH = 5.3 whorls; Honolulu County, Oahu, Waianae Mountains, Palawai Gulch; 710 m; 9 Feb. 2018; K. A. Hayes, N. W. Yeung, J. Slapcinsky; hand collected on *Pisoniaumbellifera*; GenBank: MT519824-MT519826, MT519866-MT519868, MT519889-MT519592; BPBM 285843.

***Paratypes***: USA – Honolulu County, Oahu, Waianae Mountains • 1; Puu Hapapa; 23 Jan 2013; D.T.A. Gary, K. Leung, D. R. Sischo, V. J. Costello; BPBM 285794 • 8; Puu Hapapa; 23 Jan 2013; D.T.A. Gary, K. Leung, D. R. Sischo, V. J. Costello; BPBM 285795 • 1; Palawai; 24 Dec 2014; D. R. Sischo and SEPP crew; BPBM 285799 • 3; Puu Hapapa; 24 Jan 2013; D.T.A. Gary, K. Leung, D. R. Sischo, V. J. Costello; BPBM 285796 • 2; Ekahanui; 17 Feb 2013; D.T.A. Gary, K. Leung, D. T. B. Ressler, V. J. Costello; BPBM 285797 • 1; Palawai; 24 Dec 2014; D. R. Sischo and SEPP crew; BPBM 285798 • 2; Palawai; 24 Dec 2014; D. R. Sischo and SEPP crew; BPBM 285800.

***Other material***: USA – Honolulu County, Oahu, Waianae Mountains • 37; Palikea Ridge; 12 October 1912; R. von Holt, Cooke; BPBM 24989 • 44; Palikea Ridge; 12 October 1912; von Holt, Cooke; BPBM 33011 • 27; Palikea Ridge; 12 October 1912; von Holt, Cooke; BPBM 33018 • 10; Palikea Ridge; 12 October 1912; von Holt, Cooke; BPBM 33006 • 3; Makua; 16 November 1913; Spalding; BPBM 34847 • 3; Palikea Ridge; 27 December 1914; Alice T. Cooke, C.M. Cooke; BPBM 38031 • 79; Palikea Ridge; 24 August 1922; R. von Holt, C.M. Cooke Jr., M.C. Neal; BPBM 59612 • 11; Napepeiauolelo; 25 March 1934; Meinecke, William H.; BPBM 127221 • 1; Palawai Gulch; 30 August 1935; D’Alte A. Welch, Glen W. Russ; BPBM 174037 • 15; Palawai Gulch; 30 August 1935; Glen W. Russ, D’Alte A. Welch; BPBM 174233 • 2; Palawai Gulch; 30 August 1935; D’Alte A. Welch, Glen W. Russ; BPBM 174141 • 3; Palawai Gulch; 30 August 1935; D’Alte A. Welch, Glen W. Russ; BPBM 174081 • 1; Manuwaikaalae Gulch; 28 March 1936; J. Winne, D’Alte A. Welch; BPBM 176456 • 2; Pohakea Gulch; 30 March 1936; J. Winne, D’Alte A. Welch; BPBM 176596 • 3; Pualii Gulch; 30 March 1936; J. Winne, D’Alte A. Welch; BPBM 176651 • 21; Pualii Gulch; 30 March 1936; J. Winne, D’Alte A. Welch; BPBM 176766 • 1; Kaaikukai Gulch; 03 April 1936; B. Bowen, D’Alte A. Welch; BPBM 176916 • 11; Kaaikukai; 03 April 1936; B. Bowen, D’Alte A. Welch; BPBM 176973 • 15; Palawai Gulch; 19 April 1936; J. Winne, D’Alte A. Welch; BPBM 177217 • 9; Palawai Gulch; 19 April 1936; J. Winne, D’Alte A. Welch; BPBM 177278 • 1; Kaaikukai Gulch; 05 May 1936; R. Yamaguchi, D’Alte A. Welch; BPBM 177468 • 1; Mount Kaala; 27 March 1937; F. Raymond Fosberg; BPBM 162712 • 11; Napepeiauolelo; 03 April 1938; William H. Meinecke, E. Meadows, Donald Anderson; BPBM 173979 • 9; Napepeiauolelo; 03 April 1938; William H. Meinecke, E. Meadows, Donald Anderson; BPBM 173980 • 2; Pualii Gulch; 03 April 1938; William H. Meinecke, E. Meadows,, Donald Anderson; BPBM 184885 • 5; Ekahanui Gulch; 16 September 1941; Rokuro Yamaguchi, Yoshio Kondo; BPBM 211563 • 2; Ekahanui Gulch; 16 September 1941; Rokuro Yamaguchi, Yoshio Kondo; BPBM 211678 • 6; Ekahanui Gulch; 16 September 1941; Rokuro Yamaguchi, Yoshio Kondo; BPBM 211723 • 5; Napepeiauolelo-Pualii Ridge; 15 October 1960; Yoshio Kondo, T.M. {T. Maa?}, George F. Arnemann, P.C. {Peter Char?}; BPBM 216123 • 2; Palawai Gulch; BPBM 183862 • 135; Palikea Ridge; R. von Holt, Cooke; BPBM 21823 • 17; Palikea Ridge; R. von Holt, Cooke; BPBM 21824 • 74; Palikea Ridge; Spalding; BPBM 22739 • 6; Palikea Ridge; Spalding; BPBM 19891 • 1; Palikea Ridge; Cooke; BPBM 16884.

##### Type locality.

Palawai Gulch, Waianae Mountains, Honolulu County, Oahu

##### Diagnosis.

***Shell*.** Shell sinistral with inflated whorls, H = 4.8 ± 0.3 mm, W = 3.2 ± 0.2 mm, WH = 5.4 ± 0.4, AH = 2.3 ± 0.1 mm, AW = 1.7 ± 0.1 mm (Table 2). Columella in juveniles with a strong lamella that is reduced and covered by a thickening of the inner edge of the lip in adults. Adults do not show a short projection or angular edge where the columellar lamella was located. Parietal lamella is often undulate, usually with three peaks, extending 0.2 to 0.5 whorls into the aperture. Shell color is white, pale tan or dark brown, with or without irregularly placed axial bands of brown, or with a single peripheral band of pale tan or dark brown. Specimens occasionally pale tan with two poorly defined dark bands on either side of a pale tan peripheral band.

***Reproductive system*.** Phallus retractor muscle long, attached apically to a short but well-defined epiphallus (Fig. 4F). Appendix slightly longer than the phallus. Appendix ⅔ the diameter of the phallus at its attachment, tapering gently to ⅓ its length, then remaining narrow to its terminus. Apical ¾ of the phallus is broad, tapering slightly both apically and basally, basal ¼ narrows slightly above junction with the short atrium. Vagina is short.

***Radula*.**Radula with an irregular rachidian flanked on either side by rastriform marginal teeth, as diagnostic of the family (Fig. 5F). Each tooth has a long narrow base that expands slowly for ¾ of the length of the tooth before reaching the forward curving cusps, which comprise the remaining ¼ of the tooth. There are three long cusps at mesocone, endocone, and ectocone positions with two or more alternating larger and smaller cusps intercalated between them. Number of teeth per row range from 135 to 153 (*N* = 3; Table 2).

##### Distribution and ecology.

*Auriculellagagneorum* sp. nov. is endemic to Oahu’s Waianae Mountain Range and was recorded as a potentially new species primarily from the southern Waianae Mountain Range, with several populations in the northern part of the range (Fig. 1F). The species is arboreal and has been found on *Antidesmaplatyphyllum*, *Broussaisiaarguta*, *Lantana* sp., *Melicopeanisate*, *Myrsinelessertiana*, and occasionally on unspecified ferns and dead leaves. The last known record of this species prior to recent surveys was by Yoshio Kondo, T. Maa, George F. Arnemann, and Peter Char in 1960. From 2013 to 2018 we recorded extant populations of this species from three locations in the southern Waianae Mountains.

##### Remarks.

The shell is sinistral unlike *A.minuta* and the columella does not bear an axially oriented ridge like the one found in *A.perversa*. The palatal lamella is often undulate unlike all other members of the *A.perpusilla* group. The epiphallus is short but well defined similar to *A.minuta* but unlike the long epiphallus of *A.perpusilla* or the poorly defined epiphallus of *A.perversa*. The appendix tapers gently unlike the appendices of *A.auricula*, *A.minuta*, *A.perpusilla*, *A.perversa*, and *A.tenella* which all narrow abruptly.

##### Etymology.

Named in honor of Betsy and Wayne Gagne for their indefatigable efforts advocating for the conservation of Hawaii’s unique and highly endangered biota.

## Discussion

The *Auriculellaperpusilla* species group (*A.perpusilla*, *A.perversa*, *A.minuta*) was defined as having species with small, thin, relatively low spired shells of approximately five inflated whorls. *Auriculellagagneorum* sp. nov., shares these shell characteristics. These four species can be distinguished from one another using a suite of morphological features including shell chirality (only *A.minuta* is dextral); presence of axially oriented ridge of the columella (only present in *A.perversa*); appearance of the palatal lamella (undulated only in *Auriculellagagneorum* sp. nov.); length of the epiphallus (those of both *Auriculellagagneorum* sp. nov. and *minuta* are short and well-defined); and development of the appendix (tapers gently in *Auriculellagagneorum* sp. nov. and narrow abruptly in others). The DNA data corroborate the difference seen in anatomy and conchology. In contrast to expectations based on shell morphology alone, the *perpusilla* group is not monophyletic and *Auriculellagagneorum* sp. nov. is not closely related to either *A.perpusilla* or *A.perversa*, the only other extant members of the group for which DNA data are available (Fig. 2). Instead, *A.gagneorum* sp. nov. clusters with *A.tenella*, a high spired and tightly coiled species from the *castanea* group, which also occurs in the Waianae Mountains. Similarly, *A.perpusilla* and *A.perversa* are more closely related to species with highly dissimilar shell morphologies, *A.ambusta* and *A.montana*, respectively (Fig. 2). The latter two have large, thick shells and are usually placed in the *auricula* group with other robust species. Patterns of relatedness recovered in our phylogenetic analyses indicate these gross shell characters, which are unlikely to be independent of one another, are insufficient for delineating taxa or characterizing relationships within the genus. Multiple instances of convergence in shell morphology across the genus may be explained by adaptation to similar microhabitats, or non-adaptive diversification combined with constraints on shell morphospace (Gittenberger 1991; Cowie 1995; Rundell and Price 2009; Chiba and Cowie 2016; Gillespie et al. 2018). Disentangling the processes responsible for these patterns will require additional studies of the functional morphology, ecology, and behavior of *Auriculella* species.

Historically, all four species treated here once had much larger geographic ranges, with multiple populations recorded in the last century (Fig. 1). Like nearly all land snail species across Hawaii, *Auriculella* spp. numbers have declined dramatically with an estimated 45% of the species considered extinct, and many historical populations extirpated as a result of habitat destruction, invasive species, and possibly climate change. Despite the grim statistics, there remain a number of species that can yet be saved from extinction, but only with a clear understanding of their systematics, biogeography and ecology. For example, *A.tenella*, *A.gagneorum* sp. nov., *A.perversa*, and *A.perpusilla*, are now known from only three locations for each of these species. These data combined with knowledge of reproduction and population growth rates can be used to better manage these imperiled species.

Low reproductive and growth rates are often characteristic of species that have evolved on isolated oceanic islands (MacArthur and Wilson 2001; Covas 2011), and *Auriculella* spp. are probably no exception. Two laboratory reared adults of *Auriculellagagneorum* sp. nov. produced 33 eggs in 250 days between 17 May 2018 and 23 January 2019 (Fig. 6A–C). The delicate nature of the eggs of this imperiled species permitted the measurement of only three eggs, which had an average diameter of 0.99 ± 0.05 mm. These large eggs, relative to the size of the animal, take approximately 58 days to hatch (Lindsay Renshaw, pers comm.). Such low fecundity in combination with extreme range reduction decreases the chances of long-term species and population persistence (Bick et al. 2018), particularly in the face of predation by introduced predators (Chiba and Cowie 2016).

Updated and comprehensive assessments of the systematics, biogeography, and ecology of taxa are necessary for effective management and development of long-term recovery plans. Additional surveys to locate remaining species and persisting populations are needed now, while there is still an opportunity to prevent or slow the rate of species loss (Solem 1990; Yeung and Hayes 2018). These surveys provide important opportunities to study and preserve species and develop populations for captive rearing, which in turn can be repatriated to protective enclosures in natural habitats with the goal of ultimately reintroducing species back into the wild (Natural Area Reserves Program 2016; Yeung and Hayes 2018). Our surveys have recovered species not recorded alive since the 1950s (e.g., *Auriculellaperpusilla*, *A.perversa*, *A.tenella*) and others feared extinct (Yeung et al. 2015, 2018). They have also uncovered several previously undescribed species, indicating that there is still much to learn about this highly imperiled fauna, and still hope that we might save some of it for future generations (Solem 1990).

## Supplementary Material

XML Treatment for Auriculella

XML Treatment for Auriculella auricula

XML Treatment for Auriculella minuta

XML Treatment for Auriculella perpusilla

XML Treatment for Auriculella perversa

XML Treatment for Auriculella tenella

XML Treatment for Auriculella gagneorum

## Supplementary materials

Supplementary material 1 Non-type material examined for *Auriculellaauricula*, *A.minuta*, *A.perpusilla*, *A.perversa*, and *A.tenella* Data type species data Explanation note All material examined for *A.gagneorum* sp. nov. is provided in the body of the manuscript. https://binary.pensoft.net/file/432898This dataset is made available under the Open Database License (http://opendatacommons.org/licenses/odbl/1.0/). The Open Database License (ODbL) is a license agreement intended to allow users to freely share, modify, and use this Dataset while maintaining this same freedom for others, provided that the original source and author(s) are credited.Norine W. Yeung, John Slapcinsky, Ellen E. Strong, Jaynee R. Kim, Kenneth A. Hayes
