# Supplementary material for: Overlooked but not forgotten: the first new extant species of Hawaiian land snail described in 60 years, Auriculellagagneorum sp. nov. (Achatinellidae, Auriculellinae)
Source: Zookeys. 2020 Jul 20;950:1–31. doi: 10.3897/zookeys.950.50669 (PMC7387373; doi:10.3897/zookeys.950.50669)
Supplement: Supplementary material 1 — Non-type material examined for Auriculellaauricula, A.minuta, A.perpusilla, A.perversa, and A.tenella [file zookeys-950-001-s001.docx]

**Appendix 1: Non-type material examined for *Auriculella auricula*, *A. minuta, A. perpusilla, A. perversa,* and *A. tenella*. All material examined for *A. gagneorum* sp. nov. is provided in the body of the manuscript.**

*Auriculella auricula* (Férussac, 1821)

USA – Honolulu County, Oahu, Koolau Mountains • 116; Ahuimanu; Spalding leg.; BPBM 21572 • 49; Haiku; 03 Jan 1918; Meinecke, William H. leg.; BPBM 103163 • 65; Hillebrand Glen; 25 May 1903; Cooke leg.; BPBM 12653 • 76; Hillebrand Glen; 15 May 1915; Marcel Desnouee leg.; BPBM 39060 • 219; Hillebrand Glen; 15 May 1915; Emerson, J.S. leg.; BPBM 103111 • 168; Hillebrand Glen; Cooke leg.; BPBM 12647 • 74; Hillebrand Glen; Cooke leg.; BPBM 12738 • 91; Iolekaa Valley; 20 Nov 1932; Meinecke, William H. leg.; BPBM 122773 • 11; Iolekaa Valley; 20 Nov 1932; Meinecke, William H. leg.; BPBM 122774 • 11; Iolekaa Valley; 20 Nov 1932; Meinecke, William H. leg.; BPBM 122775 • 1; Iolekaa Valley; 20 Nov 1932; Meinecke, William H. leg.; BPBM 122777 • 154; Kaau Trail; 01 Jan 1931; Russ, Glen W. leg.; BPBM 133898 • 2; Kailua-Manoa; 22 Sept 1935; Usinger, Robert L. leg.; BPBM 134862 • 9; Kalihi; Gulick leg.; BPBM 71081 • 3; Kalihi-Kapalama; 13 Sept 1913; Emerson, J.S. leg.; BPBM 103112 • 17; Kalihi-Manaiki Ridge; 27 Mar 1939; W. Donalgaho, Donald Anderson, R. Yamaguchi leg.; BPBM 180817 • 1; Kalihi-Nuuanu; 31 Aug 1903; Cooke leg.; BPBM 12689 • 31; Kalihi-Nuuanu; 15 Jun 1940; W.J. Semmens leg.; BPBM 185330 • 1; Kalihi-Nuuanu; C.H., C.M. Cooke leg.; BPBM 19915 • 29; Kalihi-Nuuanu; C.H., C.M. Cooke leg.; BPBM 19921 • 8; Kaneohe; 22 Mar 1940; E. Meadows, Donald Anderson leg.; BPBM 184947 • 32; Kaneohe; 22 Mar 1940; E. Meadows, Donald Anderson leg.; BPBM 184966 • 1; Kaneohe; 22 Mar 1940; E. Meadows, Donald Anderson leg.; BPBM 184967 • 4; Kaneohe; 22 Mar 1940; E. Meadows, Donald Anderson leg.; BPBM 184968 • 25; Kaneohe; 23 Dec 1946; Gordon Harrison leg.; BPBM 210308 • 9; Kaneohe; 23 Dec 1946; Gordon Harrison leg.; BPBM 210309 • 13; Kaneohe; Spalding leg.; BPBM 17785 • 16; Kapalama; 20 Mar 1935; Yoshio Kondo, Donald Anderson leg.; BPBM 184098 • 29; Kapalama; Judd, Alfred F. leg.; BPBM 109302 • 46; Kauhalemoa Ridge; 20 Jul 1912; Emerson, J.S., Bryan, E.H. leg.; BPBM 103089 • 88; Kekio-Pukele Stream; Russ, Glen W. leg.; BPBM 133885 • 41; Konahuanui; 11 Apr 1920; M.C. Neal leg.; BPBM 58901 • 1; Konahuanui; 20 Mar 1928; Oswald, Olaf leg.; BPBM 90105 • 6; Konahuanui; 20 Mar 1928; Oswald, Olaf leg.; BPBM 90106 • 1; Konahuanui; 09 May 1943; Elwood C. Zimmerman leg.; BPBM 212045 • 2; Konahuanui; Thwing leg.; BPBM 25014 • 10; Konahuanui Trail; 01 Jun 1909; Forbes leg.; BPBM 19597 • 6; Konahuanui Trail; C.N. Forbes leg.; BPBM 169031 • 29; Konahuanui-Pauoa; 01 Mar 1920; D. Wesley Garber leg.; BPBM 58812 • 8; Konahuanui-Pauoa; 01 Mar 1920; D. Wesley Garber leg.; BPBM 58813 • 3; Koolau; 27 Feb 1915; Dimond, H. Waterhouse leg.; BPBM 74742 • 1; Koolau; 27 Feb 1915; Dimond, H. Waterhouse leg.; BPBM 74754 • 2; Koolau; 27 Feb 1915; Dimond, H. Waterhouse leg.; BPBM 74772 • 2; Koolau; 27 Feb 1915; Dimond, H. Waterhouse leg.; BPBM 74773 • 1; Koolau; 27 Feb 1915; Dimond, H. Waterhouse leg.; BPBM 74794 • 1; Koolau; 27 Feb 1915; Dimond, H. Waterhouse leg.; BPBM 74795 • 5; Koolau; 27 Feb 1915; Dimond, H. Waterhouse leg.; BPBM 74801 • 6; Koolau; 27 Feb 1915; Dimond, H. Waterhouse leg.; BPBM 74916 • 35; Koolau; 27 Feb 1915; Dimond, H. Waterhouse leg.; BPBM 74917 • 3; Koolau; 27 Feb 1915; Dimond, H. Waterhouse leg.; BPBM 74980 • 2; Koolau; Cooke leg.; BPBM 12839 • 3; Koolau; Baldwin leg.; BPBM 18920 • 1; Koolau; Thwing leg.; BPBM 25033 • 1; Koolau; BPBM 25038 • 3; Kuliouou; 01 Jan 1922; Wm. D. Wilder leg.; BPBM 51395 • 68; Luakaha; 05 May 1903; Cooke leg.; BPBM 12661 • 62; Luakaha; 05 May 1903; Cooke leg.; BPBM 12662 • 58; Luakaha; 05 May 1903; Cooke leg.; BPBM 12663 • 61; Luakaha; 05 May 1903; Cooke leg.; BPBM 12664 • 34; Luakaha; 05 May 1903; Cooke leg.; BPBM 12665 • 54; Luakaha; 05 May 1903; Cooke leg.; BPBM 12666 • 39; Luakaha; 05 May 1903; Cooke leg.; BPBM 12667 • 121; Luakaha; 05 May 1903; Cooke leg.; BPBM 12668 • 36; Luakaha; 05 May 1903; Cooke leg.; BPBM 12669 • 4; Luakaha; 05 May 1903; Cooke leg.; BPBM 12670 • 35; Luakaha; 08 May 1903; Cooke leg.; BPBM 12671 • 222; Luakaha; 26 Apr 1908; Cooke leg.; BPBM 16388 • 68; Luakaha; 31 May 1908; Cooke leg.; BPBM 16571 • 3; Luakaha; 16 Jun 1924; C.A., C.M. Cooke, Jr., Murphy leg.; BPBM 76782 • 3; Luakaha; 16 Jun 1924; C.A., C.M. Cooke, Jr., Murphy leg.; BPBM 76823 • 3; Luakaha; 16 Jun 1924; C.A., C.M. Cooke, Jr., Murphy leg.; BPBM 76824 • 58; Luakaha; 03 Aug 1933; Brown, Pilsbry, Anderson, Cooke (Malac. Staff) leg.; BPBM 119138 • 3; Luakaha; 03 Aug 1933; Brown, Pilsbry, Anderson, Cooke (Malac. Staff) leg.; BPBM 119139 • 8; Luakaha; 03 Aug 1933; Brown, Pilsbry, Anderson, Cooke (Malac. Staff) leg.; BPBM 119140 • 52; Luakaha; 03 Aug 1933; Brown, Pilsbry, Anderson, Cooke (Malac. Staff) leg.; BPBM 119141 • 5; Luakaha; 03 Aug 1933; Brown, Pilsbry, Anderson, Cooke (Malac. Staff) leg.; BPBM 119142 • 11; Luakaha; 03 Aug 1933; Brown, Pilsbry, Anderson, Cooke (Malac. Staff) leg.; BPBM 119143 • 96; Luakaha; 03 Aug 1933; Brown, Pilsbry, Anderson, Cooke (Malac. Staff) leg.; BPBM 119157 • 36; Luakaha; 03 Aug 1933; Brown, Pilsbry, Anderson, Cooke (Malac. Staff) leg.; BPBM 119158 • 50; Luakaha; 03 Aug 1933; Brown, Pilsbry, Anderson, Cooke (Malac. Staff) leg.; BPBM 119159 • 10; Luakaha; 03 Aug 1933; Brown, Pilsbry, Anderson, Cooke (Malac. Staff) leg.; BPBM 119160 • 1; Luakaha; Cooke leg.; BPBM 42406 • 84; Luakaha Falls; 05 May 1903; Cooke leg.; BPBM 12672 • 39; Luakaha Falls; 05 May 1903; Cooke leg.; BPBM 12673 • 26; Luakaha Trail Ridge; 03 Aug 1933; Brown, Pilsbry, Anderson, Cooke (Malac. Staff) leg.; BPBM 119172 • 23; Luakaha Trail Ridge; 03 Aug 1933; Brown, Pilsbry, Anderson, Cooke (Malac. Staff) leg.; BPBM 119173 • 6; Luakaha Trail Ridge; 03 Aug 1933; Brown, Pilsbry, Anderson, Cooke (Malac. Staff) leg.; BPBM 119174 • 1; Lulumahu; 07 Dec 1913; Cooke leg.; BPBM 35459 • 63; Makiki; 01 Jan 1922; Wm. D. Wilder leg.; BPBM 51414 • 5; Makiki; 12 Jan 1930; Meinecke, William H. leg.; BPBM 120660 • 52; Malamalama Valley; 30 Nov 1919; Horner, J., Bryan, E.H. leg.; BPBM 103106 • 8; Manoa; 25 Feb 1912; Spalding leg.; BPBM 23568 • 215; Manoa; 20 May 1913; J.S. Emerson leg.; BPBM 34067 • 3; Manoa; 20 May 1913; J.S. Emerson leg.; BPBM 34073 • 2; Manoa; 10 Jun 1913; J.S. Emerson leg.; BPBM 34765 • 21; Manoa; 10 Jun 1913; J.S. Emerson leg.; BPBM 34768 • 1; Manoa; 10 Jun 1913; J.S. Emerson leg.; BPBM 34769 • 2; Manoa; 19 Jul 1913; J.S. Emerson leg.; BPBM 34776 • 19; Manoa; 28 Aug 1929; Meinecke, William H. leg.; BPBM 94138 • 36; Manoa; 28 Aug 1929; Meinecke, William H. leg.; BPBM 94139 • 3; Manoa; 28 Aug 1929; Meinecke, William H. leg.; BPBM 94140 • 17; Manoa; 20 Apr 1932; Noel L.H. Krauss leg.; BPBM 211264 • 2; Manoa; 16 Sept 1933; Noel L.H. Krauss leg.; BPBM 211256 • 16; Manoa; 14 Sept 1941; William H. Meinecke, Meadows, Malacology Staff leg.; BPBM 194643 • 36; Manoa; BPBM 56958 • 1; Manoa; BPBM 56959 • 63; Manoa; BPBM 56970 • 78; Manoa; BPBM 56971 • 92; Manoa; BPBM 56973 • 86; Manoa; BPBM 56975 • 26; Manoa; BPBM 56976 • 9; Manoa; Charles H. Merriam leg.; BPBM 191050 • 2; Manoa Cliff Trail; 15 Nov 1913; Meinecke, William H. leg.; BPBM 120615 • 8; Manoa Cliff Trail; 31 Mar 1914; J.S. Emerson leg.; BPBM 36216 • 9; Manoa Cliff Trail; 01 Jun 1933; Fosberg, Dunker, K., Anderson, D. leg.; BPBM 129479 • 2; Manoa Cliff Trail; 16 Jun 1933; Fosberg, Dunker, K., Anderson, D. leg.; BPBM 129490 • 4; Manoa Cliff Trail; 16 Nov 1933; Donald Anderson leg.; BPBM 184021 • 5; Manoa Cliff Trail; 16 Nov 1933; Donald Anderson leg.; BPBM 184022 • 13; Manoa Cliff Trail; 23 Jan 1937; M., Donald Anderson leg.; BPBM 164138 • 2; Manoa Cliff Trail; 23 Jan 1937; M., Donald Anderson leg.; BPBM 164139 • 5; Manoa Cliff Trail; 23 Jan 1937; M., Donald Anderson leg.; BPBM 164140 • 9; Manoa Cliff Trail; 23 Jan 1937; M., Donald Anderson leg.; BPBM 164141 • 1; Manoa Cliff Trail; 23 Jan 1937; M., Donald Anderson leg.; BPBM 164142 • 25; Manoa Cliff Trail; 23 Jan 1937; M., Donald Anderson leg.; BPBM 164143 • 23; Manoa-Palolo; 02 Aug 1912; Vasconcelles, M. leg.; BPBM 103094 • 79; Manoa-Palolo; 26 May 1914; Meinecke, William H. leg.; BPBM 120489 • 46; Manoa-Palolo; 18 Oct 1919; Bryan, E.H. leg.; BPBM 49045 • 28; Manoa-Palolo; 18 Oct 1919; Bryan, E.H. leg.; BPBM 49049 • 47; Manoa-Palolo; 24 Oct 1920; M.C. Neal leg.; BPBM 58887 • 37; Manoa-Palolo; 15 Mar 1925; William H. Meinecke leg.; BPBM 167833 • 6; Manoa-Palolo; 13 Mar 1927; Meinecke, William H. leg.; BPBM 93097 • 12; Manoa-Palolo; 08 Aug 1929; Russ, Glen W. leg.; BPBM 133917 • 11; Manoa-Palolo; 27 Dec 1929; Meinecke, William H. leg.; BPBM 120587 • 20; Manoa-Palolo; 01 Dec 1931; Russ, Glen W. leg.; BPBM 133916 • 1; Manoa-Palolo; 18 Mar 1932; Meinecke, Welch leg.; BPBM 112791 • 9; Manoa-Palolo; 19 Mar 1933; Anderson, Donald leg.; BPBM 129497 • 38; Manoa-Palolo; 01 Apr 1933; Meinecke, William H. leg.; BPBM 120599 • 15; Manoa-Palolo; 01 Apr 1933; Meinecke, William H. leg.; BPBM 120600 • 19; Manoa-Palolo; 19 May 1933; Anderson, Donald leg.; BPBM 129495 • 20; Manoa-Palolo; 10 Jan 1935; Yoshio Kondo, Donald Anderson leg.; BPBM 184072 • 7; Manoa-Palolo; 22 Sept 1935; Usinger, Robert L. leg.; BPBM 134857 • 6; Manoa-Palolo; 03 Nov 1935; Robert L. Usinger leg.; BPBM 184074 • 2; Manoa-Palolo; 17 Oct 1940; T. Abbott, Donald Anderson leg.; BPBM 189020 • 4; Manoa-Palolo; 17 Oct 1940; T. Abbott, Donald Anderson leg.; BPBM 189021 • 14; Manoa-Palolo; 14 Sept 1941; William H. Meinecke, Meadows, Malacology Staff leg.; BPBM 194670 • 4; Manoa-Palolo; 14 Sept 1941; William H. Meinecke, Meadows, Malacology Staff leg.; BPBM 194671 • 121; Manoa-Palolo; 17 Oct 1941; T. Abbott, Donald Anderson leg.; BPBM 211960 • 2; Manoa-Palolo; J. Waterhouse leg.; BPBM 22333 • 51; Manoa-Palolo; Emerson, J.S., O.H.E. leg.; BPBM 103093 • 156; Manoa-Palolo; Emerson, J.S., Bryan, E.H. leg.; BPBM 103095 • 3; Manoa-Palolo; Judd, Alfred F. leg.; BPBM 109206 • 44; Mauna Kapu; 01 Dec 1909; Forbes leg.; BPBM 20177 • 91; Mount Independence; 22 Aug 1903; Cooke leg.; BPBM 12683 • 1; Mount Independence; Cooke leg.; BPBM 12793 • 1; Mount Independence; Cooke leg.; BPBM 42405 • 1; Mount Olympus; 14 Apr 1908; Cooke leg.; BPBM 16299 • 3; Mount Olympus; 12 May 1918; J.C. Bridwell leg.; BPBM 46355 • 2; Mount Olympus; 01 Jan 1922; Wm. D. Wilder leg.; BPBM 51405 • 2; Mount Olympus; 06 Oct 1935; Robert L. Usinger leg.; BPBM 184082 • 11; Mount Olympus; Thwing leg.; BPBM 25030 • 5; Niniko; 16 May 1923; Oswald, Olaf leg.; BPBM 54273 • 2; Niniko; 16 May 1923; Oswald, Olaf leg.; BPBM 54274 • 12; Nuuanu; 12 May 1903; Cooke leg.; BPBM 12674 • 68; Nuuanu; 12 May 1903; Cooke leg.; BPBM 12679 • 68; Nuuanu; 12 May 1903; Cooke leg.; BPBM 12680 • 57; Nuuanu; 12 May 1903; Cooke leg.; BPBM 12681 • 68; Nuuanu; 12 May 1903; Cooke leg.; BPBM 12682 • 15; Nuuanu; 24 May 1903; Cooke leg.; BPBM 12675 • 60; Nuuanu; 24 May 1903; Cooke leg.; BPBM 12676 • 64; Nuuanu; 24 May 1903; Cooke leg.; BPBM 12677 • 65; Nuuanu; 24 May 1903; Cooke leg.; BPBM 12678 • 65; Nuuanu; 25 May 1903; Cooke leg.; BPBM 12651 • 59; Nuuanu; 25 May 1903; Cooke leg.; BPBM 12652 • 27; Nuuanu; 25 May 1903; Cooke leg.; BPBM 12654 • 64; Nuuanu; 25 May 1903; Cooke leg.; BPBM 12655 • 52; Nuuanu; 25 May 1903; Cooke leg.; BPBM 12656 • 20; Nuuanu; 25 May 1903; Cooke leg.; BPBM 12657 • 21; Nuuanu; 25 May 1903; Cooke leg.; BPBM 12658 • 47; Nuuanu; 25 May 1903; Cooke leg.; BPBM 12659 • 1; Nuuanu; 25 May 1903; Cooke leg.; BPBM 12660 • 13; Nuuanu; 19 Jun 1903; Cooke leg.; BPBM 12716 • 54; Nuuanu; 22 Aug 1903; Cooke leg.; BPBM 12684 • 70; Nuuanu; 22 Aug 1903; Cooke leg.; BPBM 12685 • 68; Nuuanu; 31 Aug 1903; Cooke leg.; BPBM 12686 • 68; Nuuanu; 16 Nov 1904; Cooke leg.; BPBM 12745 • 5; Nuuanu; 28 Nov 1906; Cooke leg.; BPBM 15188 • 2; Nuuanu; 01 Mar 1907; Cooke leg.; BPBM 15306 • 6; Nuuanu; 01 Mar 1907; Cooke leg.; BPBM 15381 • 20; Nuuanu; 01 Mar 1907; R.A. Cooke leg.; BPBM 15454 • 7; Nuuanu; 30 Nov 1907; Bartsch, Cooke leg.; BPBM 15755 • 4; Nuuanu; 25 May 1908; Spalding leg.; BPBM 16564 • 3; Nuuanu; 31 May 1908; Cooke leg.; BPBM 16582 • 31; Nuuanu; 07 Jul 1909; Robinson, Cooke leg.; BPBM 19626 • 13; Nuuanu; 07 Jul 1909; Robinson, Cooke leg.; BPBM 19651 • 31; Nuuanu; 01 Dec 1909; C.N. Forbes leg.; BPBM 169033 • 49; Nuuanu; 07 Dec 1909; Forbes leg.; BPBM 20214 • 149; Nuuanu; 16 Sept 1911; Cooke leg.; BPBM 23127 • 10; Nuuanu; 28 Nov 1911; H.Hatch, A.J.Cooke, C.M.Cooke leg.; BPBM 23423 • 25; Nuuanu; 28 Nov 1911; H.Hatch, A.J.Cooke, C.M.Cooke leg.; BPBM 23424 • 23; Nuuanu; 04 Jan 1912; A. Butt, Cooke leg.; BPBM 23655 • 26; Nuuanu; 04 Jan 1912; A. Butt, Cooke leg.; BPBM 23659 • 2; Nuuanu; 04 Jan 1912; A. Butt, Cooke leg.; BPBM 23661 • 19; Nuuanu; 04 Jan 1912; A. Butt, Cooke leg.; BPBM 23671 • 69; Nuuanu; 26 Apr 1913; J.S. Emerson, J.M. Cox leg.; BPBM 34020 • 23; Nuuanu; 25 May 1913; C.M. Cooke Jr. III, Carolene, C.M. Cooke Jr. leg.; BPBM 35085 • 61; Nuuanu; 16 Dec 1913; Cooke leg.; BPBM 35465 • 5; Nuuanu; 28 Dec 1913; Cooke leg.; BPBM 35503 • 16; Nuuanu; 01 Jan 1914; H. Hatch, C.M. Cooke leg.; BPBM 35532 • 66; Nuuanu; 01 Jan 1914; H. Hatch, C.M. Cooke leg.; BPBM 35541 • 10; Nuuanu; 15 May 1915; J.S. Emerson leg.; BPBM 39054 • 1; Nuuanu; 30 Oct 1915; A. Gouveia leg.; BPBM 40465 • 16; Nuuanu; 30 Oct 1915; A. Gouveia leg.; BPBM 40476 • 20; Nuuanu; 30 Oct 1915; A. Gouveia leg.; BPBM 40477 • 4; Nuuanu; 30 Oct 1915; A. Gouveia leg.; BPBM 40478 • 4; Nuuanu; 30 May 1916; S. Malnix, Carolene, Charley, C.M.Cooke leg.; BPBM 41935 • 12; Nuuanu; 20 Jun 1917; J.C. Bridwell leg.; BPBM 43912 • 1; Nuuanu; 21 Jun 1917; C.M. Cooke leg.; BPBM 44352 • 187; Nuuanu; 01 Jan 1922; Wm. D. Wilder leg.; BPBM 51417 • 1; Nuuanu; 01 Jan 1922; Wm. D. Wilder leg.; BPBM 51418 • 1; Nuuanu; 01 Jan 1922; Wm. D. Wilder leg.; BPBM 51419 • 1; Nuuanu; 01 Jan 1922; Wm. D. Wilder leg.; BPBM 51421 • 1; Nuuanu; 21 Oct 1928; Peter H., M. Buck leg.; BPBM 180416 • 15; Nuuanu; 21 Oct 1928; Peter H., M. Buck leg.; BPBM 180417 • 2; Nuuanu; 21 Oct 1928; Peter H., M. Buck leg.; BPBM 180418 • 24; Nuuanu; 02 Jul 1930; McAllister leg.; BPBM 98947 • 42; Nuuanu; 02 Jul 1930; McAllister leg.; BPBM 98948 • 10; Nuuanu; 10 Oct 1932; Russ, Glen W. leg.; BPBM 134001 • 4; Nuuanu; 03 Aug 1933; Brown, Pilsbry, Anderson, Cooke (Malac. Staff) leg.; BPBM 119201 • 18; Nuuanu; 03 Aug 1933; Brown, Pilsbry, Anderson, Cooke (Malac. Staff) leg.; BPBM 119202 • 7; Nuuanu; 03 Aug 1933; Brown, Pilsbry, Anderson, Cooke (Malac. Staff) leg.; BPBM 119203 • 49; Nuuanu; 23 May 1935; C.M. Cooke, Jr., Others leg.; BPBM 161492 • 4; Nuuanu; 23 May 1935; C.M. Cooke, Jr., Others leg.; BPBM 161495 • 10; Nuuanu; 23 May 1935; C.M. Cooke, Jr., Others leg.; BPBM 161496 • 6; Nuuanu; 23 May 1935; C.M. Cooke, Jr., Others leg.; BPBM 161497 • 2; Nuuanu; 23 May 1935; C.M. Cooke, Jr., Others leg.; BPBM 161498 • 1; Nuuanu; 23 May 1935; C.M. Cooke, Jr., Others leg.; BPBM 161520 • 16; Nuuanu; 28 Feb 1943; William H. Meinecke leg.; BPBM 189868 • 22; Nuuanu; 18 Apr 1943; William H. Meinecke leg.; BPBM 189876 • 6; Nuuanu; 18 Apr 1943; William H. Meinecke leg.; BPBM 189879 • 5; Nuuanu; 21 Apr 1943; William H. Meinecke leg.; BPBM 189874 • 3; Nuuanu; BPBM 11023 • 3; Nuuanu; Cooke leg.; BPBM 12645 • 11; Nuuanu; Cooke leg.; BPBM 12646 • 41; Nuuanu; Cooke leg.; BPBM 12648 • 60; Nuuanu; Cooke leg.; BPBM 12649 • 73; Nuuanu; Cooke leg.; BPBM 12650 • 56; Nuuanu; BPBM 12681 • 24; Nuuanu; Cooke leg.; BPBM 12690 • 51; Nuuanu; Cooke leg.; BPBM 12739 • 62; Nuuanu; Cooke leg.; BPBM 12740 • 35; Nuuanu; Cooke leg.; BPBM 12741 • 42; Nuuanu; Cooke leg.; BPBM 12742 • 31; Nuuanu; Cooke leg.; BPBM 12743 • 16; Nuuanu; Cooke leg.; BPBM 12744 • 48; Nuuanu; Robinson, Cooke leg.; BPBM 19612 • 45; Nuuanu; Forbes leg.; BPBM 20443 • 8; Nuuanu; Forbes leg.; BPBM 20451 • 27; Nuuanu; Forbes leg.; BPBM 20452 • 32; Nuuanu; Spalding leg.; BPBM 21663 • 1; Nuuanu; Forbes, Cooke leg.; BPBM 21737 • 3; Nuuanu; Thwing leg.; BPBM 25013 • 9; Nuuanu; Thwing leg.; BPBM 25021 • 14; Nuuanu; BPBM 33638 • 1; Nuuanu; Cooke leg.; BPBM 42403 • 1; Nuuanu; Cooke leg.; BPBM 42404 • 1; Nuuanu; Cooke leg.; BPBM 42409 • 1; Nuuanu; Cooke leg.; BPBM 42410 • 1; Nuuanu; Cooke leg.; BPBM 42411 • 1; Nuuanu; BPBM 112369 • 37; Nuuanu; C.N. Forbes leg.; BPBM 169040 • 104; Nuuanu; Charles H. Merriam leg.; BPBM 191086 • 2; Nuuanu; Charles H. Merriam leg.; BPBM 191101 • 12; Nuuanu; Charles H. Merriam leg.; BPBM 191115 • 30; Nuuanu; Charles H. Merriam leg.; BPBM 191121 • 1; Nuuanu Pali; 20 Feb 1910; Forbes leg.; BPBM 20777 • 2; Nuuanu Pali; BPBM 166433 • 77; Nuuanu Ridge; 06 May 1903; Cooke leg.; BPBM 12693 • 57; Nuuanu Ridge; 06 May 1903; Cooke leg.; BPBM 12694 • 27; Nuuanu Ridge; 06 May 1903; Cooke leg.; BPBM 12695 • 1; Nuuanu Ridge; 10 May 1903; Cooke leg.; BPBM 12691 • 6; Nuuanu Ridge; 11 May 1903; Cooke leg.; BPBM 12696 • 112; Nuuanu Ridge; 21 May 1903; Cooke leg.; BPBM 12692 • 46; Nuuanu Ridge; 21 May 1903; Cooke leg.; BPBM 12697 • 40; Nuuanu Ridge; 21 May 1903; Cooke leg.; BPBM 12698 • 16; Nuuanu Ridge; 02 Jun 1903; Cooke leg.; BPBM 12699 • 24; Nuuanu Ridge; 04 Jun 1903; Cooke leg.; BPBM 12700 • 70; Nuuanu Ridge; 04 Jun 1903; Cooke leg.; BPBM 12701 • 68; Nuuanu Ridge; 04 Jun 1903; Cooke leg.; BPBM 12702 • 14; Nuuanu Ridge; 04 Jun 1903; Cooke leg.; BPBM 12703 • 2; Nuuanu Ridge; 04 Jun 1903; Cooke leg.; BPBM 12704 • 50; Nuuanu Ridge; 12 Jun 1903; Cooke leg.; BPBM 12705 • 66; Nuuanu Ridge; 12 Jun 1903; Cooke leg.; BPBM 12706 • 52; Nuuanu Ridge; 12 Jun 1903; Cooke leg.; BPBM 12707 • 47; Nuuanu Ridge; 12 Jun 1903; Cooke leg.; BPBM 12708 • 1; Nuuanu Ridge; 12 Jun 1903; Cooke leg.; BPBM 12709 • 35; Nuuanu Ridge; 16 Jun 1903; Cooke leg.; BPBM 12710 • 3; Nuuanu Ridge; 16 Jun 1903; Cooke leg.; BPBM 12711 • 35; Nuuanu Ridge; 19 Jun 1903; Cooke leg.; BPBM 12712 • 10; Nuuanu Ridge; 19 Jun 1903; Cooke leg.; BPBM 12713 • 13; Nuuanu Ridge; 19 Jun 1903; Cooke leg.; BPBM 12714 • 6; Nuuanu Ridge; 19 Jun 1903; Cooke leg.; BPBM 12715 • 76; Nuuanu Ridge; 22 Jun 1903; Cooke leg.; BPBM 12717 • 80; Nuuanu Ridge; 22 Jun 1903; Cooke leg.; BPBM 12718 • 12; Nuuanu Ridge; 27 Jun 1903; Cooke leg.; BPBM 12719 • 22; Nuuanu Ridge; 01 Jul 1903; Cooke leg.; BPBM 12720 • 33; Nuuanu Ridge; 01 Jul 1903; Cooke leg.; BPBM 12721 • 83; Nuuanu Ridge; 01 Jul 1903; Cooke leg.; BPBM 12722 • 282; Nuuanu Ridge; 01 Jul 1903; Cooke leg.; BPBM 12723 • 61; Nuuanu Ridge; 01 Jul 1903; Cooke leg.; BPBM 12724 • 64; Nuuanu Ridge; 01 Jul 1903; Cooke leg.; BPBM 12725 • 77; Nuuanu Ridge; 01 Jul 1903; Cooke leg.; BPBM 12726 • 49; Nuuanu Ridge; 01 Jul 1903; Cooke leg.; BPBM 12727 • 49; Nuuanu Ridge; 01 Jul 1903; Cooke leg.; BPBM 12728 • 38; Nuuanu Ridge; 01 Jul 1903; Cooke leg.; BPBM 12729 • 64; Nuuanu Ridge; 01 Jul 1903; Cooke leg.; BPBM 12730 • 80; Nuuanu Ridge; 21 Jan 1904; Cooke leg.; BPBM 12732 • 91; Nuuanu Ridge; 21 Jan 1904; Cooke leg.; BPBM 12733 • 67; Nuuanu Ridge; 21 Jan 1904; Cooke leg.; BPBM 12734 • 75; Nuuanu Ridge; 21 Jan 1904; Cooke leg.; BPBM 12735 • 80; Nuuanu Ridge; 21 Jan 1904; Cooke leg.; BPBM 12736 • 71; Nuuanu Ridge; 21 Jan 1904; Cooke leg.; BPBM 12737 • 1; Nuuanu Ridge; 16 Nov 1904; Cooke leg.; BPBM 12746 • 16; Nuuanu Ridge; 01 Mar 1907; Cooke leg.; BPBM 15337 • 25; Nuuanu Ridge; 01 Mar 1907; Cooke leg.; BPBM 15338 • 9; Nuuanu Ridge; 01 Mar 1907; Cooke leg.; BPBM 15339 • 5; Nuuanu Ridge; 01 Mar 1907; Cooke leg.; BPBM 15340 • 11; Nuuanu Ridge; 01 Mar 1907; Cooke leg.; BPBM 15341 • 167; Nuuanu Ridge; 22 Apr 1908; Cooke leg.; BPBM 16369 • 22; Nuuanu Ridge; 22 Apr 1908; Cooke leg.; BPBM 16386 • 5; Nuuanu Ridge; 05 May 1908; Cooke leg.; BPBM 16491 • 7; Nuuanu Ridge; 04 Jan 1912; A. Butt, Cooke leg.; BPBM 23648 • 17; Nuuanu Ridge; 16 Dec 1913; C.M. Cooke leg.; BPBM 34830 • 8; Nuuanu Ridge; 16 Dec 1913; Cooke leg.; BPBM 35476 • 6; Nuuanu Ridge; 28 Aug 1915; Emerson, J.S. leg.; BPBM 103107 • 2; Nuuanu Ridge; 19 Oct 1919; Bryan, E.H. leg.; BPBM 103108 • 4; Nuuanu Ridge; Cooke leg.; BPBM 12731 • 21; Nuuanu Ridge; Crampton, Cooke leg.; BPBM 20138 • 10; Nuuanu Ridge; Crampton, Cooke leg.; BPBM 20139 • 8; Nuuanu Ridge; Cooke, Ponsonby leg.; BPBM 23816 • 1; Nuuanu Ridge; Cooke leg.; BPBM 42407 • 1; Nuuanu Ridge; Cooke leg.; BPBM 42408 • 34; Nuuanu Valley; 01 Dec 1909; C.N. Forbes leg.; BPBM 169034 • 55; Nuuanu Valley; 26 Apr 1913; Emerson, J.S. leg.; BPBM 103105 • 2; Nuuanu Valley; 01 Sept 1914; BPBM 57078 • 6; Nuuanu Valley; 01 Sept 1914; BPBM 57087 • 26; Nuuanu Valley; 08 Sept 1914; BPBM 57054 • 4; Nuuanu Valley; 01 Oct 1914; BPBM 57049 • 17; Nuuanu Valley; 25 Oct 1914; BPBM 57035 • 15; Nuuanu Valley; 08 Nov 1914; BPBM 57039 • 21; Nuuanu Valley; 08 Nov 1914; BPBM 57062 • 26; Nuuanu Valley; 19 Nov 1914; J.S. Emerson leg.; BPBM 37840 • 130; Nuuanu Valley; 19 Nov 1914; W.G. Marshall leg.; BPBM 37848 • 7; Nuuanu Valley; 26 Dec 1914; Meinecke, William H. leg.; BPBM 120818 • 3; Nuuanu Valley; 22 Jun 1941; William H. Meinecke leg.; BPBM 194795 • 3; Nuuanu Valley; 22 Jun 1941; William H. Meinecke leg.; BPBM 194800 • 5; Nuuanu Valley; 11 Nov 1941; William H. Meinecke leg.; BPBM 194790 • 10; Nuuanu Valley; 11 Nov 1941; William H. Meinecke leg.; BPBM 194792 • 3; Nuuanu Valley; 23 Nov 1941; William H. Meinecke leg.; BPBM 194802 • 4; Nuuanu Valley; 23 Nov 1941; William H. Meinecke leg.; BPBM 194805 • 5; Nuuanu Valley; 30 Nov 1941; William H. Meinecke leg.; BPBM 194809 • 1; Nuuanu Valley; Forbes, Cooke leg.; BPBM 21691 • 1; Nuuanu Valley; Forbes, Cooke leg.; BPBM 21700 • 22; Nuuanu Valley; Forbes, Cooke leg.; BPBM 21701 • 1; Nuuanu Valley; Forbes, Cooke leg.; BPBM 21722 • 2; Nuuanu Valley; Cooke, Ponsonby leg.; BPBM 23829 • 15; Nuuanu Valley; Cooke leg.; BPBM 23941 • 18; Nuuanu Valley; BPBM 57038 • 68; Nuuanu Valley; BPBM 57041 • 40; Nuuanu Valley; BPBM 57042 • 102; Nuuanu Valley; BPBM 57051 • 58; Nuuanu Valley; BPBM 57052 • 87; Nuuanu Valley; BPBM 57059 • 46; Nuuanu Valley; BPBM 57060 • 10; Nuuanu Valley; BPBM 57069 • 39; Nuuanu Valley; BPBM 57070 • 2; Nuuanu Valley; BPBM 57093 • 76; Nuuanu Valley; BPBM 57097 • 8; Nuuanu Valley; C.N. Forbes leg.; BPBM 169036 • 20; Nuuanu Valley; C.N. Forbes leg.; BPBM 169037 • 17; Nuuanu-Pauoa; 01 Feb 1916; Spalding leg.; BPBM 41292 • 1; Nuuanu-Pauoa; 28 Jan 1923; W.H. Meinecke leg.; BPBM 53769 • 1; Nuuanu-Pauoa; 28 Jan 1923; Meinecke, William H. leg.; BPBM 120772 • 9; Nuuanu-Pauoa; 01 Mar 1941; W.J. Clench, Donald Anderson leg.; BPBM 189043 • 16; Nuuanu-Pauoa; 01 Mar 1941; W.J. Clench, Donald Anderson leg.; BPBM 189044 • 3; Nuuanu-Pauoa; 01 Mar 1941; W.J. Clench, Donald Anderson leg.; BPBM 189063 • 4; Nuuanu-Pauoa; 01 Mar 1941; W.J. Clench, Donald Anderson leg.; BPBM 189064 • 1; Nuuanu-Pauoa; 01 Mar 1941; W.J. Clench, Donald Anderson leg.; BPBM 189071 • 1; Nuuanu-Pauoa; F.B. Freitas leg.; BPBM 23704 • 7; Pacific Heights; Podmore leg.; BPBM 17531 • 41; Pacific Heights; Podmore leg.; BPBM 17532 • 1; Pacific Heights; Podmore leg.; BPBM 17533 • 25; Pacific Heights; Podmore leg.; BPBM 17534 • 4; Pacific Heights Ridge; 29 Oct 1922; Meinecke, William H. leg.; BPBM 120763 • 8; Pacific Heights Ridge; 02 May 1935; Donald Anderson , Yoshio Kondo leg.; BPBM 162328 • 28; Pali; Spalding leg.; BPBM 20462 • 3; Pali; C. Heatham leg.; BPBM 71298 • 20; Pali Road; 01 Jan 1915; A. Gouveia leg.; BPBM 40604 • 24; Pali Road; 28 Jun 1917; J.C. Bridwell leg.; BPBM 43940 • 1; Palolo; 27 May 1907; Cooke leg.; BPBM 15436 • 47; Palolo; 27 May 1907; Cooke leg.; BPBM 15437 • 12; Palolo; 03 May 1908; Spalding leg.; BPBM 16441 • 4; Palolo; 07 May 1910; Spalding leg.; BPBM 20951 • 35; Palolo; 01 Jun 1912; S.S.Emerson leg.; BPBM 24900 • 126; Palolo; 07 Jul 1915; Emerson, J.S. leg.; BPBM 103091 • 8; Palolo; 26 Sept 1915; A. Gouveia leg.; BPBM 40394 • 1; Palolo; 10 Oct 1915; Meinecke, William H. leg.; BPBM 120374 • 20; Palolo; 01 Jun 1917; J.C. Bridwell leg.; BPBM 43841 • 5; Palolo; 19 Nov 1919; Emerson, J.S. leg.; BPBM 103092 • 31; Palolo; 05 Apr 1920; C.F. Mant leg.; BPBM 49588 • 8; Palolo; 08 May 1920; Emerson, J.S. leg.; BPBM 103090 • 10; Palolo; 25 Nov 1921; Meinecke, William H. leg.; BPBM 120446 • 43; Palolo; 22 Mar 1931; Wilder, Hume, Christophersen leg.; BPBM 104525 • 7; Palolo; 04 Nov 1931; Cooke leg.; BPBM 12843 • 10; Palolo; 08 Nov 1932; Meinecke, William H. leg.; BPBM 120361 • 19; Palolo; 17 Jul 1940; Donald Anderson, Yoshio Kondo, Rokuro Yamaguchi leg.; BPBM 185636 • 10; Palolo; 17 Jul 1940; Donald Anderson, Yoshio Kondo, Rokuro Yamaguchi leg.; BPBM 185637 • 3; Palolo; 17 Jul 1940; Donald Anderson, Yoshio Kondo, Rokuro Yamaguchi leg.; BPBM 185638 • 102; Palolo; 18 Aug 1941; Staff, T. Tanada leg.; BPBM 212009 • 121; Palolo; 18 Aug 1941; Staff, T. Tanada leg.; BPBM 212010 • 88; Palolo; 18 Aug 1941; Staff, T. Tanada leg.; BPBM 212011 • 6; Palolo; 29 Sept 1941; William H. Meinecke leg.; BPBM 194772 • 16; Palolo; 25 May 1946; Donald Anderson, Yoshio Kondo leg.; BPBM 190854 • 81; Palolo; BPBM 15436 • 3; Palolo; Baldwin leg.; BPBM 18922 • 19; Palolo; Thwing leg.; BPBM 25011 • 114; Palolo; BPBM 56944 • 34; Palolo; Gulick leg.; BPBM 71082 • 50; Palolo; Gulick leg.; BPBM 71083 • 47; Palolo; Gulick leg.; BPBM 71084 • 2; Palolo; BPBM 105526 • 3; Palolo; A.B. Lyons and Others leg.; BPBM 185507 • 15; Palolo-Kekio-Pukele Stream; 20 Dec 1932; Russ, Glen W. leg.; BPBM 120004 • 11; Palolo-Kekio-Pukele Stream; 20 Dec 1932; Russ, Glen W. leg.; BPBM 120005 • 22; Pauoa; 06 Aug 1905; J. Waterhouse, Cooke leg.; BPBM 12848 • 1; Pauoa; 25 Nov 1907; Cooke leg.; BPBM 15840 • 3; Pauoa; 25 Jul 1909; Spalding leg.; BPBM 19751 • 2; Pauoa; 01 Dec 1911; F.B. Freitas leg.; BPBM 23694 • 4; Pauoa; 31 Dec 1911; Meinecke, William H. leg.; BPBM 120689 • 384; Pauoa; 01 Jun 1912; S.S.Emerson leg.; BPBM 24898 • 43; Pauoa; 01 Jun 1912; Meinecke, William H. leg.; BPBM 24899 • 56; Pauoa; 01 Aug 1912; Vasconcelles, M. leg.; BPBM 103104 • 15; Pauoa; 28 Aug 1912; O.H. Emerson leg.; BPBM 24901 • 3; Pauoa; 28 Aug 1912; R. Scudder leg.; BPBM 24909 • 2; Pauoa; 28 Aug 1912; S.S. Emerson leg.; BPBM 24915 • 8; Pauoa; 28 Aug 1912; S.S. Emerson leg.; BPBM 24921 • 30; Pauoa; 28 Aug 1912; S.S. Emerson leg.; BPBM 24922 • 5; Pauoa; 26 Apr 1913; J.S. Emerson, J.M. Cox leg.; BPBM 34021 • 13; Pauoa; 20 May 1913; J.S. Emerson leg.; BPBM 34063 • 14; Pauoa; 31 Mar 1914; J.S. Emerson leg.; BPBM 36231 • 20; Pauoa; 24 May 1914; Meinecke, William H. leg.; BPBM 120499 • 11; Pauoa; 18 Jul 1914; L.A. Thurston leg.; BPBM 37038 • 5; Pauoa; 08 Nov 1914; BPBM 57016 • 5; Pauoa; 01 Apr 1917; A. Gouveia leg.; BPBM 43600 • 21; Pauoa; 18 Oct 1919; Bryan, E.H. leg.; BPBM 49053 • 13; Pauoa; 18 Oct 1919; Bryan, E.H. leg.; BPBM 49056 • 1; Pauoa; 04 Jan 1920; D.W. Garber leg.; BPBM 184836 • 1; Pauoa; 11 Apr 1920; D. Wesley Garber leg.; BPBM 58820 • 69; Pauoa; 11 Apr 1920; D. Wesley Garber leg.; BPBM 58821 • 6; Pauoa; 11 Sept 1927; Meinecke, William H. leg.; BPBM 120757 • 8; Pauoa; 10 Apr 1928; Oswald, Olaf leg.; BPBM 90363 • 6; Pauoa; 10 Apr 1928; Oswald, Olaf leg.; BPBM 90364 • 7; Pauoa; 10 Apr 1928; Oswald, Olaf leg.; BPBM 90371 • 1; Pauoa; 10 Apr 1928; Oswald, Olaf leg.; BPBM 90372 • 55; Pauoa; 23 Dec 1928; Meinecke, William H. leg.; BPBM 120726 • 698; Pauoa; 05 Jan 1929; Russ, Glen W. leg.; BPBM 133972 • 9; Pauoa; 19 Jan 1930; Meinecke, William H. leg.; BPBM 120837 • 6; Pauoa; 11 Oct 1930; Oswald, Olaf leg.; BPBM 100698 • 4; Pauoa; 11 Oct 1930; Oswald, Olaf leg.; BPBM 100699 • 18; Pauoa; 11 Oct 1930; Oswald, Olaf leg.; BPBM 100700 • 9; Pauoa; 11 Oct 1930; Oswald, Olaf leg.; BPBM 100701 • 16; Pauoa; 11 Oct 1930; Oswald, Olaf leg.; BPBM 100704 • 6; Pauoa; 11 Oct 1930; Oswald, Olaf leg.; BPBM 100705 • 17; Pauoa; 11 Oct 1930; Oswald, Olaf leg.; BPBM 100706 • 11; Pauoa; 11 Oct 1930; Oswald, Olaf leg.; BPBM 100707 • 12; Pauoa; Cooke leg.; BPBM 12819 • 9; Pauoa; Spalding leg.; BPBM 17790 • 2; Pauoa; Spalding leg.; BPBM 17791 • 53; Pauoa; Spalding leg.; BPBM 17904 • 9; Pauoa; Spalding leg.; BPBM 19753 • 5; Pauoa; Spalding leg.; BPBM 20816 • 23; Pauoa; BPBM 57010 • 4; Pauoa; BPBM 57012 • 16; Pauoa; BPBM 57018 • 43; Pauoa; BPBM 57030 • 67; Pauoa; Bryan, E.H. leg.; BPBM 103102 • 465; Pauoa; Emerson, J.S., O.H.E. leg.; BPBM 103103 • 39; Pauoa; Judd, Alfred F. leg.; BPBM 109276 • 105; Pauoa; Meinecke, William H. leg.; BPBM 120715 • 112; Pauoa; Meinecke, William H. leg.; BPBM 120716 • 3; Pauoa; A.B. Lyons and Others leg.; BPBM 185506 • 6; Pauoa; A.B. Lyons and Others leg.; BPBM 185508 • 16; Pukele Stream; 18 May 1946; Yoshio Kondo, Others leg.; BPBM 190850 • 1; Puu Lanihuli; 28 Jun 1908; J.F. Stokes leg.; BPBM 16686 • 1; Puu Lanihuli; 09 Feb 1919; J.C. Bridwell leg.; BPBM 47160 • 2; Puu Lanihuli; Spalding leg.; BPBM 19582 • 1; Puu O Kona; 05 Jul 1936; C. Hartt leg.; BPBM 183681 • 1; Tantalus; 07 Dec 1907; Cooke leg.; BPBM 15882 • 2; Tantalus; 10 Dec 1907; Cooke leg.; BPBM 15903 • 33; Tantalus; 01 Mar 1908; Cooke leg.; BPBM 16193 • 1; Tantalus; 08 Apr 1908; Cooke leg.; BPBM 16246 • 32; Tantalus; 10 Apr 1910; Spalding leg.; BPBM 20838 • 6; Tantalus; 02 Aug 1912; Lyons, A.B. leg.; BPBM 103100 • 1; Tantalus; 28 Aug 1912; S.S. Emerson leg.; BPBM 24919 • 117; Tantalus; 26 Apr 1913; Emerson, J.S. leg.; BPBM 103098 • 13; Tantalus; 20 May 1913; J.S. Emerson leg.; BPBM 34074 • 102; Tantalus; 20 May 1913; Emerson, J.S. leg.; BPBM 103097 • 21; Tantalus; 10 Jun 1913; J.S. Emerson leg.; BPBM 34762 • 116; Tantalus; 30 Jun 1913; J.S. Emerson leg.; BPBM 34107 • 26; Tantalus; 30 Jun 1913; J.S. Emerson leg.; BPBM 34109 • 31; Tantalus; 30 Jun 1913; J.S. Emerson leg.; BPBM 34115 • 6; Tantalus; 01 Apr 1914; J.S. Emerson leg.; BPBM 36217 • 1; Tantalus; 01 Apr 1914; J.S. Emerson leg.; BPBM 36222 • 7; Tantalus; 04 Apr 1914; J.S. Emerson leg.; BPBM 36233 • 16; Tantalus; 28 Sept 1919; Vasconcelles, M. leg.; BPBM 103101 • 5; Tantalus; 11 Apr 1920; D. Wesley Garber leg.; BPBM 58819 • 47; Tantalus; 01 Jan 1922; Wm. D. Wilder leg.; BPBM 51415 • 6; Tantalus; 08 Sept 1923; S.C. Ball leg.; BPBM 76844 • 1; Tantalus; 19 Nov 1924; N.H. Goalstone leg.; BPBM 79756 • 16; Tantalus; 01 Jan 1935; Ernest E. Lyman leg.; BPBM 170307 • 11; Tantalus; 01 Jan 1935; Ernest E. Lyman leg.; BPBM 170308 • 13; Tantalus; 01 Jan 1935; Ernest E. Lyman leg.; BPBM 170309 • 7; Tantalus; 08 Aug 1941; T. Tanada, Yoshio Kondo leg.; BPBM 211985 • 3; Tantalus; 08 Aug 1941; T. Tanada, Yoshio Kondo leg.; BPBM 211986 • 6; Tantalus; 08 Aug 1941; T. Tanada, Yoshio Kondo leg.; BPBM 211991 • 4; Tantalus; 08 Aug 1941; T. Tanada, Yoshio Kondo leg.; BPBM 211992 • 4; Tantalus; 09 Jun 1943; Y. Tanada leg.; BPBM 285783 • 2; Tantalus; 09 Jun 1943; Y. Tanada leg.; BPBM 189710 • 2; Tantalus; 09 Jun 1943; Y. Tanada leg.; BPBM 189711 • 177; Tantalus; BPBM 16246 • 6; Tantalus; Baldwin leg.; BPBM 18921 • 8; Tantalus; Thwing leg.; BPBM 25007 • 1; Tantalus; Thwing leg.; BPBM 25015 • 18; Tantalus; Thwing leg.; BPBM 25024 • 218; Tantalus; J.S. Emerson, J.M. Cox leg.; BPBM 34025 • 15; Tantalus; D.D. Baldwin leg.; BPBM 55833 • 1; Tantalus; D.D. Baldwin leg.; BPBM 55834 • 18; Tantalus; BPBM 56992 • 2; Tantalus; BPBM 56995 • 1; Tantalus; BPBM 57006 • 102; Tantalus; Emerson, J.S. leg.; BPBM 103096 • 125; Tantalus; Emerson, J.S., O.H.E. leg.; BPBM 103099 • 4; Tantalus; A.B. Lyons and Others leg.; BPBM 185504 • 2; Tantalus; A.B. Lyons and Others leg.; BPBM 185505 • 6; Tantalus; A.B. Lyons and Others leg.; BPBM 185509 • 6; Tantalus-Pauoa Flats; 10 Jun 1917; J.C. Bridwell leg.; BPBM 43854 • 2; Tantalus-Pauoa Flats; 10 Jun 1917; J.C. Bridwell leg.; BPBM 43858 • 12; Tantalus-Pauoa Flats; 17 Jun 1917; J.C. Bridwell leg.; BPBM 43882 • 5; Tantalus-Pauoa Flats; 17 Jun 1917; J.C. Bridwell leg.; BPBM 43883 • 12; Tantalus-Pauoa Flats; 17 Jun 1917; J.C. Bridwell leg.; BPBM 43888 • 6; Tantalus-Pauoa Flats; 17 Jun 1917; J.C. Bridwell leg.; BPBM 43890 • 8; Tantalus-Pauoa Flats; Spalding leg.; BPBM 19885 • 6; Tantalus-Pauoa Flats; BPBM 23036 • 2; Waialae; Cooke leg.; BPBM 12841 • 1; Waialae; BPBM 32183 • 2; Waialae Iki; 08 Apr 1917; J.C. Bridwell leg.; BPBM 43781 • 4; Waialae Iki; 08 Apr 1917; J.C. Bridwell leg.; BPBM 43784 • 4; Waialae Iki; 08 Apr 1917; J.C. Bridwell leg.; BPBM 43798 • 7; Waialae Iki; 08 Apr 1917; J.C. Bridwell leg.; BPBM 43804 • 26; Waiau-Waimalu; 08 Dec 1931; Russ, Glen W. leg.; BPBM 134108 • 8; Waiomao Valley; 28 Oct 1920; Emerson, J.S. leg.; BPBM 103088 • 6; Waipakiki Valley; 29 Mar 1915; J.S. Emerson leg.; BPBM 38392 • 6; Waipakiki Valley; 08 May 1915; J.S. Emerson leg.; BPBM 38413 • 11; Waipakiki Valley; 08 May 1915; Marcel H.J. Desnouee leg.; BPBM 38429 • 6; Waipakiki Valley; 08 May 1915; Emerson, J.S. leg.; BPBM 103110 • 269; Waipakiki Valley; Emerson, J.S. leg.; BPBM 103109.

*Auriculella minuta* Cooke and Pilsbry, 1915

USA – Honolulu County, Oahu, Koolau Mountains • 1; Kalihi Valley; Forbes leg.; BPBM 20198 • 1; Kalihi Valley; 05 Jun 1918; Emerson, J.S. leg.; BPBM 103193 • 1; Kalihi Valley; 05 Jun 1918; C.F. Mant leg.; BPBM 46017 • 2; Kalihi Valley; 25 Jan 1931; Christophersen, Wilder, Hume leg.; BPBM 161466 • 2; Kalihi Valley; BPBM 99164-5 • 6; Kalihi Valley; 11 Jun 1930; Oswald, Olaf leg.; BPBM 99165 • 9; Kalihi Valley; 25 Jan 1931; Wilder, Hume, Christophersen leg.; BPBM 104485 • 14; Kalihi Valley; 01 May 1923; C.V. Budd leg.; BPBM 54628 • 21; Kalihi Valley; 11 Jun 1930; Oswald, Olaf leg.; BPBM 99164 • 7; Manoa; 25 Nov 1928; Meinecke, William H. leg.; BPBM 93116 • 1; Manoa-Palolo; Judd, Alfred F. leg.; BPBM 109208 • 1; Manoa-Palolo; Meinecke, William H. leg.; BPBM 9587 • 2; Manoa-Palolo; BPBM 134859 • 3; Manoa-Palolo; 29 Mar 1936; Robert L. Usinger leg.; BPBM 166399 • 6; Manoa-Palolo; 25 Nov 1928; Meinecke, William H. leg.; BPBM 120564 • 14; Manoa-Palolo; 14 Sept 1941; William H. Meinecke, Meadows, BPBM Malacology Staff leg.; BPBM 194673 • 1; Mauna Kope; 22 Mar 1930; Oswald, Olaf leg.; BPBM 97936 • 2; Mauna Kope; BPBM 97935-7 • 5; Mauna Kope; 22 Mar 1930; Oswald, Olaf leg.; BPBM 97935 • 9; Mauna Kope; 22 Mar 1930; Oswald, Olaf leg.; BPBM 97937 • 3; Moanalua; 01 Jun 1930; Oswald, Olaf leg.; BPBM 99139 • 2; Mount Independence; 22 Aug 1903; Cooke leg.; BPBM 12792 • 2; Mount Olympus; 14 Apr 1908; Cooke leg.; BPBM 16288 • 1; Nuuanu; 28 Mar 1943; William H. Meinecke leg.; BPBM 189896 • 2; Nuuanu; BPBM 42379 • 2; Nuuanu; 25 Nov 1907; Cooke leg.; BPBM 15839 • 3; Nuuanu; 04 Jan 1912; A. Butt, Cooke leg.; BPBM 23656 • 4; Nuuanu; BPBM 42379-83 • 5; Nuuanu; 01 Jan 1935; BPBM 170314 • 23; Nuuanu; BPBM 16371 • 1; Nuuanu Pali; 21 Mar 1943; William H. Meinecke leg.; BPBM 189888 • 1; Nuuanu Ridge; BPBM 13034 • 1; Nuuanu Ridge; BPBM 13035 • 1; Nuuanu Ridge; Cooke leg.; BPBM 42380 • 2; Nuuanu Ridge; 05 May 1908; Cooke leg.; BPBM 16490 • 2; Nuuanu Ridge; 19 Jun 1903; Cooke leg.; BPBM 12803 • 3; Nuuanu Ridge; 19 Jun 1903; Cooke leg.; BPBM 12802 • 4; Nuuanu Ridge; Cooke, Ponsonby leg.; BPBM 23818 • 6; Nuuanu Ridge; 05 May 1908; Cooke leg.; BPBM 16480 • 7; Nuuanu Ridge; 01 Aug 1903; Cooke leg.; BPBM 12806 • 11; Nuuanu Ridge; 01 Aug 1903; Cooke leg.; BPBM 12807 • 16; Nuuanu Ridge; 04 Jun 1903; Cooke leg.; BPBM 12797 • 24; Nuuanu Ridge; 19 Jun 1903; Cooke leg.; BPBM 12801 • 27; Nuuanu Ridge; 04 Jun 1903; Cooke leg.; BPBM 12795 • 33; Nuuanu Ridge; Cooke leg.; BPBM 42381 • 104; Nuuanu Ridge; 08 Jun 1903; Cooke leg.; BPBM 12799 • 151; Nuuanu Ridge; 01 Aug 1903; Cooke leg.; BPBM 12804 • 1; Nuuanu Valley; 28 Nov 1911; H. Hatch, A.J. Cooke, C.M. Cooke leg.; BPBM 23425 • 1; Nuuanu Valley; Cooke leg.; BPBM 42382 • 1; Nuuanu Valley; Cooke leg.; BPBM 42377 • 1; Nuuanu Valley; Cooke leg.; BPBM 42378 • 2; Nuuanu Valley; Forbes, Cooke leg.; BPBM 21702 • 3; Nuuanu Valley; Cooke, Ponsonby leg.; BPBM 23830 • 5; Nuuanu Valley; Cooke, Ponsonby leg.; BPBM 23839 • 82; Nuuanu Valley; Cooke leg.; BPBM 42383 • 223; Nuuanu Valley; Cooke leg.; BPBM 42379 • 506; Nuuanu Valley; 22 Apr 1908; Cooke leg.; BPBM 16371 • 1; Nuuanu-Konahuanui; 20 Mar 1928; Oswald, Olaf leg.; BPBM 90118 • 20; Palolo; 01 Jan 1935; Ernest E. Lyman leg.; BPBM 170304 • 1; Palolo Valley; BPBM 55825 • 5; Palolo Valley; 18 Aug 1941; BPBM Malacology Staff, T. Tanada leg.; BPBM 212012 • 5; Palolo Valley; Lyman leg.; BPBM 12808 • 6; Palolo Valley; BPBM 16435 • 7; Puu Lanihuli; 09 Feb 1919; J.C. Bridwell leg.; BPBM 47162 • 1; Puu Ohulehule; 01 Sept 1923; C.S. Judd leg.; BPBM 76840 • 1; Puu Ohulehule; 31 Jul 1929; Oswald, Olaf leg.; BPBM 96588 • 1; Puu Ohulehule; 23 Jun 1928; Oswald, Olaf leg.; BPBM 91183 • 2; Puu Ohulehule; BPBM 98043-5 • 2; Puu Ohulehule; 23 Jun 1928; Oswald, Olaf leg.; BPBM 91186 • 3; Puu Ohulehule; 20 Apr 1930; Oswald, Olaf leg.; BPBM 98044 • 3; Puu Ohulehule; 20 Apr 1930; Oswald, Olaf leg.; BPBM 98045 • 5; Puu Ohulehule; 23 Jun 1928; Oswald, Olaf leg.; BPBM 91185 • 9; Puu Ohulehule; 01 Sept 1923; C.S. Judd leg.; BPBM 76841 • 13; Puu Ohulehule; 20 Apr 1930; Oswald, Olaf leg.; BPBM 98043 • 1; Waiahole Ridge; 06 Feb 1912; Forbes, Judd, Cooke leg.; BPBM 23629 • 2; Waiahole Ridge; 19 Apr 1931; Oswald, Olaf leg.; BPBM 104440 • 3; Waiahole Ridge; 19 Apr 1931; Oswald, Olaf leg.; BPBM 104439 • 4; Waiahole Ridge; 19 Apr 1931; Oswald, Olaf leg.; BPBM 104446 • 5; Waiahole Ridge; 01 Jan 1922; Wm. D. Wilder leg.; BPBM 51485 • 5; Waiahole Ridge; 19 Apr 1931; Oswald, Olaf leg.; BPBM 104436 • 5; Waiahole Ridge; 19 Apr 1931; Oswald, Olaf leg.; BPBM 104437 • 5; Waiahole Ridge; 19 Apr 1931; Oswald, Olaf leg.; BPBM 104438 • 16; Waiahole Ridge; BPBM 22803 • 2; Waikane Ridge; BPBM 98070-2 • 3; Waikane Ridge; 27 Apr 1930; Oswald, Olaf leg.; BPBM 98071 • 3; Waikane Ridge; 27 Apr 1930; Oswald, Olaf leg.; BPBM 98072 • 8; Waikane Ridge; 27 Apr 1930; Oswald, Olaf leg.; BPBM 98070.

*Auriculella perpusilla* E. Smith, 1873

USA – Honolulu County, Oahu, Waianae Mountains • 12; Koolau; BPBM 25039 • 17; Kaaawa; BPBM 15049 • 12; Kahana; BPBM 15048 • 1; Kaipapau-Koloa; 01 Jun 1948; BPBM 11865 • 7; Kaliuwaa; BPBM 55858 • 2; Koloa-Wailele; 01 Jun 1948; BPBM 11868 • 43; Koloa-Wailele; 01 Jun 1948; BPBM 11869 • 23; Waiahole; BPBM 22802 • 1; Kahauiki; 21 May 1916; A. Gouveia leg.; BPBM 41843 • 14; Kalihi; 14 Nov 1915; A. Gouveia leg.; BPBM 40531 • 22; Kalihi; 27 Nov 1915; A. Gouveia leg.; BPBM 40556 • 41; Kalihi; 27 May 1917; A. Gouveia leg.; BPBM 43670 • 1; Kamanaiki; 28 May 1916; A. Gouveia leg.; BPBM 41852 • 4; Kamanaiki; 24 Oct 1915; A. Gouveia leg.; BPBM 40456 • 1; Moanalua; 23 Nov 1919; A. Gouveia leg.; BPBM 49155 • 15; Moanalua; 23 Nov 1919; A. Gouveia leg.; BPBM 49150 • 1; Kaliuwaa; A.F. Judd leg.; BPBM 12789 • 5; Kaluanui; 27 Aug 1933; Anderson, Donald leg.; BPBM 129498 • 1; Malaekahana; 06 Apr 1917; B. Olivera, C.M.C. III, C.M. Cooke leg.; BPBM 44762 • 4; Kahana; Baldwin leg.; BPBM 18934 • 4; Moanalua; 08 Feb 1920; Bryan leg.; BPBM 49060 • 21; Pupukea; 23 Jan 1929; Bryan, Neal leg.; BPBM 93636 • 21; Pupukea; 23 Jan 1929; Bryan, Neal leg.; BPBM 93637 • 5; Kaliuwaa Valley (Sacred Falls Valley); 14 Apr 1929; Bryan, Neal, Whiting leg.; BPBM 93627 • 7; Kaliuwaa Valley (Sacred Falls Valley); 14 Apr 1929; Bryan, Neal, Whiting leg.; BPBM 93625 • 7; Kaliuwaa Valley (Sacred Falls Valley); 14 Apr 1929; Bryan, Neal, Whiting leg.; BPBM 93630 • 8; Kaliuwaa Valley (Sacred Falls Valley); 14 Apr 1929; Bryan, Neal, Whiting leg.; BPBM 93628 • 18; Kaliuwaa Valley (Sacred Falls Valley); 14 Apr 1929; Bryan, Neal, Whiting leg.; BPBM 93629 • 27; Kaliuwaa Valley (Sacred Falls Valley); 14 Apr 1929; Bryan, Neal, Whiting leg.; BPBM 93626 • 29; Kaliuwaa Valley (Sacred Falls Valley); 14 Apr 1929; Bryan, Neal, Whiting leg.; BPBM 93631 • 3; Kalihi; 29 May 1918; C.F. Mant leg.; BPBM 45975 • 17; Kalihi Ridge; 29 May 1918; C.F. Mant leg.; BPBM 45964 • 1; Kawailoa; 05 Apr 1917; C.M. Cooke III, C.M. Cooke leg.; BPBM 44964 • 5; Kahuku; 15 Sep 1921; C.M. Cooke Jr., A.T. Spalding leg.; BPBM 51952 • 1; Malaekahana; 03 Feb 1917; C.M. Cooke, Jr., C.M. Cooke, III. leg.; BPBM 52927 • 22; Puu Ohulehule; 01 Sep 1923; C.S. Judd leg.; BPBM 76842 • 22; Puu Ohulehule; 01 Sep 1923; C.S. Judd leg.; BPBM 76843 • 2; Malaekahana; 24 Nov 1915; Carolene, Charley & C.M. Cooke leg.; BPBM 41015 • 1; Kalihi Valley; 25 Jan 1931; Christopherson, Wilder & Hume leg.; BPBM 185585 • 1; Punaluu; 08 Aug 1917; B. Olivera, C.M.C. III, C.M. Cooke leg.; BPBM 45144 • 1118; Kaipapau; 05 Aug 1909; Cooke leg.; BPBM 19815 • 202; Kaliuwaa; Cooke leg.; BPBM 19833 • 19; Koolau; Cooke leg.; BPBM 21527 • 20; Kaipapau; Cooke, Forbes leg.; BPBM 19320 • 4; Kaipapau; Cooke, Forbes leg.; BPBM 19300 • 1; Kaliuwaa; Cooke, Forbes leg.; BPBM 19350 • 64; Kaliuwaa; Cooke, Forbes leg.; BPBM 19344 • 207; Punaluu; 04 May 1909; Cooke, Forbes leg.; BPBM 19200 • 1; Kahuku; 18 Jan 1913; Cooke, Pilsbry leg.; BPBM 45244 • 81; Punaluu; 01 Oct 1912; Cooke, L.L. Cooke leg.; BPBM 24943 • 1; Kaluakauila-Malaekahana Ridge; 15 Apr 1933; Donald Anderson leg.; BPBM 163951 • 5; Kaluakauila-Malaekahana Ridge; 15 Apr 1933; Donald Anderson leg.; BPBM 163956 • 19; Kaluakauila-Malaekahana Ridge; 15 Apr 1933; Donald Anderson leg.; BPBM 163959 • 7; Kamanaiki; 07 Jun 1936; Donald Anderson, Yoshio Kondo leg.; BPBM 162376 • 7; Punaluu; 14 Feb 1939; Donald Anderson, Yoshi Kondo, R. Yamaguchi leg.; BPBM 180768 • 23; Punaluu; 14 Feb 1939; Donald Anderson, Yoshi Kondo, R. Yamaguchi leg.; BPBM 180767 • 8; Kahana; 17 Jan 1946; Donald V. Hemphill leg.; BPBM 190390 • 4; Kipapa-Waiawa Ridge; 14 Apr 1946; E. Nishimura, Y. Tanada, Yoshio Kondo leg.; BPBM 190866 • 16; Kipapa-Waiawa Ridge; 14 Apr 1946; E. Nishimura, Y. Tanada, Yoshio Kondo leg.; BPBM 190862 • 5; Kahana-Waikane; 04 Sep 1927; E.H. Bryan, Jr. leg.; BPBM 88974 • 2; Moanalua; 09 Apr 1922; E.H. Bryan, Jr. leg.; BPBM 60026 • 13; Haiku-Iolekaa; 12 Apr 1936; Edmund J. Meadows leg.; BPBM 166388 • 6; Waikane; 22 Oct 1947; Elwood C. Zimmerman leg.; BPBM 210527 • 3; Punaluu; 09 Jul 1920; Emerson, J.S. leg.; BPBM 103204 • 12; Punaluu; 06 Apr 1917; Emerson, J.S., O.H.E. leg.; BPBM 103205 • 2; Kahana; 01 Jan 1935; Ernest E. Lyman leg.; BPBM 170402 • 109; Kahana; 01 Jan 1935; Ernest E. Lyman leg.; BPBM 170328 • 1; Punaluu; Forbes leg.; BPBM 19369 • 1; Waiahole; 06 Feb 1912; Forbes, Judd, Cooke leg.; BPBM 23628 • 1; Kahana; 02 Dec 1934; Fosberg, F. Raymond leg.; BPBM 132663 • 21; Kipapa Gulch; 04 Jul 1932; Hosaka, Edward Y. leg.; BPBM 114930 • 21; Kipapa Gulch; 04 Jul 1932; Hosaka, Edward Y. leg.; BPBM 114932 • 10; Ahuimanu; 12 Dec 1915; J. Gouveia leg.; BPBM 40567 • 19; Kalihi; 29 May 1918; J.S. Emerson leg.; BPBM 45769 • 5; Kalihi; 21 Sep 1918; J.S. Emerson leg.; BPBM 45793 • 57; Kalihi; 21 Nov 1918; J.S. Emerson leg.; BPBM 45806 • 6; Kalihi Ridge; 01 Apr 1913; J.S. Emerson leg.; BPBM 34034 • 8; Kaliuwaa; Judd, Alfred F. leg.; BPBM 109372 • 64; Punaluu; 01 Oct 1912; L.L., C.M. Cooke leg.; BPBM 24959 • 1; Kahana; Lyman leg.; BPBM 42414 • 1; Kahana; Lyman leg.; BPBM 42415 • 3; Kahana; Lyman leg.; BPBM 12809 • 1; Halawa Barrel Trail; 23 May 1937; M., Donald Anderson leg.; BPBM 164045 • 4; Halawa Barrel Trail; 23 May 1937; M., Donald Anderson leg.; BPBM 164109 • 5; Halawa Barrel Trail; 23 May 1937; M., Donald Anderson leg.; BPBM 164108 • 21; Halawa Barrel Trail; 23 May 1937; M., Donald Anderson leg.; BPBM 164105 • 21; Halawa Barrel Trail; 23 May 1937; M., Donald Anderson leg.; BPBM 164106 • 21; Halawa Barrel Trail; 23 May 1937; M., Donald Anderson leg.; BPBM 164107 • 2; Halawa Valley; 23 May 1937; M., Donald Anderson leg.; BPBM 164090 • 4; Halawa Valley; 23 May 1937; M., Donald Anderson leg.; BPBM 164089 • 1; Kahana; 04 Jul 1920; M.C. Neal leg.; BPBM 52029 • 1; Kalihi Ridge; 01 May 1921; M.C. Neal, Mr. Thrum leg.; BPBM 58926 • 15; Kaliuwaa Valley (Sacred Falls Valley); 01 Jun 1930; McAllister (Mrs.) leg.; BPBM 98858 • 31; Kaliuwaa Valley (Sacred Falls Valley); 01 Jun 1930; McAllister (Mrs.) leg.; BPBM 98857 • 14; Kawailoa; 11 Apr 1935; Meadow, Welch leg.; BPBM 132151 • 1; Iolekaa Valley; 20 Nov 1932; Meadows, E.J. leg.; BPBM 114900 • 2; Kahauiki-Kalihi; 15 Apr 1927; W.H. Meinecke leg.; BPBM 93479 • 7; Hauula; 27 Nov 1925; W.H. Meinecke leg.; BPBM 93068 • 10; Kipapa Ridge; 07 Apr 1929; W.H. Meinecke leg.; BPBM 93164 • 13; Kipapa Ridge; 07 Apr 1929; W.H. Meinecke leg.; BPBM 93163 • 2; Halawa; 17 Apr 1930; W.H. Meinecke leg.; BPBM 121043 • 3; Halawa; 02 Mar 1930; W.H. Meinecke leg.; BPBM 120988 • 1; Kawaipapa-Maakua; 16 Jun 1935; W.H. Meinecke leg.; BPBM 132405 • 1; Kawaipapa-Maakua; 16 Jun 1935; W.H. Meinecke leg.; BPBM 132409 • 7; Maakua-Papali; 27 Nov 1925; W.H. Meinecke leg.; BPBM 122591 • 1; Papali-Punaiki; 15 Jul 1934; W.H. Meinecke leg.; BPBM 128830 • 2; Kaluanui-Maakua; 15 Jul 1934; W.H. Meinecke leg.; BPBM 128840 • 2; Punaiki; 02 Dec 1934; W.H. Meinecke leg.; BPBM 132360 • 34; Iolekaa Valley; 20 Nov 1932; W.H. Meinecke leg.; BPBM 122780 • 40; Iolekaa Valley; 20 Nov 1932; W.H. Meinecke leg.; BPBM 122779 • 24; Kaaawa; 08 May 1932; W.H. Meinecke leg.; BPBM 122643 • 6; Kahana-Punaluu; W.H. Meinecke leg.; BPBM 108719 • 29; Kahana-Punaluu; W.H. Meinecke leg.; BPBM 108718 • 4; Kaipapau-Koloa; 21 Apr 1935; W.H. Meinecke leg.; BPBM 132443 • 2; Kaiwikoele; 15 Mar 1932; W.H. Meinecke leg.; BPBM 122467 • 1; Kaluanui; 29 Jul 1934; W.H. Meinecke leg.; BPBM 128897 • 84; Ihiihi-Kahawainui; 10 Apr 1932; W.H. Meinecke leg.; BPBM 122567 • 119; Kaluakauila-Malaekahana; 03 Apr 1932; W.H. Meinecke leg.; BPBM 122560 • 10; Oio Stream; 26 Nov 1933; W.H. Meinecke leg.; BPBM 125015 • 43; Oio Stream; 26 Nov 1933; W.H. Meinecke leg.; BPBM 125013 • 43; Kawela-Oio-Pahipahialua Ridge; 14 May 1933; W.H. Meinecke leg.; BPBM 123845 • 1; Kaukonahua; 19 Jun 1932; W.H. Meinecke leg.; BPBM 122661 • 2; Kipapa-Waiawa; 07 Apr 1929; W.H. Meinecke leg.; BPBM 121898 • 16; Kalihi; 21 Nov 1918; Miss M. Burbank leg.; BPBM 45810 • 28; Kalihi; 21 Nov 1918; Miss M. Clough leg.; BPBM 45819 • 2; Hauula; 05 May 1928; O. Oswald leg.; BPBM 90732 • 3; Hauula; 06 Apr 1928; O. Oswald leg.; BPBM 90305 • 3; Hauula; 06 Apr 1928; O. Oswald leg.; BPBM 90296 • 4; Hauula; 05 May 1928; O. Oswald leg.; BPBM 90731 • 7; Hauula; 05 May 1928; O. Oswald leg.; BPBM 90708 • 7; Hauula; 05 May 1928; O. Oswald leg.; BPBM 90730 • 8; Hauula; 05 May 1928; O. Oswald leg.; BPBM 90718 • 9; Hauula; 06 Apr 1928; O. Oswald leg.; BPBM 90297 • 14; Hauula; 05 May 1928; O. Oswald leg.; BPBM 90707 • 20; Hauula; 06 Apr 1928; O. Oswald leg.; BPBM 90304 • 20; Hauula; 06 Apr 1928; O. Oswald leg.; BPBM 90310 • 20; Hauula; 06 Apr 1928; O. Oswald leg.; BPBM 90309 • 20; Hauula; 06 Apr 1928; O. Oswald leg.; BPBM 90298 • 22; Kaaawa; 21 May 1928; O. Oswald leg.; BPBM 90853 • 22; Kaaawa; 21 May 1928; O. Oswald leg.; BPBM 90854 • 5; Kaaawa; 21 May 1928; O. Oswald leg.; BPBM 90826 • 6; Kaaawa; 21 May 1928; O. Oswald leg.; BPBM 90848 • 6; Kaaawa; 21 May 1928; O. Oswald leg.; BPBM 90827 • 1; Weliweli; 17 Jun 1928; O. Oswald leg.; BPBM 91127 • 22; Puu Ohulehule; 23 Jun 1928; O. Oswald leg.; BPBM 91189 • 22; Puu Ohulehule; 23 Jun 1928; O. Oswald leg.; BPBM 91188 • 1; Kahana; 02 Jun 1928; O. Oswald leg.; BPBM 90886 • 1; Kahana; 24 Jun 1928; O. Oswald leg.; BPBM 91191 • 1; Kahana; 08 Jul 1928; O. Oswald leg.; BPBM 91316 • 1; Kahana; 08 Jul 1928; O. Oswald leg.; BPBM 91322 • 1; Kahana; 08 Sep 1928; O. Oswald leg.; BPBM 91368 • 1; Kahana; 08 Sep 1928; O. Oswald leg.; BPBM 91395 • 2; Kahana; 01 Jul 1928; O. Oswald leg.; BPBM 91267 • 2; Kahana; 22 Sep 1928; O. Oswald leg.; BPBM 91536 • 4; Kahana; 01 Jul 1928; O. Oswald leg.; BPBM 91275 • 4; Kahana; 08 Jul 1928; O. Oswald leg.; BPBM 91296 • 5; Kahana; 02 Jun 1928; O. Oswald leg.; BPBM 90906 • 5; Kahana; 02 Jun 1928; O. Oswald leg.; BPBM 90907 • 5; Kahana; 10 Jun 1928; O. Oswald leg.; BPBM 91000 • 5; Kahana; 24 Jun 1928; O. Oswald leg.; BPBM 91217 • 5; Kahana; 16 Sep 1928; O. Oswald leg.; BPBM 91492 • 6; Kahana; 02 Jun 1928; O. Oswald leg.; BPBM 90927 • 6; Kahana; 02 Jun 1928; O. Oswald leg.; BPBM 90936 • 8; Kahana; 01 Jul 1928; O. Oswald leg.; BPBM 91274 • 8; Kahana; 16 Sep 1928; O. Oswald leg.; BPBM 91484 • 11; Kahana; 08 Jul 1928; O. Oswald leg.; BPBM 91314 • 12; Kahana; 02 Jun 1928; O. Oswald leg.; BPBM 90935 • 12; Kahana; 08 Sep 1928; O. Oswald leg.; BPBM 91378 • 14; Kahana; 08 Sep 1928; O. Oswald leg.; BPBM 91384 • 16; Kahana; 08 Sep 1928; O. Oswald leg.; BPBM 91377 • 23; Kahana; 08 Sep 1928; O. Oswald leg.; BPBM 91374 • 23; Kahana; 03 Nov 1928; O. Oswald leg.; BPBM 92097 • 29; Kahana; 08 Sep 1928; O. Oswald leg.; BPBM 91390 • 32; Kahana; 22 Sep 1928; O. Oswald leg.; BPBM 91532 • 20; Kahana; 10 Jun 1928; O. Oswald leg.; BPBM 90987 • 20; Kahana; 10 Jun 1928; O. Oswald leg.; BPBM 90988 • 20; Kahana; 10 Jun 1928; O. Oswald leg.; BPBM 90999 • 1; Kahana; 16 Dec 1928; O. Oswald leg.; BPBM 92219 • 1; Kalihi Valley; 25 Mar 1928; O. Oswald leg.; BPBM 90244 • 2; Kalihi Valley; 25 Mar 1928; O. Oswald leg.; BPBM 90245 • 2; Kalihi Valley; 25 Mar 1928; O. Oswald leg.; BPBM 90210 • 3; Kalihi Valley; 25 Mar 1928; O. Oswald leg.; BPBM 90259 • 3; Kalihi Valley; 25 Mar 1928; O. Oswald leg.; BPBM 90247 • 4; Kalihi Valley; 25 Mar 1928; O. Oswald leg.; BPBM 90239 • 4; Kalihi Valley; 25 Mar 1928; O. Oswald leg.; BPBM 90216 • 4; Kalihi Valley; 25 Mar 1928; O. Oswald leg.; BPBM 90233 • 5; Kalihi Valley; 25 Mar 1928; O. Oswald leg.; BPBM 90234 • 6; Kalihi Valley; 25 Mar 1928; O. Oswald leg.; BPBM 90220 • 7; Kalihi Valley; 25 Mar 1928; O. Oswald leg.; BPBM 90243 • 8; Kalihi Valley; 25 Mar 1928; O. Oswald leg.; BPBM 90253 • 8; Kalihi Valley; 25 Mar 1928; O. Oswald leg.; BPBM 90215 • 13; Kalihi Valley; 25 Mar 1928; O. Oswald leg.; BPBM 90240 • 16; Kalihi Valley; 25 Mar 1928; O. Oswald leg.; BPBM 90254 • 17; Kalihi Valley; 25 Mar 1928; O. Oswald leg.; BPBM 90238 • 4; Punawai; 01 Jul 1941; O. Oswald leg.; BPBM 194127 • 2; Kaluanui; 01 Jul 1941; O. Oswald leg.; BPBM 194105 • 2; Kaluanui; 01 Jul 1941; O. Oswald leg.; BPBM 194102 • 1; Kawailoa; 01 Jul 1941; O. Oswald leg.; BPBM 194157 • 7; Kawailoa; 01 Jul 1941; O. Oswald leg.; BPBM 194140 • 5; Kawailoa; 01 Jul 1941; O. Oswald leg.; BPBM 194163 • 5; Kawailoa; 01 Jul 1941; O. Oswald leg.; BPBM 194153 • 5; Punaluu; 01 Jul 1941; O. Oswald leg.; BPBM 194098 • 3; Kahana; 23 Sep 1928; O. Oswald leg.; BPBM 93544 • 22; Kaaawa; 11 Aug 1929; O. Oswald leg.; BPBM 96627 • 22; Kaaawa; 11 Aug 1929; O. Oswald leg.; BPBM 96639 • 22; Kaaawa; 11 Aug 1929; O. Oswald leg.; BPBM 96654 • 1; Kaaawa; 31 Jul 1929; O. Oswald leg.; BPBM 96586 • 1; Kaaawa; 31 Jul 1929; O. Oswald leg.; BPBM 96587 • 2; Kaaawa; 06 Oct 1929; O. Oswald leg.; BPBM 96762 • 6; Kaaawa; 06 Oct 1929; O. Oswald leg.; BPBM 96763 • 3; Kahana; 12 Jun 1929; O. Oswald leg.; BPBM 96575 • 11; Kahana; 12 Jun 1929; O. Oswald leg.; BPBM 96574 • 3; Puu Kaaumakua; 06 May 1929; O. Oswald leg.; BPBM 96453 • 5; Puu Kaaumakua; 06 May 1929; O. Oswald leg.; BPBM 96452 • 16; Puu Kaaumakua; 06 May 1929; O. Oswald leg.; BPBM 96451 • 1; Kahana-Waikane; 06 May 1929; O. Oswald leg.; BPBM 96437 • 3; Kahana-Waikane; 06 May 1929; O. Oswald leg.; BPBM 96466 • 3; Kahana-Waikane; 06 May 1929; O. Oswald leg.; BPBM 96442 • 5; Kahana-Waikane; 06 May 1929; O. Oswald leg.; BPBM 96441 • 13; Kahana-Waikane; 06 May 1929; O. Oswald leg.; BPBM 96440 • 2; Kaluanui; 15 Dec 1929; O. Oswald leg.; BPBM 96926 • 5; Kaluanui; 15 Dec 1929; O. Oswald leg.; BPBM 96927 • 1; Manana; 20 Oct 1929; O. Oswald leg.; BPBM 96815 • 2; Punaluu; 01 Dec 1929; O. Oswald leg.; BPBM 96865 • 8; Punaluu; 01 Dec 1929; O. Oswald leg.; BPBM 96851 • 8; Punaluu; 01 Dec 1929; O. Oswald leg.; BPBM 96860 • 4; Punaluu; 29 Sep 1929; O. Oswald leg.; BPBM 96730 • 11; Punaluu; 29 Sep 1929; O. Oswald leg.; BPBM 96741 • 9; Punaluu; 21 Sep 1929; O. Oswald leg.; BPBM 96706 • 12; Punaluu; 21 Sep 1929; O. Oswald leg.; BPBM 96707 • 6; Punaluu; 01 Dec 1929; O. Oswald leg.; BPBM 96868 • 4; Kipapa Ridge; 06 Apr 1929; O. Oswald leg.; BPBM 96334 • 16; Kipapa Ridge; 06 Apr 1929; O. Oswald leg.; BPBM 96333 • 3; Puu Ohulehule; 20 Apr 1930; O. Oswald leg.; BPBM 98019 • 3; Puu Ohulehule; 20 Apr 1930; O. Oswald leg.; BPBM 98016 • 5; Puu Ohulehule; 20 Apr 1930; O. Oswald leg.; BPBM 98018 • 6; Halawa; 02 Mar 1930; O. Oswald leg.; BPBM 97892 • 7; Halawa; 02 Mar 1930; O. Oswald leg.; BPBM 97893 • 3; Hauula-Punaluu; 09 Feb 1929; O. Oswald leg.; BPBM 92778 • 6; Hauula-Punaluu; 09 Feb 1929; O. Oswald leg.; BPBM 92783 • 3; Hauula-Punaluu; 09 Feb 1929; O. Oswald leg.; BPBM 92777 • 2; Kaaawa; 06 Jan 1929; O. Oswald leg.; BPBM 92692 • 6; Kaaawa; 06 Jan 1929; O. Oswald leg.; BPBM 92691 • 11; Kaaawa; 06 Jan 1929; O. Oswald leg.; BPBM 92680 • 20; Kaaawa; 06 Jan 1929; O. Oswald leg.; BPBM 92679 • 20; Kaaawa; 06 Jan 1929; O. Oswald leg.; BPBM 92690 • 2; Weliweli; 16 Nov 1930; O. Oswald leg.; BPBM 100913 • 20; Weliweli; 16 Nov 1930; O. Oswald leg.; BPBM 100914 • 20; Weliweli; 23 Nov 1930; O. Oswald leg.; BPBM 100919 • 20; Weliweli; 23 Nov 1930; O. Oswald leg.; BPBM 100920 • 7; Ahuimanu; 21 Dec 1931; O. Oswald leg.; BPBM 104378 • 7; Ahuimanu; 21 Dec 1931; O. Oswald leg.; BPBM 104379 • 14; Ahuimanu; 21 Dec 1931; O. Oswald leg.; BPBM 104377 • 1; Kahana; 27 Jan 1929; O. Oswald leg.; BPBM 92742 • 1; Kahana; 03 Feb 1929; O. Oswald leg.; BPBM 92758 • 1; Kahana; 18 May 1930; O. Oswald leg.; BPBM 99041 • 2; Kahana; 22 Jan 1929; O. Oswald leg.; BPBM 92720 • 2; Kahana; 18 May 1930; O. Oswald leg.; BPBM 99054 • 3; Kahana; 27 Jan 1929; O. Oswald leg.; BPBM 92725 • 4; Kahana; 18 May 1930; O. Oswald leg.; BPBM 99053 • 9; Kahana; 27 Jan 1929; O. Oswald leg.; BPBM 92724 • 13; Kahana; 18 May 1930; O. Oswald leg.; BPBM 99052 • 22; Kahana; 22 Jan 1929; O. Oswald leg.; BPBM 92719 • 22; Kahana; 27 Jan 1929; O. Oswald leg.; BPBM 92732 • 22; Kahana; 27 Jan 1929; O. Oswald leg.; BPBM 92733 • 22; Kahana; 27 Jan 1929; O. Oswald leg.; BPBM 92734 • 22; Kahana; 03 Feb 1929; O. Oswald leg.; BPBM 92768 • 0; Kahana; 18 May 1930; O. Oswald leg.; BPBM 99035 • 2; Kahana; 18 May 1930; O. Oswald leg.; BPBM 99030 • 9; Kahana; 18 May 1930; O. Oswald leg.; BPBM 99080 • 15; Kahana; 18 May 1930; O. Oswald leg.; BPBM 99036 • 5; Kahana; 18 May 1930; O. Oswald leg.; BPBM 99063 • 5; Kahana; 18 May 1930; O. Oswald leg.; BPBM 99064 • 19; Kahuku; 05 Apr 1931; O. Oswald leg.; BPBM 104419 • 19; Kahuku; 05 Apr 1931; O. Oswald leg.; BPBM 104420 • 19; Kahuku; 05 Apr 1931; O. Oswald leg.; BPBM 104421 • 2; Kalihi; 12 Apr 1930; O. Oswald leg.; BPBM 97983 • 1; Mauna Kope; 01 Sep 1930; O. Oswald leg.; BPBM 99595 • 21; Mauna Kope-Moanalua; 22 Mar 1930; O. Oswald leg.; BPBM 97922 • 21; Mauna Kope-Moanalua; 22 Mar 1930; O. Oswald leg.; BPBM 97923 • 1; Kaliuwaa; 09 Nov 1930; O. Oswald leg.; BPBM 100888 • 5; Moanalua; 16 Mar 1930; O. Oswald leg.; BPBM 97907 • 3; Moanalua; 01 Jun 1930; O. Oswald leg.; BPBM 99133 • 4; Moanalua; 01 Jun 1930; O. Oswald leg.; BPBM 99134 • 21; Moanalua; 16 Mar 1930; O. Oswald leg.; BPBM 97921 • 1; Puu Peahinaia; 03 Aug 1930; O. Oswald leg.; BPBM 99458 • 1; Punaluu; 05 Oct 1930; O. Oswald leg.; BPBM 100695 • 1; Punaluu; 26 Jan 1930; O. Oswald leg.; BPBM 97798 • 3; Punaluu; 05 Oct 1930; O. Oswald leg.; BPBM 100693 • 4; Punaluu; 05 Oct 1930; O. Oswald leg.; BPBM 100694 • 1; Castle Trail; 05 Oct 1930; O. Oswald leg.; BPBM 100675 • 4; Castle Trail; 05 Oct 1930; O. Oswald leg.; BPBM 100677 • 9; Castle Trail; 05 Oct 1930; O. Oswald leg.; BPBM 100676 • 1; Punaluu; 12 Jan 1930; O. Oswald leg.; BPBM 97779 • 4; Punaluu; 12 Jan 1930; O. Oswald leg.; BPBM 97778 • 5; Punaluu; 12 Jan 1930; O. Oswald leg.; BPBM 97777 • 8; Punaluu; 12 Jan 1930; O. Oswald leg.; BPBM 97776 • 2; Pupukea; 17 Feb 1931; O. Oswald leg.; BPBM 104406 • 2; Pupukea; 17 Feb 1931; O. Oswald leg.; BPBM 104407 • 5; Pupukea; 17 Feb 1931; O. Oswald leg.; BPBM 104408 • 7; Pupukea; 17 Feb 1931; O. Oswald leg.; BPBM 104398 • 7; Pupukea; 17 Feb 1931; O. Oswald leg.; BPBM 104399 • 16; Pupukea; 17 Feb 1931; O. Oswald leg.; BPBM 104400 • 3; Waiahole; 26 Apr 1931; O. Oswald leg.; BPBM 105183 • 3; Waiahole; 19 Apr 1931; O. Oswald leg.; BPBM 104430 • 4; Waiahole; 26 Apr 1931; O. Oswald leg.; BPBM 105182 • 4; Waiahole; 19 Apr 1931; O. Oswald leg.; BPBM 104431 • 9; Waikane; 27 Apr 1930; O. Oswald leg.; BPBM 98069 • 11; Waikane; 27 Apr 1930; O. Oswald leg.; BPBM 98068 • 2; Waimea; 10 May 1931; O. Oswald leg.; BPBM 105210 • 18; Waimea; 10 May 1931; O. Oswald leg.; BPBM 105209 • 1; Kalihi Ridge; 11 Feb 1907; P.H. Timberlake (?) leg.; BPBM 42897 • 4; Kaliuwaa; 29 Sep 1912; R. von Holt, C.M. Cooke III, Cooke leg.; BPBM 24925 • 3; Laie; 24 Jul 1938; R. Yamaguchi, Yoshio Kondo leg.; BPBM 180548 • 6; Laie; 24 Jul 1938; R. Yamaguchi, Yoshio Kondo leg.; BPBM 180547 • 14; Kaipapau; R.A., C.M. Cooke leg.; BPBM 21480 • 128; Kaipapau; R.A., C.M. Cooke leg.; BPBM 21467 • 45; Konahuanui Ridge; 22 Jul 1910; R.A., C.M. Cooke leg.; BPBM 21461 • 136; Papali-Punaiki; 20 Dec 1932; Russ, Glen W. leg.; BPBM 134333 • 43; Kaunala-Oio; 13 Jun 1933; Russ, Glen W. leg.; BPBM 134280 • 4; Ihiihi Gulch; 01 Jan 1929; Russ, Glen W. leg.; BPBM 134298 • 3; Ihiihi Valley; 07 Nov 1931; Russ, Glen W. leg.; BPBM 107598 • 59; Makao; 20 Jun 1933; Russ, Glen W. leg.; BPBM 134341 • 9; Castle Trail; 22 Dec 1932; Russ, Glen W. leg.; BPBM 134358 • 20; Castle Trail; 27 Jun 1933; Russ, Glen W. leg.; BPBM 134365 • 10; Punaluu; 20 Nov 1931; Russ, Glen W. leg.; BPBM 134366 • 5; Kipapa-Waikakalaua; 09 Dec 1931; Russ, Glen W. leg.; BPBM 134169 • 2; Hauula; 01 Sep 1915; Spalding leg.; BPBM 41264 • 6; Hauula; 01 Nov 1914; Spalding leg.; BPBM 37857 • 6; Hauula; 22 Nov 1914; Spalding leg.; BPBM 37864 • 11; Hauula; 10 Jun 1917; Spalding leg.; BPBM 43719 • 1; Kaaawa; 16 Apr 1911; Spalding leg.; BPBM 22308 • 12; Kaaawa; 22 Jan 1908; Spalding leg.; BPBM 15955 • 1; Ahuimanu; 10 Jun 1917; Spalding leg.; BPBM 43713 • 2; Kahana; Spalding leg.; BPBM 15969 • 38; Kahauiki; Spalding leg.; BPBM 17936 • 5; Kahauiki; Spalding leg.; BPBM 20831 • 19; Kahuku-Waialee; 24 Sep 1911; Spalding leg.; BPBM 23135 • 16; Kaipapau; Spalding leg.; BPBM 21569 • 2; Kamanaiki; 28 May 1916; Spalding leg.; BPBM 41905 • 1; Kaliuwaa; Spalding leg.; BPBM 17433 • 2; Laie-Malaekahana; 11 Jun 1911; Spalding leg.; BPBM 22752 • 1; Moanalua; Spalding leg.; BPBM 19166 • 1; Wahiawa; Spalding leg.; BPBM 23895 • 12; Waiahole; Spalding leg.; BPBM 21360 • 45; Waialee; 18 Oct 1914; Spalding leg.; BPBM 37361 • 3; Waimea; Spalding leg.; BPBM 22710 • 3; Waipio; 05 Jul 1908; Spalding leg.; BPBM 16717 • 15; Kipapa; 24 Dec 1911; Spalding leg.; BPBM 23555 • 3; Waipio; 08 May 1910; Spalding leg.; BPBM 20946 • 12; Kahana; 08 Oct 1933; Spalding, Irwin leg.; BPBM 118550 • 22; Kahana; 08 Oct 1933; Spalding, Irwin leg.; BPBM 118551 • 4; Kahuku; 06 Mar 1932; St John, Harold leg.; BPBM 108779 • 9; Kipapa-Waiawa; 06 Sep 1933; Stewart, Wendell O. leg.; BPBM 119969 • 24; Punaluu; 26 Oct 1941; T. Yamaguchi, Yoshio Kondo leg.; BPBM 211963 • 10; Kaaawa; 01 Mar 1913; Thaanum leg.; BPBM 36785 • 3; Laie; Thurston leg.; BPBM 22146 • 3; Kahuku-Malaekahana; 01 Feb 1910; Thurston, Lorrin A. leg.; BPBM 130982 • 713; Kaliuwaa; Tomo, Cooke leg.; BPBM 21540 • 1; Kahana; 20 May 1923; W.H. Meinecke leg.; BPBM 54575 • 5; Kahana; 20 May 1923; W.H. Meinecke leg.; BPBM 54574 • 11; Kahana; 20 May 1923; W.H. Meinecke leg.; BPBM 54573 • 80; Punaluu; W.J. Semmes leg.; BPBM 186977 • 34; Ihiihi-Kahawainui; 10 Apr 1935; Welch, D'Alte A. leg.; BPBM 132183 • 7; Hauula; 18 Feb 1925; W.H. Meinecke leg.; BPBM 167736 • 9; Kahana; 20 May 1923; W.H. Meinecke leg.; BPBM 167733 • 32; Kahana-Punaluu; 06 Mar 1932; W.H. Meinecke leg.; BPBM 167789 • 6; Kahana-Wahiawa; 15 Apr 1928; W.H. Meinecke leg.; BPBM 167841 • 10; Kahana-Wahiawa; 15 Apr 1928; W.H. Meinecke leg.; BPBM 167840 • 1; Kawaiiki-Opaeula; 19 Apr 1936; W.H. Meinecke leg.; BPBM 166298 • 24; Kipapa Ridge; 07 Apr 1929; W.H. Meinecke leg.; BPBM 167745 • 1; Waikakalaua; 19 Jan 1936; W.H. Meinecke leg.; BPBM 166187 • 8; Hauula; 01 Jan 1922; Wm. D. Wilder leg.; BPBM 51481 • 18; Hauula; 01 Jan 1922; Wm. D. Wilder leg.; BPBM 51488 • 20; Waialee; 01 Jan 1922; Wm. D. Wilder leg.; BPBM 51479 • 11; Kaliuwaa Valley (Sacred Falls Valley); 02 Mar 1946; Yoshio Kondo leg.; BPBM 190815 • 7; Kaluanui; 02 Jun 1946; Yoshio Kondo leg.; BPBM 210995 • 26; Castle Trail; 02 Jun 1946; Yoshio Kondo leg.; BPBM 210989 • 1; Wahiawa; 30 May 1953; Yoshio Kondo leg.; BPBM 216884.

*Auriculella perversa* Cooke, 1915

USA – Honolulu County, Oahu, Koolau Mountains • 1; Halawa Ridge; 18 Apr 1930; Meinecke, William H. leg.; BPBM 98131 • 1; Halawa Ridge; 18 Apr 1930; Meinecke, William H. leg.; BPBM 98132 • 22; Halawa Ridge; 09 Feb 1930; Oswald, Olaf leg.; BPBM 97833 • 22; Halawa Ridge; 09 Feb 1930; Oswald, Olaf leg.; BPBM 97834 • 22; Halawa Ridge; 09 Feb 1930; Oswald, Olaf leg.; BPBM 97835 • 22; Halawa Ridge; 09 Feb 1930; Oswald, Olaf leg.; BPBM 97836 • 3; Halawa Valley; 02 Mar 1930; Meinecke, William H. leg.; BPBM 97744 • 21; Halawa Valley; Crampton, Spalding, Waterhouse, Cooke leg.; BPBM 20173 • 22; Halawa Valley; 09 Feb 1930; Oswald, Olaf leg.; BPBM 97830 • 7; Kaalakei; Meinecke, William H. leg.; BPBM 120133 • 5; Kalihi; 02 May 1928; Oswald, Olaf leg.; BPBM 90691 • 1; Kalihi; BPBM 180695 • 1; Kalihi; BPBM 180659 • 1; Kalihi; BPBM 180660 • 1; Kalihi; BPBM 103201 • 1; Kalihi; BPBM 103202 • 1; Kalihi; BPBM 103203 • 1; Kalihi; BPBM 183043 • 1; Kalihi-Kaneohe; BPBM 183058 • 1; Kalihi-Kaneohe; BPBM 183059 • 1; Kalihi-Kaneohe; BPBM 183060 • 7; Kalihi-Moanalua; BPBM 93501 • 1; Kamanaiki; BPBM 162375 • 6; Konahuanui; 20 Mar 1928; Oswald, Olaf leg.; BPBM 90151 • 16; Konahuinui-Mount Olympus; 03 Sept 1911; Cooke leg.; BPBM 23018 • 6; Kulepiamoa Ridge; 19 Jul 1939; Oliver Huddleston Emerson, Edwin H. Bryan Jr., Donald Anderson leg.; BPBM 183032 • 9; Kulepiamoa Ridge; 19 Jul 1939; Oliver Huddleston Emerson, Edwin H. Bryan Jr., Donald Anderson leg.; BPBM 183031 • 1; Kuliouou; 18 Jun 1911; Spalding leg.; BPBM 22768 • 1; Kuliouou; 06 May 1923; J.F.G. Stokes, M. Barton, M. Neal leg.; BPBM 54636 • 3; Kuliouou; 14 Apr 1916; A. Gouveia leg.; BPBM 41878 • 10; Kuliouou; 06 May 1923; Meinecke, William H. leg.; BPBM 167732 • 15; Kuliouou; 01 Jan 1922; Wm. D. Wilder leg.; BPBM 51390 • 22; Kuliouou; Spalding leg.; BPBM 19691 • 99; Kuliouou; 01 Jan 1922; Wm. D. Wilder leg.; BPBM 51387 • 132; Kuliouou; 18 Jun 1911; Spalding leg.; BPBM 22767 • 11; Kuliouou Ridge; 06 May 1923; Meinecke, William H. leg.; BPBM 54570 • 3; Kuliouou-Niu; 26 Jun 1919; Emerson, J.S. leg.; BPBM 103194 • 5; Kuliouou-Niu; 26 Jun 1919; L.L. Loofbourow leg.; BPBM 49035 • 21; Kuliouou-Niu; 08 Apr 1928; Meinecke, William H. leg.; BPBM 120145 • 22; Kuliouou-Niu; 08 Apr 1928; Oswald, Olaf leg.; BPBM 90333 • 22; Kuliouou-Niu; 08 Apr 1928; Oswald, Olaf leg.; BPBM 90341 • 22; Kuliouou-Niu; 08 Apr 1928; Oswald, Olaf leg.; BPBM 90325 • 22; Kuliouou-Niu; 08 Apr 1928; Oswald, Olaf leg.; BPBM 90324 • 2; Kulliouou-Niu; 05 Mar 1939; Meinecke, William H. leg.; BPBM 183277 • 1; Manaiki; BPBM 180668 • 1; Manaiki; BPBM 180802 • 1; Manaiki; BPBM 180803 • 77; Manoa; BPBM 45995 • 17; Manoa Cliff Trail; 23 Jan 1937; M., Donald Anderson leg.; BPBM 164148 • 70; Manoa Cliff Trail; 23 Jan 1937; M., Donald Anderson leg.; BPBM 164147 • 4; Manoa Valley; 07 Nov 1918; Mant leg.; BPBM 46159 • 6; Manoa Valley; 31 Jul 1910; Spalding leg.; BPBM 21559 • 15; Manoa Valley; 17 Jun 1918; C.F. Mant leg.; BPBM 46024 • 119; Manoa Valley; 01 Jun 1918; C.F. Mant leg.; BPBM 45995 • 3; Moanalua Valley; 16 Mar 1930; Oswald, Olaf leg.; BPBM 97905 • 16; Moanalua Valley; 16 Mar 1930; Oswald, Olaf leg.; BPBM 97904 • 2; Mount Olympus; 12 May 1918; J.C. Bridwell leg.; BPBM 46353 • 2; Mount Olympus; 03 Sept 1911; Spalding leg.; BPBM 23039 • 4; Mount Olympus; 12 May 1918; J.C. Bridwell leg.; BPBM 46356 • 5; Mount Olympus; 14 Apr 1908; Cooke leg.; BPBM 16297 • 11; Mount Olympus; 12 May 1918; J.C. Bridwell leg.; BPBM 46351 • 26; Mount Olympus; 01 Jan 1922; Wm. D. Wilder leg.; BPBM 51401 • 1; Mount Olympus; BPBM 169341 • 1; Mount Olympus; BPBM 194681 • 1; Mount Olympus; BPBM 194682 • 1; Nuuanu; BPBM 91030 • 2; Nuuanu; 30 Oct 1915; A. Gouveia leg.; BPBM 40467 • 2; Nuuanu; BPBM 90132 • 42; Nuuanu; BPBM 16372 • 89; Nuuanu; BPBM 42385 • 1; Nuuanu; BPBM 170315 • 2; Nuuanu Pali; 21 Mar 1943; Meinecke, William H. leg.; BPBM 189889 • 1; Nuuanu Ridge; 04 Jun 1903; Cooke leg.; BPBM 12796 • 3; Nuuanu Ridge; 22 Apr 1908; Cooke leg.; BPBM 16372 • 4; Nuuanu Ridge; 16 Jun 1903; Cooke leg.; BPBM 12800 • 13; Nuuanu Ridge; 01 Aug 1903; Cooke leg.; BPBM 12805 • 28; Nuuanu Ridge; 08 Jun 1903; Cooke leg.; BPBM 12798 • 2; Nuuanu Valley; Cooke, Ponsonby leg.; BPBM 23840 • 1; Nuuanu-Konahuanui; 20 Mar 1928; Oswald, Olaf leg.; BPBM 90154 • 2; Nuuanu-Konahuanui; 20 Mar 1928; Oswald, Olaf leg.; BPBM 90107 • 2; Nuuanu-Konahuanui; 20 Mar 1928; Oswald, Olaf leg.; BPBM 90119 • 3; Nuuanu-Konahuanui; 20 Mar 1928; Oswald, Olaf leg.; BPBM 90131 • 3; Nuuanu-Konahuanui; 20 Mar 1928; Oswald, Olaf leg.; BPBM 90097 • 5; Nuuanu-Konahuanui; 20 Mar 1928; Oswald, Olaf leg.; BPBM 90092 • 22; Nuuanu-Konahuanui; 20 Mar 1928; Oswald, Olaf leg.; BPBM 90141 • 22; Nuuanu-Konahuanui; 20 Mar 1928; Oswald, Olaf leg.; BPBM 90093 • 22; Nuuanu-Konahuanui; 20 Mar 1928; Oswald, Olaf leg.; BPBM 90094 • 22; Nuuanu-Konahuanui; 20 Mar 1928; Oswald, Olaf leg.; BPBM 90095 • 22; Nuuanu-Konahuanui; 20 Mar 1928; Oswald, Olaf leg.; BPBM 90096 • 4; Palolo; 06 Feb 1928; J.C. Bridwell leg.; BPBM 169342 • 1; Palolo Ridge; 16 May 1916; Spalding leg.; BPBM 41387 • 6; Palolo Ridge; 01 Apr 1919; J.C. Bridwell leg.; BPBM 47482 • 14; Pauoa; 04 Jan 1914; Spalding leg.; BPBM 35227 • 1; Pauoa Flats; 10 Apr 1928; Oswald, Olaf leg.; BPBM 90367 • 1; Pauoa Flats; 28 Aug 1912; R. Scudder leg.; BPBM 24908 • 1; Pauoa Flats; 28 Aug 1912; S.S. Emerson leg.; BPBM 24913 • 2; Pauoa Flats; 17 Jun 1917; J.C. Bridwell leg.; BPBM 43898 • 7; Pauoa Flats; 04 Jan 1914; Cooke leg.; BPBM 35553 • 9; Pauoa Flats; 28 Aug 1912; R. Scudder leg.; BPBM 24907 • 33; Pauoa Flats; Burroughs, Frear, Cooke leg.; BPBM 19424 • 1; Pauoa Flats; BPBM 103200 • 3; Pauoa-Tantalus; 10 Jun 1917; J.C. Bridwell leg.; BPBM 43862 • 4; Pauoa-Tantalus; 10 Jun 1917; J.C. Bridwell leg.; BPBM 43857 • 2; Puu Lanihuli; 09 Feb 1919; J.C. Bridwell leg.; BPBM 47161 • 5; Puu Lanihuli; 01 Sept 1911; Thurston leg.; BPBM 23123 • 1; Puu Lanihuli; BPBM 169343 • 2; Puu o Kona; 02 Nov 1930; Oswald, Olaf leg.; BPBM 100828 • 3; Puu O Kona; 13 Jul 1937; Edwin H. Bryan Jr. leg.; BPBM 164181 • 3; Puu O Kona; 13 Jul 1937; Edwin H. Bryan Jr. leg.; BPBM 164182 • 4; Puu o Kona; 02 Nov 1930; Oswald, Olaf leg.; BPBM 100829 • 6; Puu o Kona; 02 Nov 1930; Oswald, Olaf leg.; BPBM 100815 • 10; Puu o Kona; 11 Jun 1938; Olaf Oswald, E. Meadows, Donald Anderson leg.; BPBM 180573 • 12; Puu o Kona; 13 Jun 1937; Donald Anderson leg.; BPBM 164216 • 15; Puu O Kona; 13 Jul 1937; Edwin H. Bryan Jr. leg.; BPBM 164180 • 20; Puu o Kona; 13 Jun 1937; Donald Anderson leg.; BPBM 164215 • 1; Tantalus; 20 May 1913; J.S. Emerson leg.; BPBM 34079 • 1; Tantalus; 04 Apr 1914; J.S. Emerson leg.; BPBM 36236 • 1; Tantalus; 27 Dec 1918; A. Gouveia leg.; BPBM 46257 • 2; Tantalus; 20 May 1913; J.S. Emerson leg.; BPBM 34071 • 12; Tantalus; 10 Dec 1907; Cooke leg.; BPBM 15905 • 13; Tantalus; 27 Dec 1918; A. Gouveia leg.; BPBM 46256 • 1; Tantalus; BPBM 103195 • 1; Tantalus; BPBM 103196 • 1; Tantalus; BPBM 103197 • 1; Tantalus; BPBM 103199 • 1; Tantalus; BPBM 103198 • 1; Tantalus; BPBM 133960 • 1; Tantalus Valley; J.S. Emerson leg.; BPBM 23946 • 153; Tantalus Valley; 01 Mar 1908; Cooke leg.; BPBM 16192 • 1; Tantalus-Pauoa Flats; 17 Jun 1917; J.C. Bridwell leg.; BPBM 43892 • 3; Wailupe; 01 Jan 1922; Wm. D. Wilder leg.; BPBM 51398 • 1; Waimanalo; BPBM 164228 • 1; Waimanalo; BPBM 164229.

*Auriculella tenella* Ancey, 1889

USA – Honolulu County, Oahu, Waianae Mountains • 1; Palehua Valley; 27 Dec 1914; Alice T. Cooke, C.M. Cooke leg.; BPBM 38018 • 1; Kukuiula; 09 Oct 1934; Bryan leg.; BPBM 129528 • 1; Mount Kaala; 21 Apr 1929; Bryan, Neal, Whiting leg.; BPBM 93651 • 1; Leilehua Valley; 23 Aug 1904; Cooke, Charles Montague Jr. leg.; BPBM 42419 • 1; Leilehua Valley; 23 Aug 1904; Cooke, Charles Montague Jr. leg.; BPBM 42420 • 1; Palehua Ridge; Cooke, Charles Montague Jr. leg.; BPBM 16947 • 1; Palehua Ridge; 16 Oct 1912; Cooke, Charles Montague Jr. leg.; BPBM 33088 • 1; Popouwela Glen; Cooke, Charles Montague Jr. leg.; BPBM 35298 • 1; Waianae; Cooke, Hilda, R. von Holt leg.; BPBM 21928 • 1; Palikea Ridge; 12 Aug 1931; Cooke, Welch leg.; BPBM 105956 • 1; Waianae; 13 Aug 1931; Cooke, Welch leg.; BPBM 106079 • 1; Waianae; 13 Aug 1931; Cooke, Welch leg.; BPBM 106177 • 1; Ekahanui Gulch; 22 Apr 1936; D'Alte A. Welch leg.; BPBM 177333 • 1; Mount Kaala; 28 Apr 1935; Elwood C. Zimmerman leg.; BPBM 162465 • 1; Palikea Ridge; 11 Nov 1936; Elwood C. Zimmerman leg.; BPBM 164312 • 1; Palikea Ridge; 11 Nov 1936; Elwood C. Zimmerman leg.; BPBM 164313 • 1; Palikea Ridge; 11 Nov 1936; Elwood C. Zimmerman leg.; BPBM 164314 • 1; Mount Kaala; 08 Dec 1935; F. Raymond Fosberg leg.; BPBM 162012 • 1; Mount Kaala; 28 Mar 1937; F. Raymond Fosberg leg.; BPBM 162723 • 1; Mount Kaala; 27 Mar 1937; F. Raymond Fosberg, Donald Anderson leg.; BPBM 162928 • 1; Mount Kaala; 27 Mar 1937; F. Raymond Fosberg, M., D. Anderson leg.; BPBM 163074 • 1; Waianae; Forbes leg.; BPBM 20882 • 1; Waianae; 25 May 1948; Gordon Harrison leg.; BPBM 210980 • 1; Mount Kaala; 22 Jul 1917; J.C. Bridwell leg.; BPBM 43973 • 1; Mount Kaala; 22 Jul 1917; J.C. Bridwell leg.; BPBM 43982 • 1; Waianae; 06 Feb 1928; J.C. Bridwell leg.; BPBM 169345 • 1; Manuwai Gulch; 04 Nov 1932; Lemke and Welch leg.; BPBM 114311 • 1; Mount Kaala; 27 Mar 1937; M., D. Anderson leg.; BPBM 162953 • 1; Mount Kaala; 28 Mar 1937; M., D. Anderson leg.; BPBM 162815 • 1; Mount Kaala; 26 Mar 1937; M., D. Anderson leg.; BPBM 163127 • 1; Mount Kaala; 26 Mar 1937; M., D. Anderson leg.; BPBM 163128 • 1; Ekahanui Gulch; 04 Mar 1934; Meinecke, William H. leg.; BPBM 125176 • 1; Haleauau; 31 Dec 1926; Meinecke, William H. leg.; BPBM 123132 • 1; Huliwai Gulch; 04 Feb 1934; Meinecke, William H. leg.; BPBM 126146 • 1; Huliwai Gulch; 04 Feb 1934; Meinecke, William H. leg.; BPBM 126177 • 1; Huliwai Gulch; 04 Feb 1934; Meinecke, William H. leg.; BPBM 126178 • 1; Huliwai Gulch; 04 Feb 1934; Meinecke, William H. leg.; BPBM 126282 • 1; Kaaikukai-Palawai; 15 Nov 1925; Meinecke, William H. leg.; BPBM 123439 • 1; Nanakuli; 15 Apr 1934; Meinecke, William H. leg.; BPBM 127864 • 1; Napepeiauolelo; 25 Mar 1934; Meinecke, William H. leg.; BPBM 126926 • 1; Palawai Gulch; 15 Nov 1925; Meinecke, William H. leg.; BPBM 123373 • 1; Palawai Gulch; 30 Mar 1934; Meinecke, William H. leg.; BPBM 127438 • 1; Palawai Gulch; 08 Apr 1934; Meinecke, William H. leg.; BPBM 127677 • 1; Palawai Gulch; 30 Mar 1934; Meinecke, William H. leg.; BPBM 127391 • 1; Mount Kaala; 22 Jul 1928; Neal, M.C., E.H. Bryan leg.; BPBM 92519 • 1; Mount Kaala; 08 May 1923; O. Oswald leg.; BPBM 54190 • 1; Mount Kaala; 08 May 1923; O. Oswald leg.; BPBM 54197 • 1; Puu Kaua; 22 Jun 1924; O. Swezey leg.; BPBM 76925 • 1; Palehua Ridge; 31 Dec 1917; O.H.E. leg.; BPBM 103229 • 1; Palikea Ridge; 24 Aug 1922; R. von Holt, C.M. Cooke Jr., M.C. Neal leg.; BPBM 59563 • 1; Mount Kaala; 24 Jun 1937; R. Yamaguchi, Donald Anderson leg.; BPBM 163580 • 1; Mount Kaala; 25 Jun 1937; R. Yamaguchi, The Andersons leg.; BPBM 163653 • 1; Nanakuli; 14 Feb 1938; R. Yamaguchi, Yoshio Kondo, Others leg.; BPBM 172563 • 1; Nanakuli; 14 Feb 1938; R. Yamaguchi, Yoshio Kondo, Others leg.; BPBM 172564 • 1; Nanakuli; 14 Feb 1938; R. Yamaguchi, Yoshio Kondo, others leg.; BPBM 172620 • 1; Nanakuli; 14 Feb 1938; R. Yamaguchi, Yoshio Kondo, others leg.; BPBM 172621 • 1; Kupehau-Napepeiauolelo; 17 May 1933; Russ, Glen W. leg.; BPBM 133777 • 1; Leilehua Valley; Spalding leg.; BPBM 19744 • 1; Mount Kaala; Spalding leg.; BPBM 22814 • 1; Popouwela; 02 Mar 1913; Spalding leg.; BPBM 33876 • 1; Popouwela; 05 Nov 1911; Spalding leg.; BPBM 23399 • 1; Popouwela Ridge; 14 Sep 1913; Spalding leg.; BPBM 34819 • 1; Pukaloa; Spalding leg.; BPBM 20862 • 1; Waianae; 16 Nov 1913; Spalding leg.; BPBM 34846 • 1; Waianae; 13 Oct 1912; von Holt, Cooke leg.; BPBM 33038 • 1; Popouwela Valley; 16 Aug 1932; Welch, D'Alte A., Christopherson leg.; BPBM 131715 • 1; Mohiakea-Pukaloa; 22 Jan 1933; Winne, Welch leg.; BPBM 117105 • 1; Nanakuli; 05 Sep 1940; Yoshio Kondo, Donald Anderson leg.; BPBM 186868 • 2; Palehua Ridge; 23 Aug 1922; C.M. Cooke Jr., M.C. Neal leg.; BPBM 59314 • 2; Palehua Ridge; 25 Aug 1922; C.M. Cooke Jr., M.C. Neal leg.; BPBM 59655 • 2; Palikea Ridge; 27 Dec 1914; C.M.C. leg.; BPBM 38050 • 2; Lualualei; 15 Jun 1932; Christophersen, Welch leg.; BPBM 113714 • 2; Palehua Ridge; 06 Mar 1903; Cooke, Charles Montague Jr. leg.; BPBM 12765 • 2; Palehua Ridge; Cooke, Charles Montague Jr. leg.; BPBM 16849 • 2; Palehua Valley; Cooke, Charles Montague Jr. leg.; BPBM 21911 • 2; Nanakuli; 12 Aug 1931; Cooke, Welch leg.; BPBM 105733 • 2; Nanakuli; 12 Aug 1931; Cooke, Welch leg.; BPBM 105631 • 2; Palikea Ridge; 12 Aug 1931; Cooke, Welch leg.; BPBM 105881 • 2; Waianae; 13 Aug 1931; Cooke, Welch leg.; BPBM 106080 • 2; Puumaialau Gulch; 16 Sep 1941; Donald Anderson leg.; BPBM 211768 • 2; Mount Kaala; 21 Sep 1937; Elwood C. Zimmerman leg.; BPBM 163947 • 2; Mount Kaala; 26 Mar 1937; F. Raymond Fosberg, M., D. Anderson leg.; BPBM 162733 • 2; Mount Kaala; 26 Mar 1937; F. Raymond Fosberg, M., D. Anderson leg.; BPBM 162739 • 2; Mount Kaala; 26 Mar 1937; F. Raymond Fosberg, M., D. Anderson leg.; BPBM 162740 • 2; Mount Kaala; 24 Jun 1937; M. Anderson, R. Yamaguchi leg.; BPBM 163525 • 2; Haleauau; 26 Mar 1937; M., D. Anderson leg.; BPBM 163191 • 2; Mount Kaala; 27 Mar 1937; M., D. Anderson leg.; BPBM 163064 • 2; Mount Kaala; 28 Mar 1937; M., D. Anderson leg.; BPBM 162810 • 2; Mount Kaala; 28 Mar 1937; M., D. Anderson leg.; BPBM 162826 • 2; Mount Kaala; 28 Mar 1937; M., D. Anderson leg.; BPBM 162828 • 2; Mount Kaala; 28 Mar 1937; M., D. Anderson leg.; BPBM 162849 • 2; Palehua Ridge; 15 Nov 1925; Meinecke, William H. leg.; BPBM 93030 • 2; Ekahanui-Huliwai; 17 Dec 1933; Meinecke, William H. leg.; BPBM 125076 • 2; Huliwai Gulch; 11 Feb 1934; Meinecke, William H. leg.; BPBM 126469 • 2; Kaaikukai; 18 Mar 1934; Meinecke, William H. leg.; BPBM 126752 • 2; Palehua Ridge; 15 Nov 1925; Meinecke, William H. leg.; BPBM 123428 • 2; Lualualei; 11 Oct 1928; O. Oswald leg.; BPBM 91886 • 2; Popouwela Ridge; 16 Mar 1913; Pils, Spalding, Cooke leg.; BPBM 33759 • 2; Palehua Valley; 19 Oct 1912; R. von Holt, Cooke leg.; BPBM 33158 • 2; Halona-Palawai; 05 May 1936; R. Yamaguchi, D'Alte A. Welch leg.; BPBM 177498 • 2; Mount Kaala; 25 Jun 1937; R. Yamaguchi, The Andersons leg.; BPBM 163652 • 2; Nanakuli; 14 Feb 1938; R. Yamaguchi, Yoshio Kondo, Others leg.; BPBM 172562 • 2; Huliwai Gulch; 14 May 1933; Russ, Glen W. leg.; BPBM 133614 • 2; Huliwai Gulch; 14 May 1933; Russ, Glen W. leg.; BPBM 133660 • 2; Haleauau; 14 Aug 1914; Spalding leg.; BPBM 36621 • 2; Mauna Kapu; Spalding leg.; BPBM 20190 • 2; Palikea Ridge; 14 Jun 1908; Spalding leg.; BPBM 16652 • 2; Napepeiauolelo; 03 Apr 1938; William H. Meinecke, E. Meadows, Donald Anderson leg.; BPBM 173926 • 2; Kaluaa Gulch; 24 Nov 1946; Yoshio Kondo leg.; BPBM 211162 • 2; Puu Kaua; 16 Feb 1947; Yoshio Kondo, Others leg.; BPBM 210428 • 2; Nanakuli; 12 Aug 1939; Yoshio, Kay Kondo leg.; BPBM 183332 • 3; Waianae; BPBM 11021 • 3; Halona; 27 Apr 1932; Burrows, Welch leg.; BPBM 113188 • 3; Lualualei; 15 Jun 1932; Christophersen, Welch leg.; BPBM 113678 • 3; Palikea Ridge; Cooke, Charles Montague Jr. leg.; BPBM 16924 • 3; Ekahanui Gulch; 16 Sep 1941; Donald Anderson leg.; BPBM 211749 • 3; Mount Kaala; 11 Apr 1948; Dybas, Yoshio Kondo leg.; BPBM 211140 • 3; Mount Kaala; 24 Jun 1937; M. Anderson, R. Yamaguchi leg.; BPBM 163523 • 3; Mount Kaala; 28 Mar 1937; M., D. Anderson leg.; BPBM 163089 • 3; Mount Kaala; 28 Mar 1937; M., D. Anderson leg.; BPBM 163090 • 3; Mount Kaala; 28 Mar 1937; M., D. Anderson leg.; BPBM 162863 • 3; Ekahanui Gulch; 07 Jan 1934; Meinecke, William H. leg.; BPBM 125607 • 3; Ekahanui Gulch; 07 Apr 1915; Meinecke, William H. leg.; BPBM 123337 • 3; Huliwai-Waieli; 11 Mar 1934; Meinecke, William H. leg.; BPBM 126662 • 3; Palawai Gulch; 30 Mar 1934; Meinecke, William H. leg.; BPBM 127393 • 3; Puu Kaua; 22 Jun 1924; O. Swezey leg.; BPBM 76924 • 3; Palawai Gulch; 12 May 1936; Otto Degener, D'Alte A. Welch leg.; BPBM 165152 • 3; Haleauau; Spalding leg.; BPBM 17645 • 3; Pukaloa; 19 Jan 1919; William H. Meinecke leg.; BPBM 167822 • 4; Palaiki Valley; Cooke, Charles Montague Jr. leg.; BPBM 16839 • 4; Palehua Valley; Cooke, Charles Montague Jr. leg.; BPBM 16899 • 4; Pukaloa; 23 Aug 1904; Cooke, Charles Montague Jr. leg.; BPBM 12769 • 4; Waianae; Cooke, Charles Montague Jr. leg.; BPBM 16885 • 4; Palikea Ridge; 12 Aug 1931; Cooke, Welch leg.; BPBM 105955 • 4; Waianae; 13 Aug 1931; Cooke, Welch leg.; BPBM 106078 • 4; Nanakuli; 15 Jun 1939; Donald Anderson, R. Yamaguchi leg.; BPBM 182754 • 4; Palawai Gulch; 14 Jun 1939; Donald Anderson, R. Yamaguchi leg.; BPBM 182653 • 4; Palawai Gulch; 14 Jun 1939; Donald Anderson, R. Yamaguchi leg.; BPBM 182654 • 4; Nanakuli; 15 Apr 1934; Meinecke, William H. leg.; BPBM 127815 • 4; Lualualei; 11 Oct 1928; O. Oswald leg.; BPBM 91899 • 4; Popouwela Ridge; 16 Mar 1913; Pils, Spalding, Cooke leg.; BPBM 33780 • 4; Palikea Ridge; 12 Oct 1912; R. von Holt, Cooke leg.; BPBM 24990 • 4; Mohiakea-Pukaloa; 22 Mar 1936; Robert L. Usinger leg.; BPBM 165883 • 4; Lihue Valley; 30 May 1913; Spalding leg.; BPBM 34090 • 4; Palikea Ridge; Spalding leg.; BPBM 22737 • 4; Popouwela; 02 Mar 1913; Spalding leg.; BPBM 33875 • 4; Mount Kaala; 10 Apr 1948; Yoshio Kondo leg.; BPBM 211147 • 5; Nanakuli; 12 Aug 1931; Cooke, Welch leg.; BPBM 105656 • 5; Mount Kaala; 24 Jun 1937; M. Anderson, R. Yamaguchi leg.; BPBM 163524 • 5; Mount Kaala; 27 Mar 1937; M., D. Anderson leg.; BPBM 163065 • 5; Mount Kaala; 28 Mar 1937; M., D. Anderson leg.; BPBM 162864 • 5; Ekahanui Gulch; 25 Feb 1934; Meinecke, William H. leg.; BPBM 125233 • 5; Nanakuli; 22 Apr 1934; Meinecke, William H. leg.; BPBM 128020 • 5; Palehua Ridge; R. von Holt, Cooke leg.; BPBM 21846 • 5; Waianae; R. von Holt, Cooke leg.; BPBM 21832 • 5; Ekahanui Gulch; 16 Sep 1941; Rokuro Yamaguchi, Yoshio Kondo leg.; BPBM 211645 • 5; Nanakuli; 25 Aug 1941; Rokuro Yamaguchi, Yoshio Kondo leg.; BPBM 211349 • 5; Nanakuli; 29 Oct 1939; S. Tachikawa, Rokuro Yamaguchi, Yoshio Kondo leg.; BPBM 183532 • 5; Palikea Ridge; BPBM 21869 • 6; Popouwela Ridge; 30 Jul 1933; Cooke, Charles Montague Jr. leg.; BPBM 119114 • 6; Popouwela Valley; 23 Nov 1913; Cooke, Spalding leg.; BPBM 35431 • 6; Nanakuli; 15 Jun 1939; Donald Anderson, R. Yamaguchi leg.; BPBM 182875 • 6; Popouwela; 16 Aug 1932; E. Christopherson, D'Alte A. Welch leg.; BPBM 172721 • 6; Mount Kaala; 28 Mar 1937; M., D. Anderson leg.; BPBM 162830 • 6; Ekahanui Gulch; 14 Jan 1934; Meinecke, William H. leg.; BPBM 125641 • 6; Ekahanui Gulch; 07 Jan 1934; Meinecke, William H. leg.; BPBM 125578 • 6; Ekahanui Gulch; 25 Feb 1934; Meinecke, William H. leg.; BPBM 125340 • 6; Napepeiauolelo; 25 Mar 1934; Meinecke, William H. leg.; BPBM 127139 • 6; Lualualei; 11 Oct 1928; O. Oswald leg.; BPBM 91906 • 6; Lualualei; 11 Oct 1928; O. Oswald leg.; BPBM 91917 • 6; Nanakuli; 04 Mar 1928; O. Oswald leg.; BPBM 90001 • 6; Puu Kaua; 22 Jun 1924; O. Swezey leg.; BPBM 76923 • 6; Waianae; 06 Jun 1929; Oswald, O. leg.; BPBM 96536 • 6; Palikea Ridge; 24 Aug 1922; R. von Holt, C.M. Cooke Jr., M.C. Neal leg.; BPBM 59631 • 6; Popouwela Valley; 16 Aug 1932; Welch, D'Alte A., Christopherson leg.; BPBM 131728 • 6; Palikea Ridge; BPBM 16550 • 7; Nanakuli; 15 Jun 1939; Donald Anderson, R. Yamaguchi leg.; BPBM 182752 • 7; Ekahanui Gulch; 24 Jun 1936; Glen W. Russ, D'Alte A. Welch leg.; BPBM 177637 • 7; Puu Hapapa; 08 Mar 1948; J. Tobin, Yoshio Kondo leg.; BPBM 211062 • 7; Kaaikukai; 18 Mar 1934; Meinecke, William H. leg.; BPBM 126738 • 7; Nanakuli; 29 Apr 1934; Meinecke, William H. leg.; BPBM 128120 • 7; Waianae; 22 Apr 1934; Meinecke, William H. leg.; BPBM 127946 • 7; Ekahanui Gulch; 16 Sep 1941; Rokuro Yamaguchi, Yoshio Kondo leg.; BPBM 211724 • 7; Nanakuli; 25 Aug 1941; Rokuro Yamaguchi, Yoshio Kondo leg.; BPBM 211348 • 7; Lihue Valley; 16 Oct 1914; Spalding leg.; BPBM 37273 • 7; Puu Kaua; 03 May 1925; W.H. Meinecke leg.; BPBM 80020 • 8; Palehua Ridge; 08 Mar 1914; C.M. Cooke leg.; BPBM 35931 • 8; Popouwela Ridge; 30 Jul 1933; Cooke, Charles Montague Jr. leg.; BPBM 119115 • 8; Palikea Ridge; 12 Aug 1931; Cooke, Welch leg.; BPBM 105880 • 8; Nanakuli; 15 Jun 1939; Donald Anderson, R. Yamaguchi leg.; BPBM 182753 • 8; Mount Kaala; 24 Jun 1937; M. Anderson, R. Yamaguchi leg.; BPBM 163522 • 8; Ekahanui Gulch; 25 Feb 1934; Meinecke, William H. leg.; BPBM 125400 • 8; Napepeiauolelo; 01 Apr 1934; Meinecke, William H. leg.; BPBM 127559 • 8; Napepeiauolelo; 01 Apr 1934; Meinecke, William H. leg.; BPBM 127596 • 8; Palawai Gulch; 30 Mar 1934; Meinecke, William H. leg.; BPBM 127491 • 8; Palawai Gulch; 29 Apr 1934; Meinecke, William H. leg.; BPBM 128218 • 8; Palikea Ridge; 24 Aug 1922; R. von Holt, C.M. Cooke Jr., M.C. Neal leg.; BPBM 59562 • 8; Ekahanui Gulch; 16 Sep 1941; Rokuro Yamaguchi, Yoshio Kondo leg.; BPBM 211677 • 8; Napepeiauolelo-Palawai; BPBM 127635 • 9; Palikea Ridge; 12 Aug 1931; Cooke, Welch leg.; BPBM 106021 • 9; Waianae; Lyons, A.B. leg.; BPBM 103231 • 9; Mount Kaala; 28 Mar 1937; M., D. Anderson leg.; BPBM 162827 • 9; Huliwai Gulch; 11 Mar 1934; Meinecke, William H. leg.; BPBM 126632 • 9; Palawai Gulch; 30 Mar 1934; Meinecke, William H. leg.; BPBM 127392 • 9; Pualii; 28 Jan 1934; Meinecke, William H. leg.; BPBM 125982 • 9; Palikea Ridge; William Clench, Donald Anderson leg.; BPBM 186927 • 10; Palanui Valley-Palehua; Cooke, Charles Montague Jr. leg.; BPBM 16810 • 10; Waianae; 13 Aug 1931; Cooke, Welch leg.; BPBM 106128 • 10; Waianae; 12 Aug 1931; Cooke, Welch leg.; BPBM 105849 • 10; Nanakuli; 15 Jun 1939; Donald Anderson, R. Yamaguchi leg.; BPBM 182873 • 10; Nanakuli; 15 Jun 1939; Donald Anderson, R. Yamaguchi leg.; BPBM 182874 • 10; Mount Kaala; 26 Mar 1937; F. Raymond Fosberg, M., D. Anderson leg.; BPBM 162741 • 10; Puu Kaua; 22 Jun 1924; O. Swezey leg.; BPBM 76922 • 10; Palikea Ridge; 01 Apr 1938; William H. Meinecke, E. Meadows, Donald Anderson leg.; BPBM 183413 • 11; Palawai Gulch; 23 Aug 1922; C.M. Cooke Jr., M.C. Neal leg.; BPBM 59418 • 11; Palehua Valley; 07 Mar 1914; Cooke, Charles Montague Jr. leg.; BPBM 35851 • 11; Nanakuli; 12 Aug 1931; Cooke, Welch leg.; BPBM 105694 • 11; Waianae; D.D.B. leg.; BPBM 55850 • 11; Lualualei; 11 Oct 1928; O. Oswald leg.; BPBM 91907 • 11; Lualualei; 11 Oct 1928; O. Oswald leg.; BPBM 91918 • 11; Popouwela Ridge; 16 Mar 1913; Pils, Spalding, Cooke leg.; BPBM 33838 • 11; Popouwela Valley; 16 Aug 1932; Welch, D'Alte A., Christopherson leg.; BPBM 131690 • 12; Palehua Ridge; 23 Aug 1922; C.M. Cooke Jr., M.C. Neal leg.; BPBM 59334 • 12; Halona; 27 Apr 1932; Christophersen, Welch leg.; BPBM 113261 • 13; Ekahanui Gulch; 14 Jan 1934; Meinecke, William H. leg.; BPBM 125804 • 13; Huliwai Gulch; 11 Feb 1934; Meinecke, William H. leg.; BPBM 126361 • 13; Palikea Ridge; Spalding leg.; BPBM 21676 • 14; Palehua Ridge; 08 Mar 1914; C.M. Cooke leg.; BPBM 35915 • 14; Mount Kaala; 28 Mar 1937; M., D. Anderson leg.; BPBM 162865 • 14; Napepeiauolelo; 25 Mar 1934; Meinecke, William H. leg.; BPBM 126979 • 15; Popouwela; 14 Sep 1913; Cooke, Charles Montague Jr. leg.; BPBM 35281 • 15; Huliwai Gulch; 04 Feb 1934; Meinecke, William H. leg.; BPBM 126147 • 15; Waianae; 11 Mar 1934; Meinecke, William H. leg.; BPBM 126702 • 16; Palehua Ridge; 09 Nov 1919; Bryan, E.H. leg.; BPBM 103230 • 16; Puumaialau Gulch; 16 Sep 1941; Donald Anderson leg.; BPBM 211769 • 16; Mount Kaala; 28 Mar 1937; M., D. Anderson leg.; BPBM 162829 • 16; Puu Hapapa; 19 Mar 1948; Mr. and Mrs. Murnan, Yoshio Kondo leg.; BPBM 211096 • 16; Palikea Ridge; 24 Aug 1922; R. von Holt, C.M. Cooke Jr., M.C. Neal leg.; BPBM 59613 • 16; Ekahanui Gulch; 16 Sep 1941; Rokuro Yamaguchi, Yoshio Kondo leg.; BPBM 211725 • 16; Kupehau-Napepeiauolelo; 26 Jun 1933; Russ, Glen W. leg.; BPBM 133808 • 17; Palikea Ridge; 12 Aug 1931; Cooke, Welch leg.; BPBM 105879 • 17; Puu Hapapa; 08 Mar 1948; J. Tobin, Yoshio Kondo leg.; BPBM 211034 • 17; Puu Hapapa; 08 Mar 1948; J. Tobin, Yoshio Kondo leg.; BPBM 211035 • 17; Ekahanui Gulch; 21 Jan 1934; Meinecke, William H. leg.; BPBM 125910 • 17; Palawai Gulch; 18 Mar 1934; Meinecke, William H. leg.; BPBM 126875 • 17; Ekahanui-Puu Kanehoa; 01 Dec 1931; Russ, Glen W. leg.; BPBM 133720 • 18; Puu Kaua; 22 Jun 1924; O. Swezey leg.; BPBM 76921 • 19; Waianae; 12 Aug 1931; Cooke, Welch leg.; BPBM 105911 • 19; Nanakuli; 15 Jun 1939; Donald Anderson, R. Yamaguchi leg.; BPBM 182751 • 20; Popouwela; 16 Aug 1932; E. Christopherson, D'Alte A. Welch leg.; BPBM 172759 • 20; Ekahanui Gulch; 25 Feb 1934; Meinecke, William H. leg.; BPBM 125281 • 20; Palikea Ridge; R. von Holt, Cooke leg.; BPBM 21878 • 20; Ekahanui Gulch; 16 Sep 1941; Rokuro Yamaguchi, Yoshio Kondo leg.; BPBM 211505 • 21; Waianae; 12 Aug 1931; Cooke, Welch leg.; BPBM 105912 • 21; Waianae; Lyons, A.B. leg.; BPBM 109679 • 21; Ekahanui Gulch; 07 Jan 1934; Meinecke, William H. leg.; BPBM 125606 • 21; Leilehua Valley; BPBM 170337 • 22; Kupehau-Napepeiauolelo; 17 May 1933; Russ, Glen W. leg.; BPBM 133774 • 23; Leilehua Valley; Spalding leg.; BPBM 17054 • 23; Palikea Ridge; Spalding leg.; BPBM 22736 • 25; Palikea Ridge; 12 Aug 1931; Cooke, Welch leg.; BPBM 106020 • 25; Palehua Ridge; 09 Nov 1919; J.C. Bridwell leg.; BPBM 58635 • 25; Ekahanui-Puu Kanehoa; 01 Dec 1931; Russ, Glen W. leg.; BPBM 133701 • 26; Palehua Ridge; 09 Nov 1919; Bridwell leg.; BPBM 49187 • 27; Palehua Ridge; 09 Nov 1919; Bryan leg.; BPBM 49183 • 27; Nanakuli; 12 Aug 1931; Cooke, Welch leg.; BPBM 105735 • 27; Huliwai Gulch; 04 Feb 1934; Meinecke, William H. leg.; BPBM 126241 • 32; Nanakuli; 22 May 1938; William H. Meinecke, Donald Anderson leg.; BPBM 183500 • 34; Palikea Ridge; 15 Nov 1925; Meinecke, William H. leg.; BPBM 93314 • 36; Napepeiauolelo; 25 Mar 1934; Meinecke, William H. leg.; BPBM 127058 • 36; Palikea Ridge; 24 Aug 1922; R. von Holt, C.M. Cooke Jr., M.C. Neal leg.; BPBM 59506 • 38; Palikea Ridge; 12 Oct 1912; von Holt, Cooke leg.; BPBM 33019 • 39; Puu Kaua; 03 May 1925; W.H. Meinecke leg.; BPBM 80019 • 41; Ekahanui Gulch; 03 May 1925; Meinecke, William H. leg.; BPBM 123302 • 42; Palehua Ridge; 18 Oct 1912; Cooke, Charles Montague Jr. leg.; BPBM 33126 • 42; Ekahanui Gulch; 16 Sep 1941; Rokuro Yamaguchi, Yoshio Kondo leg.; BPBM 211472 • 43; Popouwela Ridge; 16 Mar 1913; Pils, Spalding, Cooke leg.; BPBM 33797 • 45; Mauna Kapu; 19 Oct 1912; R. von Holt, Cooke leg.; BPBM 33181 • 47; Palawai Gulch; 08 Apr 1934; Meinecke, William H. leg.; BPBM 127727 • 49; Palawai Gulch; 22 May 1938; William H. Meinecke, E. Meadows,, Donald Anderson leg.; BPBM 180933 • 56; Palehua Valley; 19 Oct 1912; R. von Holt, Cooke leg.; BPBM 33147 • 59; Nanakuli; 29 Apr 1934; Meinecke, William H. leg.; BPBM 128182 • 61; Napepeiauolelo; 25 Mar 1934; Meinecke, William H. leg.; BPBM 127220 • 64; Palehua Ridge; Cooke, Charles Montague Jr. leg.; BPBM 16998 • 70; Palawai Gulch; 23 Aug 1922; C.M. Cooke Jr., M.C. Neal leg.; BPBM 59476 • 70; Popouwela; 16 Aug 1932; E. Christopherson, D'Alte A. Welch leg.; BPBM 172722 • 72; Ekahanui Gulch; 07 Jan 1934; Meinecke, William H. leg.; BPBM 125476 • 78; Ekahanui Gulch; 14 Jan 1934; Meinecke, William H. leg.; BPBM 125718 • 82; Ekahanui Gulch; 07 Jan 1934; Meinecke, William H. leg.; BPBM 125539 • 89; Popouwela; 16 Aug 1932; Welch, D'Alte A., Christopherson leg.; BPBM 131947 • 96; Popouwela Valley-Waeli Gulch; 16 Aug 1932; Welch, D'Alte A., Christopherson leg.; BPBM 131767 • 98; Ekahanui Gulch; 16 Sep 1941; Rokuro Yamaguchi, Yoshio Kondo leg.; BPBM 211471 • 104; Nanakuli; 22 May 1938; William H. Meinecke, E. Meadows, Donald Anderson leg.; BPBM 183467 • 132; Napepeiauolelo; 03 Apr 1938; William H. Meinecke, E. Meadows, Donald Anderson leg.; BPBM 173981 • 139; Popouwela; 16 Aug 1932; Welch, D'Alte A., Christopherson leg.; BPBM 131815 • 160; Palikea Ridge; Cooke, Charles Montague Jr. leg.; BPBM 16908 • 181; Palikea Ridge; R. von Holt, Cooke leg.; BPBM 21869 • 185; Nanakuli; 15 Jun 1939; Donald Anderson, R. Yamaguchi leg.; BPBM 182901 • 187; Ekahanui Gulch; 16 Sep 1941; Rokuro Yamaguchi, Yoshio Kondo leg.; BPBM 211560 • 203; Lihue Valley; 16 Oct 1914; Cooke, Charles Montague Jr. leg.; BPBM 37206 • 214; Waianae; BPBM 33194 • 384; Popouwela; 16 Aug 1932; Welch, D'Alte A., Christopherson leg.; BPBM 131876.
